# Exploring the Phototherapeutic Applications of Mitochondria-Targeted COUPY Photocages of Antitumor Drugs

**DOI:** 10.1021/acs.jmedchem.5c00550

**Published:** 2025-04-28

**Authors:** Marta López-Corrales, Eduardo Izquierdo-García, Manel Bosch, Tapas Das, Amadeu Llebaria, Laia Josa-Culleré, Vicente Marchán

**Affiliations:** † Departament de Química Inorgànica i Orgànica, Secció de Química Orgànica, Universitat de Barcelona (UB), and Institut de Biomedicina de la Universitat de Barcelona (IBUB), Martí i Franquès 1-11, E-08028 Barcelona, Spain; ‡ Unitat de Microscòpia Òptica Avançada, Centres Científics i Tecnològics (CCiTUB), Universitat de Barcelona (UB), Av. Diagonal 643, E-08028 Barcelona, Spain; § Department of Chemistry, 230637 National Institute of Technology Jamshedpur, Jamshedpur, Jharkhand 831014, India; ∥ MCS, Department of Biological Chemistry, 203230 Institute for Advanced Chemistry of Catalonia (IQAC−CSIC), Jordi Girona 18-26, 08034 Barcelona, Spain

## Abstract

Photocleavable protecting groups hold great promise in photopharmacology to control the release of bioactive molecules from their caged precursors within specific subcellular compartments. Herein, we describe a series of photocages based on a COUPY scaffold, incorporating chlorambucil (CLB) and 4-phenylbutyric acid (4-PBA) as bioactive payloads that can be efficiently activated with visible light. Confocal microscopy confirmed the preferential accumulation of CLB and 4-PBA *N*-hexyl COUPY photocages in the mitochondria, which exhibited a remarkable phototoxicity against cancer cells upon green-yellow light irradiation, with IC_50_ values in the nanomolar range. This effect was attributed to a synergistic mechanism involving the photorelease of the bioactive payloads and the intrinsic photogeneration of Type I and Type II ROS by the COUPY scaffold within mitochondria. Thus, COUPY-caged derivatives of CLB and 4-PBA underscore the potential of COUPY-caging groups as a versatile platform to develop innovative light-activated agents operating simultaneously through photodynamic therapy and photoactivated chemotherapy.

## Graphical abstract

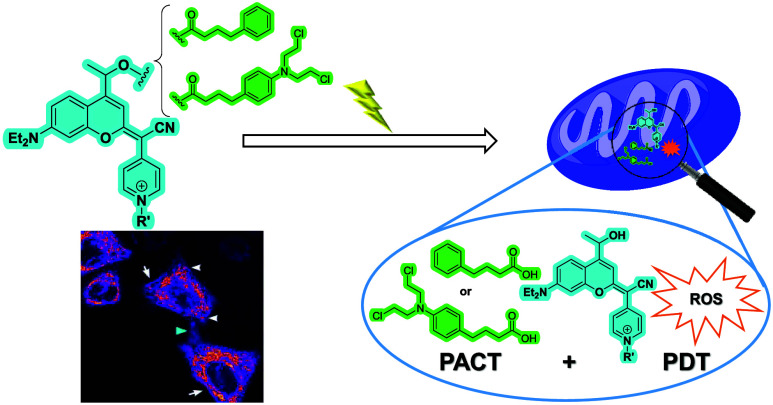

## Introduction

The use of photocleavable protecting groups (PPGs), also known as caging groups or photoremovable protecting groups, has emerged as a promising therapeutic strategy. By using light to control the spatial and temporal release of bioactive compounds, this approach enables targeted drug activation from light-responsive prodrugs (photocaged compounds) in a specific location, enhancing therapeutic selectivity and efficacy.^1–3^ In this context, the use of light in therapy, often called photopharmacology,^4–6^ has opened new possibilities in medicine for treating a wide variety of human diseases, including cancer. By minimizing the off-target activation of antitumor drugs in healthy tissues, this approach mitigates the side effects associated with conventional unspecific chemotherapeutic treatments.

Caged analogues of bioactive compounds, including small molecule drugs, peptides, oligonucleotides and neurotransmitters, can be prepared by temporally masking an essential functionality or motif required for their biological activity, with the appropriate PPG.^1–6^ Upon irradiation with light of the appropriate wavelength and intensity, the photolabile chemical bond is cleaved, triggering the release of the intact bioactive molecule at the targeted tissue (e.g., in a tumoral site), along with the corresponding PPG-derived photoproduct. To date, several classes of PPGs have been described in the literature, with most being activated by short-wavelength light. However, the use of UV or blue light is often impractical for many biological applications due to its inherent limitations. First, UV is highly phototoxic and can damage cellular components and biomolecules, such as DNA, proteins, and lipids, potentially leading to cell death, mutations, and an increased risk of carcinogenesis. Second, the poor penetration of short-wavelength light into tissues hinders its ability to effectively reach deep-seated tumors.^7,8^ To overcome these limitations, great efforts have been invested in developing PPGs activatable with longer wavelengths, specifically in the visible and near-infrared (NIR) region of the electromagnetic spectrum, commonly known as the optical window of biological tissues or phototherapeutic window, to achieve deeper tissue penetration and minimize phototoxicity.^9–12^ Among visible/NIR-light-sensitive PPGs based on organic chromophores, derivatives of boron dipyrromethene (BODIPY),^13–15^ coumarin,^16–19^ cyanine,^20,21^ naphthalene,^22,23^ and xanthenium^24^ scaffolds have been widely used across various fields. These systems enable more precise and safer therapeutic interventions, thereby broadening the scope and applicability of photopharmacology.

The development of fluorescent probes and PPGs that selectively target subcellular organelles such as mitochondria is particularly compelling given the critical roles this organelle plays in cellular homeostasis, signaling, and the progression of various diseases.^25–35^ Mitochondria are essential for energy production, but also play a vital role in a variety of biological processes related to cell growth and death, as well as regulating reactive oxygen species (ROS) levels, which can influence cellular health and pathology.^36^ Mitochondrial malfunction is implicated in several human pathologies, including cancer and neurodegenerative disorders, making it a critical target for disease treatment.^37,38^ In this context, many therapeutic approaches have been focused on targeting this key subcellular organelle by exploiting the negative membrane potential of the mitochondrial inner membrane. For instance, the attachment of positively charged peptides^39^ or lipophilic moieties such as triphenylphosphonium cations^40,41^ to bioactive molecules has been shown to enhance mitochondrial accumulation of the resulting conjugates. However, this approach has several limitations, since the incorporation of a peptide or a bulky hydrophobic group to the molecule of interest can alter its physicochemical and pharmacological properties.

Coumarin-based PPGs are particularly attractive in photopharmacology^42^ due to the structural versatility of the scaffold, which allows for the attachment of caged compounds through a variety of chemical linkages (e.g., ester, carbamate, carbonate, or thiocarbonate), and enables fine-tuning of the photophysical and photochemical properties.^17,43–45^ Our group has developed a new family of coumarin-based fluorophores, named COUPYs, that arise from the incorporation of a cyano­(1-alkyl-4-pyridin-1-ium)­methylene motif at position 2 of the coumarin backbone (e.g., compound **1**, Scheme 1). These dyes exhibit tunable photophysical properties upon minimal structural modifications and have been successfully used to fluorescently label biomolecules, such as peptides and lipids.^46–50^ COUPY fluorophores are also promising photosensitizers (PSs) for anticancer photodynamic therapy (PDT), either alone,^51^ nanoencapsulated^52^ or conjugated to Ir­(III) and Ru­(II) complexes,^53–55^ owing to their ability to simultaneously photogenerate Type I (e.g., superoxide) and Type II (singlet oxygen) ROS. Moreover, the replacement of the pyridinium moiety by bipyridine allowed the integration of coumarin-based ligands within the coordination sphere of Ru­(II) polypyridyl complexes, leading to PSs with outstanding *in vitro* and *in vivo* performance.^56^ Interestingly, COUPY dyes inherently accumulate in mitochondria owing to their positively charged *N*-alkylpyridinium moiety.^46–48^ This feature offers a valuable opportunity for targeted delivery of bioactive payloads to this crucial organelle when transformed into visible-light sensitive PPGs.^57,58^ Indeed, COUPY-based photocages (e.g., COUPY-caged model compound **2** in Scheme 1) can be photoactivated by yellow (560 nm) and red light (620 nm) under physiological-like conditions, while remaining stable to spontaneous hydrolysis when incubated in the dark in biologically relevant media.^57^ Importantly, the use of a COUPY-caged version of the protonophore 2,4-dinitrophenol (**3**, Scheme 1) allowed us to confirm, via confocal microscopy, that photoactivation can occur within mitochondria of living HeLa cells upon exposure to low doses of yellow light.^58^

**Scheme 1:**
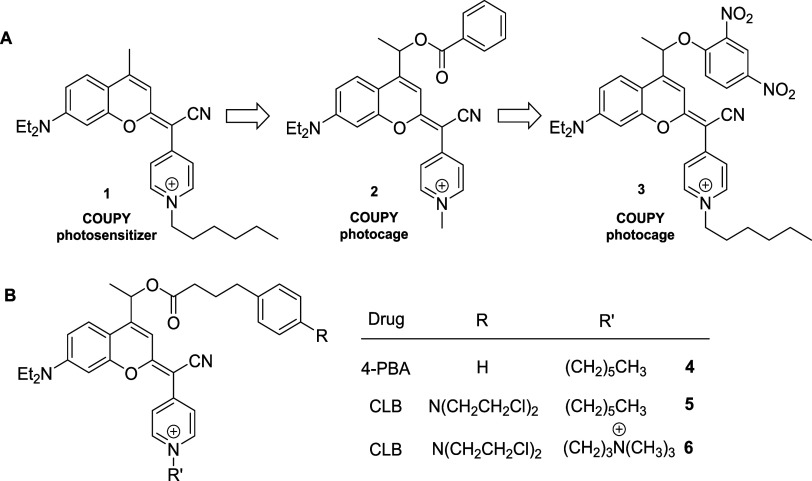
(A) Schematic Representation of the Transformation of COUPY Dyes into COUPY-Based Photocages. (B) Chemical Structure of COUPY Photocages **4-6**, Incorporating the Antitumor Drugs (CLB and 4-PBA) Investigated in This Work

Based on these precedents, in this work we describe a new class of light-activated anticancer agents based on COUPY photocages of two known chemotherapeutic drugs that combine the advantages of PDT and photoactivated chemotherapy (PACT) in a single molecule.^59,60^ We anticipate that the anticancer efficacy of these new dual-action PDT/PACT agents will be enhanced by the synergistic effect of the photoreleased cytotoxic payload and the ROS generated by the COUPY PPG or its coumarin photoproducts. We selected two antitumor drugs bearing a carboxylic acid group, namely chlorambucil (CLB) and 4-phenylbutyric acid (4-PBA), which enabled their incorporation into the COUPY-based PPG system via a photocleavable ester bond. On the one hand, CLB is an FDA-approved nitrogen mustard-based alkylating agent^61^ whose mechanism of action involves cross-linking the two complementary strands of DNA, preventing DNA replication and, thereby, inducing apoptosis in cancer cells. CLB is used in the clinics for the treatment of chronic lymphocytic leukemia, ovarian carcinoma, and lymphoma. On the other hand, 4-PBA is a histone deacetylase inhibitor^62^ being currently studied as a promising anticancer drug.^63–65^ In photocages **4** and **5** (Scheme 1), the pyridine moiety was alkylated with a hexyl chain, inspired by the excellent mitochondrial accumulation of the parent COUPY photosensitizer **1**.^51^ Additionally, CLB photocage **6**, modified with a (*N*,*N*,*N*-trimethylammonium)­propyl chain, was synthesized to investigate the effect of an extra positive charge on mitochondrial accumulation. Therefore, in this work we present the synthesis, photophysical, photochemical, and photobiological evaluation of 4-PBA (**4**) and CLB (**5, 6**) COUPY photocages as novel light-activated anticancer agents operating through a dual PDT/PACT mechanism.

## Results and Discussion

### Synthesis and Characterization of COUPY-Caged Compounds **4–6**

COUPY-photocages of CLB and 4-PBA (compounds **4–6**) were synthesized from coumarin alcohol **7**, which was obtained from commercially available 7-(*N,N*-diethylamino)-4-methylcoumarin, following previously published procedures developed by our group.^57,58^ First, esterification of **7** with 4-PBA using EDC as a coupling agent and DMAP as a catalyst provided compound **8**, which was transformed into thiocoumarin **9** by reaction with Lawesson’s reagent (LW) (Scheme 2). Then, condensation with 4-pyridylacetonitrile followed by a silver nitrate treatment afforded COUPY-derivative **10**, which was *N*-alkylated with 1-bromohexane to yield the COUPY photocage **4**. CLB photocages **5** and **6** were synthesized in two steps from the coumarin alcohol **11**, which was obtained by alkaline hydrolysis of coumarin **10**. On the one hand, compound **12** was obtained through alkylation of **11** with 1-bromohexane, followed by treatment with a large excess of KCl to obtain the pyridinium chloride salt. Then, esterification of CLB with **12** using EDC and DMAP afforded COUPY photocage **5**. Notably, preliminary attempts to perform the esterification reaction using the bromide salt of compound **12** resulted in the replacement of one of the Cl atoms with Br in the CLB moiety. On the other hand, the synthesis of COUPY photocage **6** involved first the esterification of CLB with coumarin alcohol **11**, followed by alkylation with (3-bromopropyl)­trimethylammonium bromide. In this case, the replacement of the Cl atom by Br in the CLB moiety could be reverted by subsequent treatment with Amberlite IRA-410 anion-exchange resin. All the compounds were purified by silica or alumina column chromatography and fully characterized by high-resolution mass spectrometry (HRMS) and 1D ^1^H and ^13^C­{^1^H} NMR spectroscopy. The purity of the compounds was assessed by HPLC-MS (Figure S1).

**Scheme 2:**
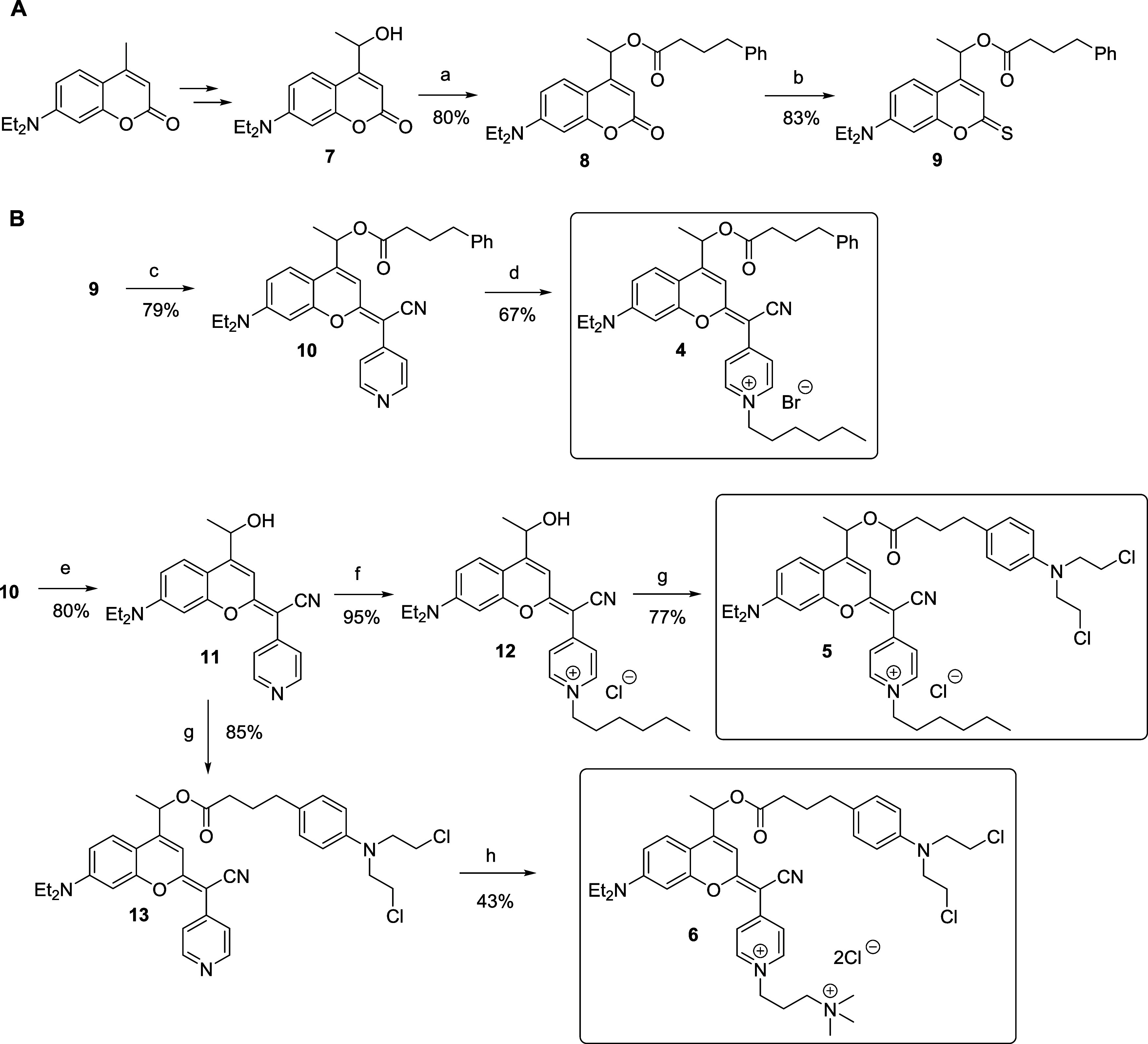
Synthetic Scheme Followed for the Preparation of COUPY-Caged Compounds **4-6** ^a^

a Reagents and Conditions: (a) 4-PBA, EDC.HCl, DMAP, DCM, RT, 18 h; (b) LW, toluene, 105 °C, 18 h; (c) (i) 4-pyridylacetonitrile hydrochloride, NaH, ACN anh., RT, 4 h, (ii) AgNO_3_, RT, 2 h; (d) 1-bromohexane, ACN anh., 60 °C, 18 h; (e) NaOH, ACN/H_2_O, RT, 18 h; (f) (i) 1-bromohexane, ACN anh., 60 °C, 18 h, (ii) KCl, RT, 4 h; (g) CLB, EDC.HCl, DMAP, DCM, RT, 18 h; (h), (i) Br­(CH_2_)_3_N­(CH_3_)_3_Br, KI, ACN anh., 80 °C, 72 h, (ii) Amberlite IRA-410, 60 °C, 18 h.

Interestingly, the 1D ^1^H NMR spectra of nonalkylated COUPY photocages of 4-PBA and CLB (**10** and **13**, respectively) showed two sets of proton signals in ∼ 90/92:10/8 ratios in DMSO-*d*_6_, which reproduced the results previously found with other COUPY fluorophores and photocages.^46–48,57,58^ The detection of chemical exchange cross-peaks in the ^1^H, ^1^H 2D-NOESY spectra confirmed the presence of a mixture of *E* and *Z* rotamers around the exocyclic carbon–carbon double bond (Figures S2–S3), with the *E* rotamer being identified as the predominant species. By contrast, only one set of proton signals was observed in the ^1^H NMR spectra of COUPY-caged compounds **4–6**. The presence of a NOESY cross-peak between the H8 of the coumarin backbone and the proton signals of the pyridinium moiety confirmed the *E*-configuration of the exocyclic backbone in these compounds (Figures S4–S6).

### Photophysical and Photochemical Characterization of the Compounds

The photophysical properties of COUPY-caged compounds (**4–6**) were studied in a mixture of PBS and ACN (8:2, v/v) at room temperature and compared with those of the coumarin alcohol **12** (Table 1). As shown in Figure 1, all compounds exhibited an intense absorption band in the visible region of the electromagnetic spectrum, with absorption maxima ranging from 563 nm (**4**-**5**) to 568 nm (**6**), which allowed the use of biocompatible visible light in the photobiological studies to be performed (*vide infra*). Esterification with both carboxylic acids (CLB or 4-PBA) caused a slight red shift in compounds **4–6** (*ca* 8–13 nm) relative to the parent coumarin alcohol **12**. On the other hand, the 5 nm red shift in the absorption maximum of compound **6** compared to compounds **4** and **5** indicates that the incorporation of the trimethylammonium group enhances the push–pull effect along the coumarin chromophore. Surprisingly, the molar extinction coefficient of **6** was considerably lower than that of the other compounds (e.g., ε = 33 for **6** vs. 48 mM^–1^ cm^–1^ for **5**), probably due to its aggregation in aqueous media. In addition, all COUPY derivatives showed emission in the far-red region with emission maxima ranging from 618 nm (**5**) to 625 nm (**6**), resulting in relatively high Stokes’ shifts (58–60 nm for **4–6**). As shown in Table 1, the fluorescent quantum yield of CLB-caged compounds **5** and **6** (Φ_F_ = 0.07–0.09) was lower than that of the 4-PBA photocage **4** and coumarin alcohol **12** (Φ_F_ = 0.23 and 0.26, respectively).

**Table 1:** Photophysical Properties of COUPY-Caged Compounds **4-6** and of Coumarin Alcohol **12**

	absorption		emission		
compound	λ_max_ (nm)^a^	ε(λ_max_) (mM^–1^ cm^–1^)^b^	λ_em_ (nm)^c^	Stokes’ shift (nm)^d^	Φ_F_ ^e^
**4**	563	51.0	620	60	0.23
**5**	563	48.1	618	58	0.07
**6**	568	32.8	625	60	0.09
**12**	555	29.0	612	59	0.26

a Wavelength of the absorption maximum.

b Molar absorption coefficient at λ_max_.

c Wavelength of the emission maximum upon excitation at 20 nm below λ_max_.

d Stokes’ shift.

e Fluorescence quantum yield.

**Figure 1:**
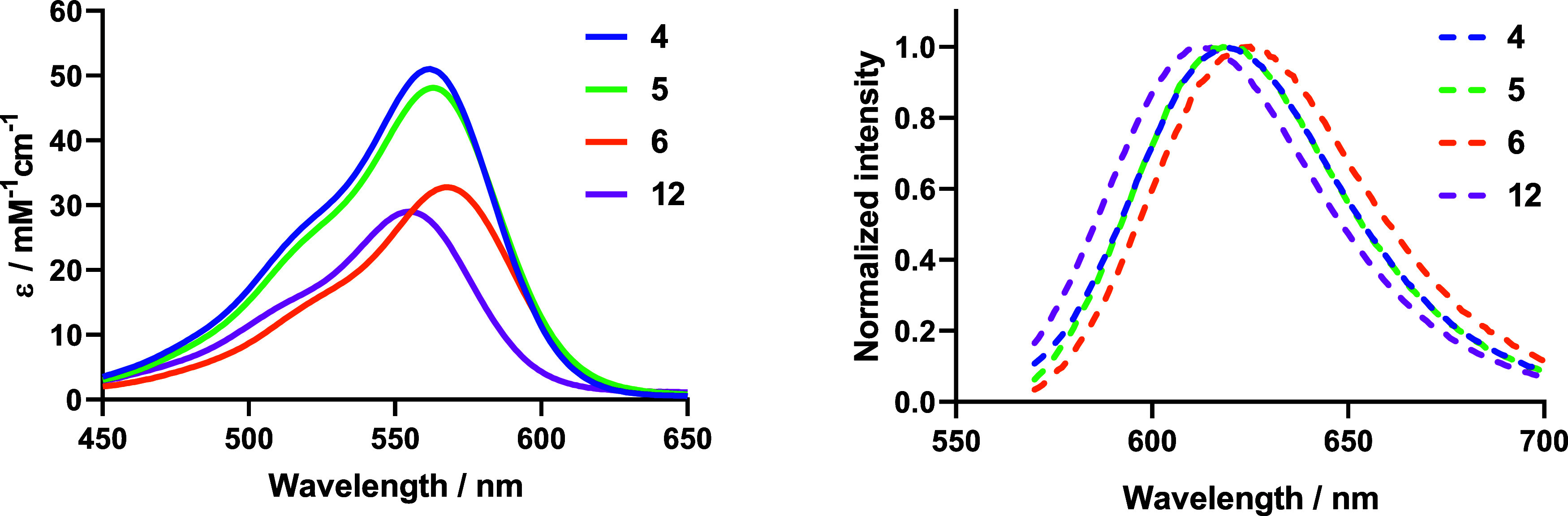
Absorption (left) and emission (right) spectra of COUPY photocages **4–6** and of coumarin alcohol **12**.

Central to PDT is the production of ROS by the PS under light irradiation.^66,67^ In addition to singlet oxygen (^1^O_2_), produced through an energy-transfer process (Type II PDT), Type I PDT involves electron-transfer reactions that result in the generation of a variety of cytotoxic ROS, including superoxide anion radical (^•^O_2_^–^).^68,69^ The ability of COUPY-caged compounds **4–6** to generate ROS upon green light irradiation (505 ± 35 nm, 100 mW cm^–2^) was studied by spectroscopic methods using two fluorogenic probes (Figure 2), namely singlet oxygen sensor green (SOSG) for ^1^O_2_ and dihydrorhodamine 123 (DHR123) for ^•^O_2_^–^. The ability of the parent COUPY PS **1** and the coumarin alcohol photoproduct **12** to photogenerate ROS was also investigated. As shown in Figures 2 and S7–S10, in all cases an increase of the fluorescence intensity of probes SOSG and DHR123 was observed upon irradiation with green light in the presence of the compounds, confirming their ability to photogenerate both singlet oxygen and superoxide, respectively. However, the efficiency of COUPY photocages **4–6** and coumarin alcohol **12** to photogenerate singlet oxygen was considerably lower than that of COUPY dye **1**. This result was further corroborated by the measurement of singlet oxygen quantum yields determined using 1,3-diphenylisobenzofuran (DPBF) as a ^1^O_2_ scavenger and methylene blue as a reference (Table S1 and Figures S11 and S12). Interestingly, the singlet oxygen quantum yield of 4-PBA-containing COUPY photocage was much higher than that of the CLB counterparts (e.g., Φ_Δ_ = 0.05 for **4** vs Φ_Δ_ < 0.01 for **5** and **6**), which indicates that the payload drug also influences the ability of the coumarin scaffold to photogenerate ROS. By contrast, the ability of COUPY-caged compounds **4–6** to photogenerate superoxide was much higher than that of the parent COUPY fluorophore **1**.

**Figure 2:**
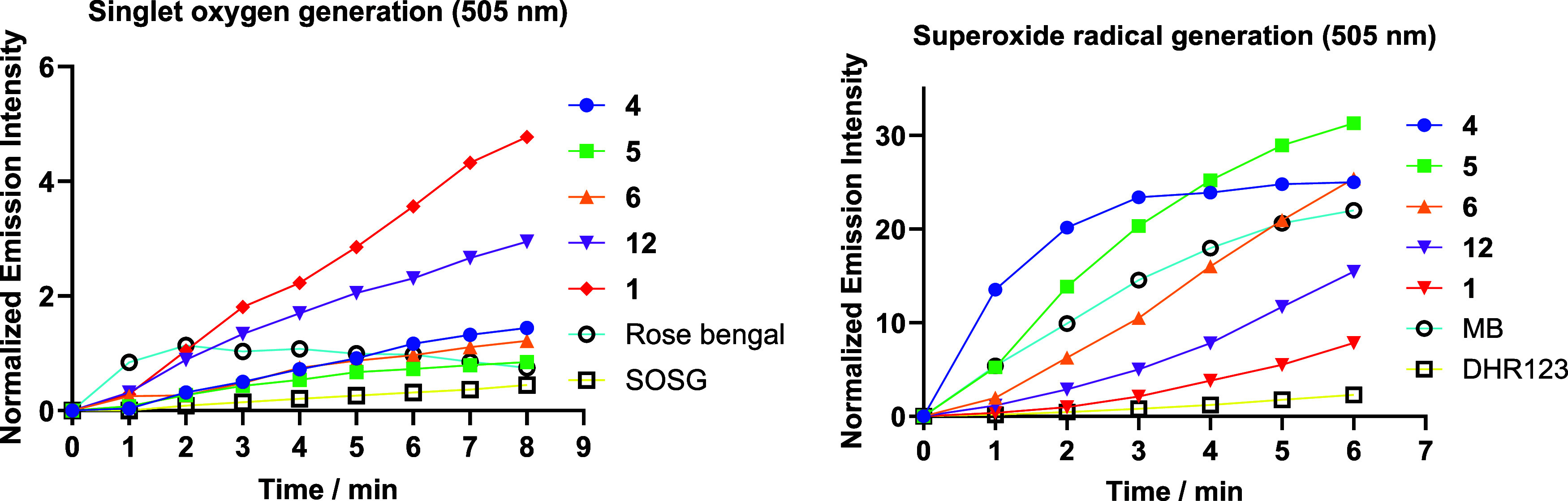
Photogeneration of singlet oxygen and superoxide by COUPY-caged compounds **4–6** and COUPY coumarins **1** and **12** studied using specific fluorogenic probes. Increase in fluorescence emission of SOSG (5 μM, left) and DHR123 (10 μM, right) upon irradiation of the compounds (10 μM) with green light (505 ± 35 nm, 100 mW cm^–2^) in PBS (2% DMSO).

As shown in Figures S8 and S10, further confirmation of the photogeneration of singlet oxygen and superoxide by COUPY-caged compounds **4–6** was obtained by using specific scavengers for each species (e.g., sodium azide for ^1^O_2_ and tiron for ^•^O_2_^–^). Taken together, ROS photogeneration studies indicate that modifications at position 4 of the COUPY coumarin backbone, either through hydroxylation (e.g., coumarin alcohol **12**) or attachment of a payload through an ester bond (e.g., COUPY photocages **4** and **5**), have a notable impact on the photochemical behavior of the compounds compared to the unmodified parent coumarin **1**, resulting in lower singlet oxygen production but increased generation of superoxide radical anions.

### Photolysis Studies of COUPY-Caged Compounds **4–6**

Photoactivation of COUPY-caged compounds **4–6** was evaluated in a mixture of PBS and ACN (8:2, v/v) at 37 °C. Prior to irradiation, the compounds were incubated for 2 h at 37 °C to assess their stability in the dark. No significant degradation was observed in either the PBS/ACN mixture or in Dulbecco’s Modified Eagle Medium (DMEM) supplemented with 10% FBS, confirming their suitability for further photorelease studies (Figures S13 and S14, respectively). Photolysis under visible light irradiation was then monitored by reversed-phase HPLC-MS following the progressive disappearance of the compounds over time (Figures S15–S17). As shown in Figure 3, the concentration of all compounds decreased gradually with increasing irradiation time.

**Figure 3:**
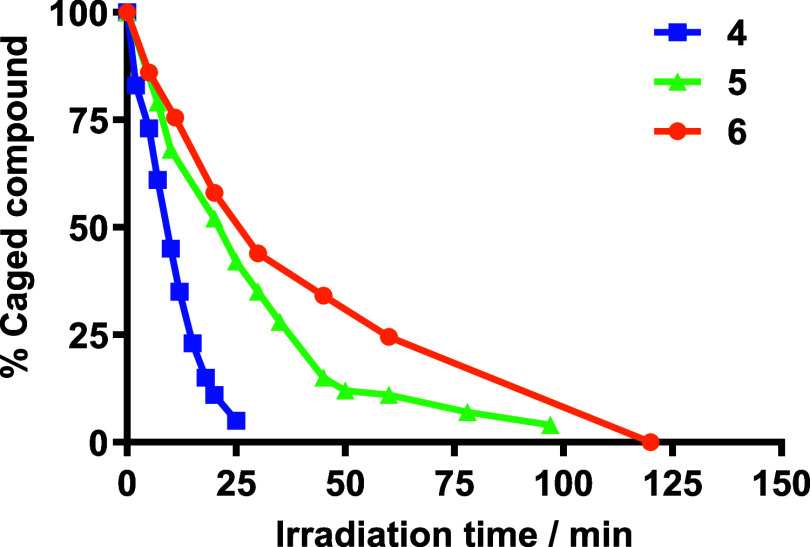
Plot of the temporal evolution of the amount of COUPY-caged compounds **4–6** upon irradiation with visible light (470–750 nm range, centered at 530 nm; 150 mW cm^–2^). Lines connecting the experimental data points are shown for visual guidance only. All of the experiments were performed in an 8:2 (v/v) mixture of PBS buffer and ACN at 37 °C.

Photolysis studies revealed that both antitumor drugs (CLB and 4-PBA) were efficiently photoreleased from their corresponding COUPY photocages, leading to a main coumarin alcohol photoproduct (**12** or **14**, Scheme 3). As previously found in other COUPY-caged model compounds containing a methyl group at the position adjacent to the photolabile bond, the secondary carbocation intermediate generated upon photoheterolysis of the ester bond^70^ also produced a minor vinyl coumarin photoproduct (**15** or **16**) through a β-elimination reaction (Scheme 3).^57,58^ Photolysis of compound **4** was significantly faster than that of compounds **5** and **6**, as 4-PBA was almost completely released within 25 min (*k*_u_ = 0.092 min^–1^). By contrast, photorelease of CLB from COUPY photocages **5–6** required more than 90 min to achieve a complete uncaging (*k*_u_ = 0.035 min^–1^ for **5** and *k*_u_ = 0.025 min^–1^ for **6**). These results are in good agreement with previous findings in other COUPY-caged model compounds such as COUPY photocages **2** and **3** containing benzoic acid and 2,4-dinitrophenol as payloads, thus confirming that the nature of the leaving group has a strong influence on the photoactivation process.^57,58^ To our delight, CLB was efficiently photoreleased without any structural modifications, demonstrating that the nitrogen mustard moiety remained stable under the irradiation conditions in aqueous medium up to 2 h (Figures S16–S17). The slower photolysis rate of compound **6** compared to **5** can be attributed to the stronger electron-withdrawing character of the 3-(*N*,*N*,*N*-trimethylammonium)­propyl moiety relative to the hexyl group. This electron deficiency may destabilize the carbocation component of the carbocation–carboxylate ion pair (Scheme 3), thereby reducing the rate constant of the photoheterolysis reaction.

**Scheme 3:**
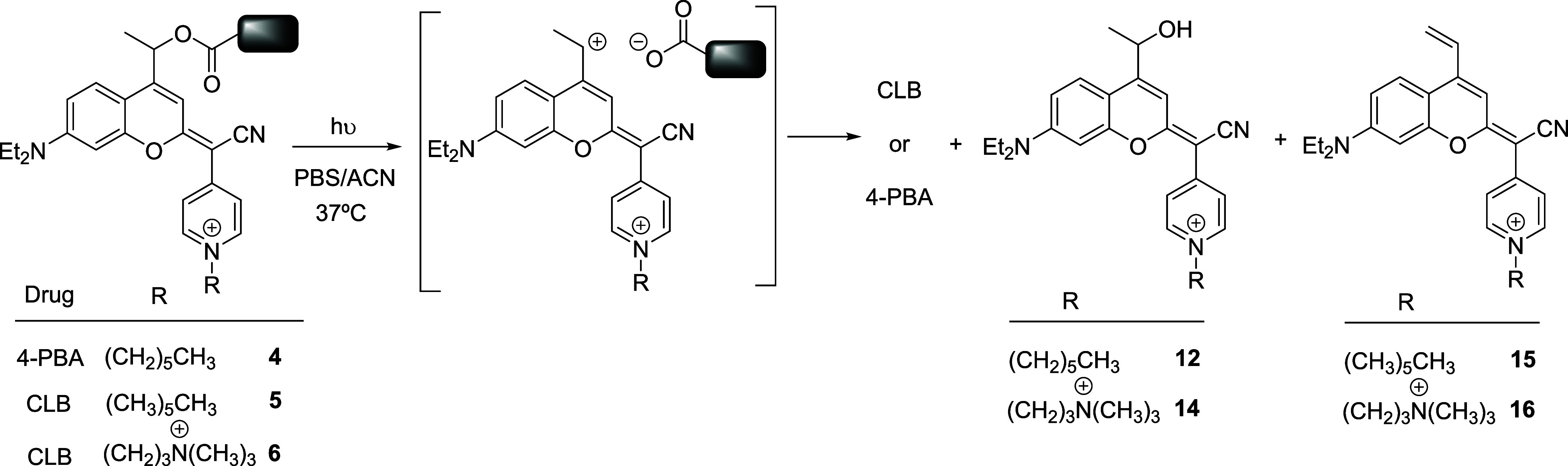
Mechanistic Interpretation of the Photolysis of COUPY-Caged Compounds **4-6** Under Visible Light Irradiation

The photolytic efficiency of the uncaging process was determined as the product of the molar absorption coefficient at the irradiation wavelength (ε­(λ_irr_)) and the uncaging quantum yield (Φ_Phot_) calculated from the rate of disappearance of COUPY photocages upon irradiation. As summarized in Table 2, compound **4** displayed a higher Φ_Phot_ than its CLB-containing counterparts, resulting in a significantly greater overall photolytic efficiency (Φ_Phot_ × ε*:* 1.22 for **4** compared to 0.38 for **5** and 0.19 for **6**).

**Table 2:** Photochemical Parameters for COUPY-Caged Compounds^a^

compound	*k*_u_ (min^–1^)^b^	Φ_Phot_ [× 10^–5^]^c^	ε(λ_irr_) [mM^–1^ cm^–1^]^d^	Φ_Phot_ × ε*(*λ_irr_) [M^–1^ cm^–1^]^e^
**4**	0.108	3.89	31.3	1.22
**5**	0.037	1.30	29.0	0.38
**6**	0.025	1.07	17.4	0.19

a Irradiation was performed with visible LED light (470–750 nm range, centered at 530 nm; 150 mW cm^–2^) in an 8:2 (v/v) mixture of PBS buffer and ACN.

b Uncaging first-order rate constant.

c Uncaging quantum yields were determined from the degradation of the compounds by actinometry.

d Molar absorption coefficient at the irradiation wavelength (530 nm).

e Photolytic efficiency at the irradiation wavelength.

Importantly, the CLB-caged derivatives underwent efficient photochemical cleavage, affording the corresponding products with nearly quantitative chemical yields upon completion of photolysis under visible-light irradiation (*ca* 92% for **5** after 97 min and *ca* 89% for **6** after 120 min), consistent with the complete consumption of the starting material, as confirmed by HPLC analysis (Figures S16–S18). In the case of compound **4**, the uncaging chemical yield could not be determined due to the partial precipitation of 4-PBA in the reaction medium, which prevented accurate quantification by HPLC-MS analysis. However, monitoring the photolysis process by ^1^H NMR in a mixture of CD_3_CN and D_2_O (2:8, v/v) enabled confirmation of the successful uncaging of 4-PBA (Figure 4).

**Figure 4:**
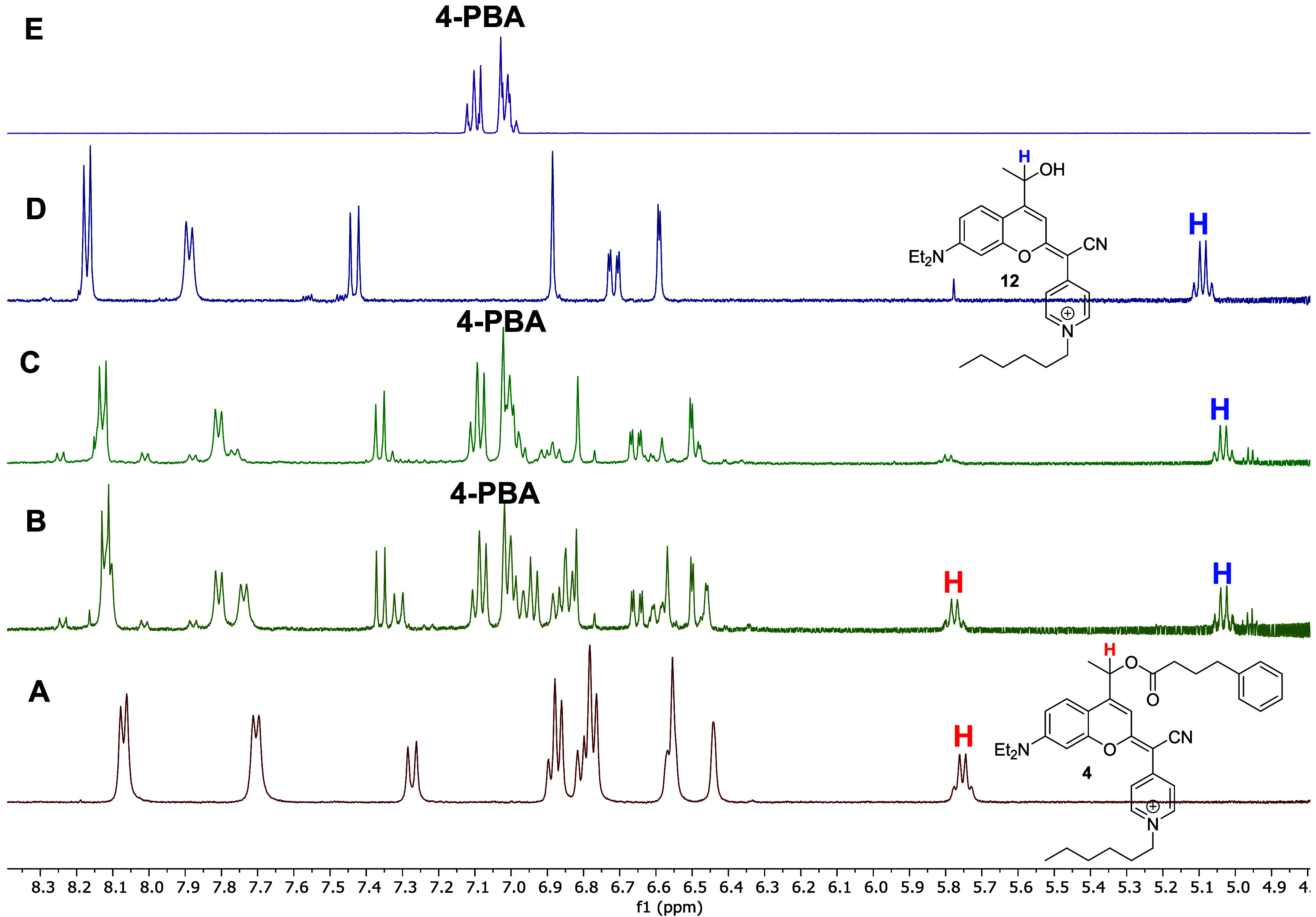
Expanded ^1^H NMR spectra (400 MHz, CD_3_CN/D_2_O, 2:8 v/v) of COUPY photocage **4** recorded (A) before irradiation and after (B) 2 h and (C) 4 h of visible-light irradiation at 37 °C. Spectra of the photoreleased products, coumarin alcohol **12** (D) and 4-phenylbutyric acid (4-PBA) (E), are shown for reference.

### Cellular Uptake Studies in Living HeLa Cells

Before assessing the (photo)­cytotoxicity of COUPY photocages **4–6**, we first examined their cellular uptake and subcellular localization in living HeLa cells using confocal microscopy, leveraging the fluorescent properties of the COUPY PPG. As shown in Figure 5, after a 30 min incubation at 37 °C (1 μM), fluorescence emission from compounds **4–6** was detected inside the cells upon excitation at 561 nm, confirming their rapid and efficient cellular uptake.

**Figure 5:**
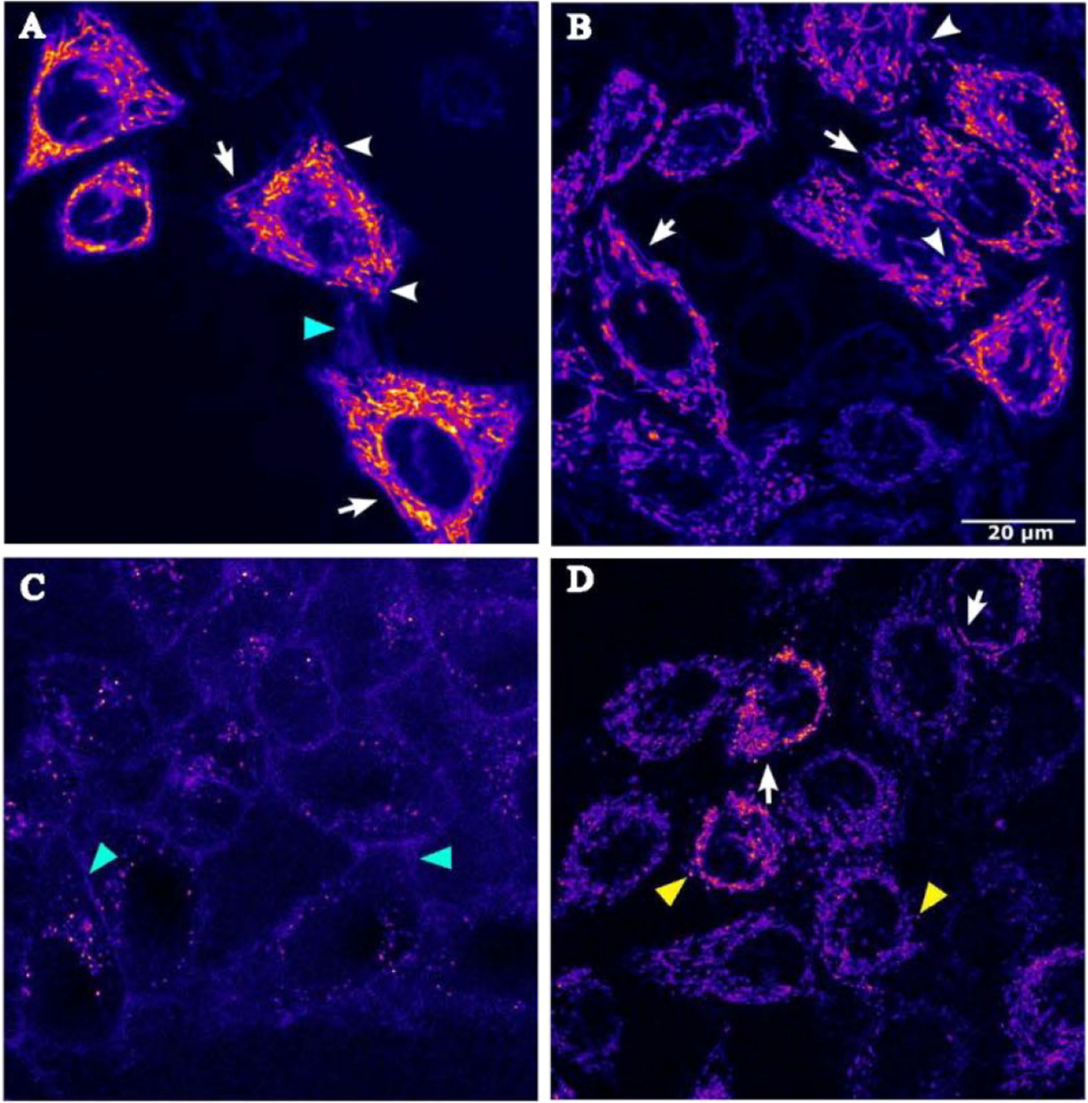
Cellular uptake of COUPY-caged compounds **4** (A), **5** (B), and **6** (C), and of the coumarin alcohol photoproduct **12** (D). Single confocal planes of HeLa cells incubated with the compounds (**4–6**, 1 μM, and **12**, 2 μM; 30 min, 37 °C). White arrows and arrowheads point out elongated and doughnut-shaped mitochondria, respectively. Cyan and yellow arrowheads point out extracellular membranes and intracellular vesicles, respectively. Lookup table (LUT): Fire. Scale bar: 20 μm.

For compounds **4** and **5**, the filamentous staining pattern was similar to that previously observed for the COUPY fluorophore **1**,^51^ COUPY photocage **3**,^57^ and the coumarin alcohol **12**,^57^ clearly indicating mitochondrial accumulation. In line with their fluorescence quantum yields, the emission intensity of COUPY-caged 4-PBA derivative **4** was slightly higher than that of the CLB-caged analogue **5**. Subsequent colocalization experiments with MitoTracker Green FM (MTG) confirmed the mitochondrial localization of both compounds (Figure S21), as evidenced by high Pearson’s correlation coefficients (*r* = 0.84 for **4** and *r* = 0.82 for **5**), indicating a strong correlation between the compounds’ signals and MitoTracker staining. As shown in Table S2, the Manders’ colocalization coefficients (e.g., M1 = 0.67; M2 = 0.80 for compound **4**) further confirmed this observation, indicating a robust and statistically significant association. Notably, colocalization studies with LysoTracker Green (LTG) revealed minimal lysosomal accumulation for both COUPY photocages **4** and **5**, as indicated by their low Pearson correlation coefficients (*r* = 0.19 and 0.21, respectively). These findings further support the conclusion that both compounds preferentially localize to mitochondria.

Interestingly, compound **6** exhibited a completely different staining pattern compared to its *N*-hexyl counterpart (Figure 5), primarily accumulating on the extracellular membrane and in intracellular vesicles. To determine the nature of the vesicles, we conducted colocalization experiments using the lysosome-specific fluorescent marker LysoTracker Green FM (LTG). We also used the fluorescent probe Wheat Germ Agglutinin Alexa Fluor 633 (WGA), which stains the extracellular membrane and endosomes formed by endocytosis (Figure S22). In both cases, moderate Pearson’s correlation coefficients were obtained (*r* = 0.44 for LTG, *r* = 0.59 for WGA), suggesting that the vesicles stained by compound **6** correspond primarily to lysosomes and endosomes. Thus, the incorporation of a 3-(*N*,*N*,*N*-trimethylammonium)­propyl moiety completely alters the intrinsic preference for mitochondria of monocationic *N*-alkyl COUPY dyes and PPGs, likely due to the inability of the resulting dication to permeate the phospholipid bilayer of the mitochondrial membrane.^71^ This result is consistent with theoretical and experimental studies that demonstrated that the subcellular distribution behavior between fluorophores containing an *N*-methyl-pyridinium unit and those with an *N*,*N*,*N*-trimethylammonium-alkyl-pyridinium group is different.^72,73^

### *In Vitro* (photo)­cytotoxicity Evaluation in HeLa Cells

Having demonstrated that COUPY-caged derivatives **4–6** can photorelease CLB and 4-PBA payloads upon visible light irradiation and that both the intact photocages and the coumarin alcohol photoproduct (compound **12**) can sensitize Type I and Type II ROS, we next focused on evaluating their (photo)­cytotoxicity toward HeLa cells, where efficient cellular uptake had previously been confirmed. Noteworthy, before conducting the cell-based assays, photolysis studies under cell-free conditions were performed using the same 96-well plate illuminator intended for cellular experiments. These tests confirmed the complete release of CLB and 4-PBA from the COUPY photocages at relatively low light fluences (e.g., < 3.6 J cm^–2^ for **4** and <7.2 J cm^–2^ for **5**; see Figures S23–S24), successfully reproducing the results obtained in cuvette-based experiments under visible-light irradiation. For (photo)­cytotoxicity assays, cells were incubated for 1 h with increasing concentrations of compounds **4–6**, **12** or the free antitumor drugs (CLB and 4-PBA). The latter served as controls to demonstrate the synergistic effect resulting from combining PDT and PACT in the new photocages, compared to single therapies. After refreshing the medium, cells were either kept in the dark or irradiated for 1 h with green-yellow LED light centered at 550 nm at a dose of 7.2 J cm^–2^. Following a 48-h period after treatment, cell viability was assessed using the MTT (3-[4,5-dimethylthiazol-2-yl]-2,5 2,5-diphenyl tetrazolium bromide) assay, and IC_50_ values, defined as the concentration required to inhibit cell growth by 50%, were determined from the corresponding dose–response curves (Figures S25–S27). The phototoxic index (PI), calculated as the ratio of dark to light IC_50_ values, was used to quantify the phototherapeutic efficiency of the compounds. This parameter accounts for the combined cytotoxic effect of both the anticancer drug (CLB or 4-PBA) photoreleased from the COUPY photocage and the photogenerated ROS (PACT + PDT).

As expected, all the coumarin-containing compounds displayed a much higher cytotoxicity toward cancer cells upon light irradiation than in the dark (Table 3). Nonetheless, COUPY-caged compounds **4** and **5** exhibited non-negligible dark cytotoxicity (e.g., IC_50_ = 3.76 μM for **5**), whereas the CLB-caged compound **6** was significantly less cytotoxic (IC_50_ = 40.58 μM). The differences in dark toxicity between the hexylated derivatives **4** and **5** compared to the dicationic compound **6** may be attributed to their distinct subcellular accumulation patterns. To our delight, IC_50_ values of compounds **4** and **5** under light conditions were found in the nanomolar range (IC_50_ = 0.027 μM for **4** and IC_50_ = 0.094 μM for **5**), leading to relatively high PI values (131 for **4** and 40 for **5**). In contrast, the IC_50_ of compound **6** under light conditions was much higher (10.74 μM), resulting in a much smaller PI value (3.8). The modest photocytotoxicity of the coumarin alcohol photoproduct **12** (IC_50_ = 0.65 μM) compared to compounds **4** and **5** suggests that the pronounced photocytotoxicity effects of these photocages cannot be solely attributed to ROS photogeneration by the coumarin moiety in the mitochondria. By contrast, CLB and 4-PBA were deemed noncytotoxic toward HeLa cells under both dark and light conditions (IC_50_ values >100 μM), probably due to poor cellular uptake. The low antiproliferative activity of CLB toward HeLa cells has been previously reported in the literature, even after longer incubation times.^74^

**Table 3:** (Photo)­cytotoxicity of Compounds **4-6**, **12**, and Antitumor Drugs (CLB and 4-PBA) Toward HeLa Cells Expressed as IC_50_ Values [μM]^a^ and Phototoxic Indexes (PI)^b^

compound	IC_50_ [μM] dark	IC_50_ [μM] light	PI
**4**	3.54 ± 0.24	0.027 ± 0.015	131
**5**	3.76 ± 0.13	0.094 ± 0.005	40
**6**	41 ± 10	11 ± 5	3.7
**12**	27.21 ± 2.69	0.7 ± 0.4	40
CLB	>100	>100	
4-PBA	>100	>100	

a Cells were treated for 2 h (1 h of incubation and 1 h of irradiation with green-yellow light (550 nm pulsed light (1 min ON, 1 min OFF), 7.2 J cm^–2^)) followed by 48 h recovery in drug-free medium. Control cells were left in the dark. IC_50_ values represent the mean of three independent biological replicates.

b Phototoxic index (PI) = IC_50_ (dark-nonirradiated cells)/IC_50_ (irradiated cells).

To get more insights into the potential applications of COUPY photocages in cancer phototherapy, the (photo)­cytotoxicity of compound **5** along with that of its photoproducts (**12** and CLB) was evaluated toward U87-MG human glioblastoma cells, due to their previously reported sensitivity to CLB^75^ and their clinical relevance, as glioblastoma represents one of the most aggressive and treatment-resistant brain tumors with a particularly poor prognosis. As shown in Table S3 and Figure S31, COUPY photocage **5** also exhibited high photocytotoxicity against glioblastoma cells upon green-yellow light irradiation, with IC_50_ values comparable to those observed in HeLa cells (e.g., IC_50_ = 0.094 μM in HeLa cells vs 0.12 μM in U87-MG cells). Again, the coumarin alcohol **12** exhibited modest phototoxic activity toward glioblastoma cells. Overall, these results indicate that the antitumor activity of CLB and 4-PBA can be considerably enhanced when incorporated into COUPY photocages, not only by facilitating efficient delivery to mitochondria but also thanks to the generation of cytotoxic ROS by the coumarin moiety.

## Conclusions

In summary, we have reported an efficient strategy to selectively deliver anticancer drugs into mitochondria using COUPY-based caging groups activatable with visible light. COUPY-caged compounds **4** and **5** exhibited several notable features for cancer phototherapy, including absorption in the visible region (λ_max_ ranging from 563 to 568 nm), large molar extinction coefficients (51.0 mM^–1^ cm^–1^ for **4** and 48.1 mM^–1^ cm^–1^ for **5**), dark stability in aqueous media, and fast photolysis kinetics, delivering the corresponding bioactive payload molecules without structural modification under visible light irradiation. Confocal microscopy studies revealed that the alkyl chain on the pyridine moiety played a crucial role in subcellular localization. While *N*-hexyl photocages **4** and **5** selectively accumulated in the mitochondria, the dicationic compound **6** localized to the cytosolic membrane and intracellular vesicles. Additionally, compounds **4** and **5** exhibited remarkable photocytotoxicity against HeLa cells under green-yellow light irradiation, with nanomolar IC_50_ values, leading to high PI values. Notably, CLB photocage **6** showed a much lower (photo)­cytotoxic activity, likely due to its extramitochondrial localization. More importantly, while coumarin alcohol **12** exhibited modest phototoxicity against HeLa cells through mitochondrial ROS generation, its lower activity compared to photocages **4** and **5**, underscores the enhanced efficacy achieved through the synergistic combination of PDT and PACT mechanisms in the latter. Overall, this study showcases the versatility of COUPY-based photocages for developing novel mitochondria-targeted anticancer phototherapeutic agents, expanding the toolbox of PACT/PDT dual agents.

## Experimental Section

### Materials and Methods

Common chemicals and solvents (HPLC grade or reagent grade quality) were purchased from commercial sources and used without further purification. A hot plate magnetic stirrer, together with an aluminum reaction block of the appropriate size, was used as the heating source in all reactions requiring heat. Aluminum plates coated with a 0.2 mm thick layer of silica gel 60 F254 were used for thin-layer chromatography analyses (TLC), whereas column chromatography purification was carried out using silica gel 60 (230–400 mesh). Reversed-phase high-performance liquid chromatography (HPLC) analyses were carried out on a Jupiter Proteo C12 column (150 × 4.6 mm, 90 Å 4 μm, flow rate: 1 mL/min, column 1) or on a XSelectCSH Phenyl-Hexyl column (100 × 4.6 mm, 130 Å 3.5 μm, flow rate: 1 mL/min, column 2) using linear gradients of 0.1% formic acid in H_2_O (A) and 0.1% formic acid in ACN (B). All final compounds were >95% pure by this method. NMR spectra were recorded at 25 °C in a 400 MHz spectrometer using the deuterated solvent as an internal deuterium lock. The residual protic signal of chloroform, DMSO, MeOH, or CH_3_CN was used as a reference in ^1^H and ^13^C­{^1^H} NMR spectra recorded in CDCl_3_, DMSO-*d*_6_, CD_3_OD, and CD_3_CN, respectively. Chemical shifts are reported in part per million (ppm) in the δ scale, coupling constants in Hz and multiplicity as follows: s (singlet), d (doublet), t (triplet), q (quartet), qt (quintuplet), m (multiplet), dd (doublet of doublets), dq (doublet of quartets), br (broad signal), etc. Electrospray ionization (ESI) mass spectra were recorded on an instrument equipped with a single quadrupole detector coupled to an HPLC, and high-resolution (HR) ESI-MS on an LC/MS-TOF instrument.

### Synthesis of COUPY Scaffolds (8–13)

#### Compound **8**

A mixture of coumarin **7** (1.60 g, 6.12 mmol), phenylbutyric acid (1.50 g, 9.15 mmol), EDC hydrochloride (1.75 g, 9.15 mmol) and DMAP (1.12 g, 9.15 mmol) was cooled at 0 °C under an argon atmosphere and then dissolved in DCM (100 mL). The mixture was stirred at 0 °C for 15 min and then 18 h at room temperature. Then, the solution was washed with saturated NH_4_Cl (2 × 100 mL), 5% aqueous NaHCO_3_ (1 × 100 mL, 2 × 50 mL), and deionized water (100 mL). The organic layer was dried over anhydrous MgSO_4_, filtered and evaporated under reduced pressure. The product was isolated by silica column chromatography (silica gel, 50–100% DCM in hexanes, 1–3% MeOH in DCM) to give 2 g of a yellow solid (yield: 80%). TLC: Rf (DCM) 0.6. ^1^H NMR (400 MHz, CDCl_3_) δ (ppm): 7.38 (1H, d, *J* = 7.28 Hz), 7.27 (3H, m), 7.19 (3H, m), 6.58 (1H, dd, *J* = 7.1, 1.9 Hz), 6.51 (1H, d, *J* = 1.9 Hz), 6.12 (1H, s), 6.07 (1H, q, *J* = 5.6 Hz), 3.41 (4H, q, *J* = 5.3 Hz), 2.67 (2H, t, *J* = 5.6 Hz), 2.42 (2H, m), 1.99 (2H, qt, *J* = 5.8 Hz), 1.57 (3H, d, *J* = 5.6 Hz), and 1.20 (6H, t, *J* = 5.3 Hz). ^13^C­{^1^H} NMR (101 MHz, CDCl_3_): δ (ppm) 172.5, 162.4, 156.7, 155.7, 150.7, 141.3, 128.6, 128.6, 126.2, 124.9, 108.8, 105.8, 105.0, 98.1, 67.3, 44.9, 35.2, 33.8, 26.5, 21.1, 12.6. HRMS (ESI-TOF) *m*/*z* [M + H]^+^ 408.2169 calcd for C_25_H_30_NO_4_^+^, found 408.2169; analytical HPLC (10 to 100% B over 15 min, column 1) R_t_ = 11.10 min.

#### Compound **9**

Lawesson’s reagent (1.54 g, 3.81 mmol) was added to a solution of coumarin **8** (1.94 g, 4.75 mmol) in toluene (60 mL) under an argon atmosphere. The mixture was stirred at 105 °C in the dark overnight. After removal of the solvent under reduced pressure, the product was isolated by column chromatography (silica gel, 0–30% hexanes in DCM) to give 1.67 g of an orange solid (yield: 83%). TLC: R_f_ (DCM) 0.78. ^1^H NMR (400 MHz, CDCl_3_) δ (ppm): 7.45 (1H, d, *J* = 8.8 Hz), 7.29 (2H, m), 7.19 (3H, m), 7.06 (1H, s), 6.67 (2H, m), 6.07 (1H, q, *J* = 6.5 Hz), 3.42 (4H, q, *J* = 7.2 Hz), 2.66 (2H, t, *J* = 7.2 Hz), 2.42 (2H, t, *J* = 8 Hz), 1.99 (2H, qt, *J* = 7.2 Hz), 1.54 (3H, d, *J* = 6.5 Hz), 1.22 (6H, t, *J* = 7.2 Hz). ^13^C­{^1^H} NMR (101 MHz, CDCl_3_): δ (ppm) 197.6, 172.5, 159.6, 151.0, 148.1, 141.3, 128.6, 128.6, 126.2, 125.0, 119.3, 110.5, 108.1, 97.8, 67.0, 53.6, 45.1, 35.2, 33.7, 26.5, 21.0, 12.6. HRMS (ESI-TOF) *m*/*z* [M + H]^+^ 424.1941 calcd for C_25_H_30_NO_3_S^+^, found 424.1936; analytical HPLC (30 to 100% B over 15 min, column 1) R_t_ = 8.87 min.

#### Compound **10**

4-Pyridylacetonitrile hydrochloride (125 mg, 0.807 mmol) and NaH (60% dispersion in mineral oil, 64.6 mg, 2.69 mmol) were dissolved in anhydrous ACN (30 mL) under an argon atmosphere. After stirring for 15 min at room temperature, a solution of thiocoumarin **9** (171 mg, 0.404 mmol) in anhydrous ACN (15 mL) was added dropwise under Ar, and the reaction mixture was stirred at room temperature for 2 h and protected from light. Then, AgNO_3_ (166 mg, 1.00 mmol) was added and the mixture was stirred at room temperature for 2 h. The crude was evaporated under reduced pressure and purified by column chromatography (silica gel, 50–100% DCM in hexanes, and then 0.1–0.8% MeOH in DCM) to give 121 mg of an orange solid (79% yield). TLC: R_f_ (DCM) 0.25. ^1^H NMR (400 MHz, DMSO-*d*_6_) δ (ppm): (major rotamer, *E*) 8.58 (2H, dd, *J* = 4 Hz, 1.6 Hz), 7.73 (2H, dd, *J* = 4 Hz, 1.6 Hz), 7.58 (1H, dd, *J* = 8 Hz, 2.4 Hz), 7.27 (2H, m), 7.19 (3H, m), 6.74 (2H, m), 6.71 (1H, s), 6.06 (1H, q, *J* = 6.6 Hz), 3.49 (4H, q, *J* = 7.1 Hz), 2.62 (2H, t, *J* = 7.6 Hz), 2.42 (2H, br t, *J* = 6.9 Hz), 1.90 (2H, m), 1.55 (3H, d, *J* = 6.6 Hz), 1.15 (6H, t, *J* = 7.1 Hz). ^13^C­{^1^H} NMR (101 MHz, CDCl_3_) δ (ppm): (major rotamer, *E*) 172.5, 163.1, 162.9, 154.8, 150.6, 149.7, 146.7, 141.2, 140.9, 128.5, 128.4, 126.0, 124.8, 120.9, 119.4, 109.3, 108.7, 106.6, 97.3, 83.4, 67.4, 44.7, 35.1, 33.7, 26.5, 20.8, 12.5. HRMS (ESI-TOF) *m*/*z* [M + H]^+^ 508.2595 calcd for C_32_H_34_N_3_O_3_^+^, found 508.2599; analytical HPLC (30 to 100% B over 15 min, column 1) R_t_ = 8.49 min. Rotamers ratio *E/Z* 90:10.

#### Compound **11**

To a solution of coumarin **10** (242 mg, 0.477 mmol) in a 2:1 (v/v) mixture of ACN and H_2_O (40 mL), an aqueous solution of sodium hydroxide 0.25 M (5.72 mL, 1.43 mmol) was added and the reaction mixture was stirred overnight at room temperature. After removal of the solvent under pressure, the product was purified by column chromatography (silica gel, 50–100% DCM in hexanes, and then 0.25–5% MeOH in DCM) to give 139 mg of an orange solid (80% yield). TLC: Rf (5% MeOH in DCM) 0.38; ^1^H NMR (400 MHz, DMSO-*d*_6_) δ (ppm): (major rotamer, *E*) 8.55 (2H, m), 7.72 (2H, m), 7.52 (1H, d, *J* = 9.2 Hz), 6.96 (1H, s), 6.71 (1H, s), 6.70 (1H, m), 5.61 (1H, d, *J* = 4.0 Hz), 5.06 (1H, m), 3.46 (4H, q, *J* = 7.2 Hz), 1.40 (3H, d, *J* = 6.4 Hz), 1.14 (6H, t, *J* = 7.2 Hz). ^13^C­{^1^H} NMR (101 MHz, DMSO-*d*_6_) δ (ppm): (major rotamer, *E*) 163.7, 154.2, 153.6, 150.4, 140.1, 125.4, 120.1, 119.4, 109.5, 106.4, 106.3, 96.9, 80.5, 63.9, 43.8, 24.2, 12.4; HRMS (ESI-TOF) *m*/*z* [M + H]^+^ calcd for C_22_H_23_N_3_O_2_ 362.1869, found 362.1872; analytical HPLC (10 to 100% B over 15 min, column 1) R_t_ = 7.92 min. Rotamers ratio *E/Z* 92:8.

#### Compound **13**

A mixture of coumarin **11** (80.0 mg, 0.22 mmol), CLB (100 mg, 0.33 mmol), EDC·hydrochloride (63.6 mg, 0.33 mmol) and DMAP (40.6 mg, 0.33 mmol) was cooled at 0 °C under an argon atmosphere and then dissolved in DCM (15 mL). The mixture was stirred at 0 °C for 15 min and then 18 h at room temperature. After removal of the solvent under reduced pressure, the product was purified by column chromatography (silica gel, 0–0.8% MeOH in DCM) to give 122 mg of an orange solid (85% yield). TLC: R_f_ (5% MeOH in DCM)= 0.47. TLC: R_f_ (5% MeOH in DCM) 0.47. ^1^H NMR (400 MHz, DMSO-*d*_6_) δ (ppm): (major rotamer, *E*) 8.59 (2H, dd, *J* = 4 Hz, 1.6 Hz), 7.73 (2H, dd, *J* = 4 Hz, 1.6 Hz), 7.58 (1H, dd, *J* = 8 Hz, 3.5 Hz), 7.02 (2H, m), 6.75 (2H, m), 6.71 (1H, s), 6.65 (2H, m), 6.06 (1H, q, *J* = 6.1 Hz), 3.68 (8H, m), 3.49 (4H, q, *J* = 7.1 Hz), 2.49 (2H, m), 2.41 (2H, br t, *J* = 7.3 Hz), 1.85 (2H, m), 1.55 (3H, d, *J* = 6.1 Hz), 1.15 (6H, t, *J* = 7.1 Hz). ^13^C­{^1^H} NMR (101 MHz, DMSO-*d*_6_) δ (ppm): (major rotamer, *E*) 172.3, 163.3, 154.8, 151.2, 150.5, 148.5, 145.0, 140.2, 129.8, 129.8, 125.9, 120.8, 119.5, 112.3, 110.2, 107.0, 106.1, 97.6, 82.1, 68.0, 52.7, 44.5, 44.4, 41.6, 33.7, 33.5, 27.0, 21.2, 21.0, 12.9, 12.8. HRMS (ESI-TOF) *m*/*z* [M + H]^+^ 647.2550 calcd for C_36_H_41_N_4_O_3_^+^, found 647.2541; analytical HPLC (30 to 100% B over 15 min, column 1) R_t_ = 10.29 min. Rotamers ratio *E/Z* 92:8.

### Synthesis of COUPY-Caged Compounds (**4–6**)

#### Compound **4**

To a solution of coumarin **10** (50 mg, 0.099 mmol) in anhydrous ACN (3 mL), 1-bromohexane (1.04 mL, 7.39 mmol) was added under an Ar atmosphere and the reaction mixture was stirred overnight at 60 °C. After removal of the solvent under reduced pressure, the product was purified by column chromatography (silica gel, 0.2–1.8% MeOH in DCM) to give 44 mg of a pink solid (67% yield). TLC: R_f_ (5% MeOH in DCM) 0.51. ^1^H NMR (400 MHz, CDCl_3_) δ (ppm): 8.75 (2H, d, *J* = 7.1 Hz), 8.31 (2H, d, *J* = 7.1 Hz), 7.45 (1H, d, *J* = 9.3 Hz), 7.39 (1H, d, *J* = 2.4 Hz), 7.22 (2H, m), 7.12 (3H, m), 6.96 (1H, s), 6.69 (1H, dd, *J* = 12, 2.5 Hz), 6.07 (1H, q, *J* = 6.7 Hz), 4.38 (2H, t, *J* = 7.5 Hz), 3.60 (4H, m), 2.61 (2H, t, *J* = 7.5 Hz), 2.40 (2H, t, *J* = 7.5 Hz), 1.93 (4H, m), 1.55 (3H, d, *J* = 6.7 Hz), 1.24 (12H, m), 0.82 (3H, m). ^13^C­{^1^H} NMR (101 MHz, CDCl_3_) δ (ppm): 172.4, 167.8, 156.0, 154.0, 152.8, 149.6, 142.5, 141.1, 128.5, 128.4, 126.0, 125.1, 121.9, 118.3, 112.0, 107.3, 106.4, 98.7, 80.5, 67.4, 60.0, 45.5, 35.1, 33.6, 31.4, 31.1, 26.4, 25.8, 22.4, 21.3, 13.9, 12.7. HRMS (ESI-TOF) *m*/*z* [M]^+^ 592.3534 calcd for C_38_H_46_N_3_O_3_, found 592.3521; analytical HPLC (30 to 100% B over 15 min, column 1) R_t_ = 11.70 min.

#### Compound **5**

To a solution of **12** (62 mg, 0.129 mmol) in DCM at 0 °C (10 mL), a solution of DMAP (23.7 mg, 0.194 mmol), EDC·hydrochloride (37.2 mg, 0.194 mmol) and chlorambucil (59.0 mg, 0.194 mmol) in DCM (12 mL) was added. The reaction mixture was stirred for 15 min at 0 °C and then it was left stirring at room temperature overnight. After removal of the solvent under reduced pressure, the product was purified by column chromatography (silica gel, 60–100% DCM in hexane and then, 0–5.5% MeOH in DCM) to afford 76.2 mg of a pink solid (77%). TLC: R_f_ (10% MeOH in DCM) 0.40. ^1^H NMR (400 MHz, CDCl_3_) δ (ppm): 8.79 (2H, d, *J* = 7.1 Hz), 8.40 (2H, d, *J* = 7.1 Hz), 7.50 (1H, d, *J* = 9.0 Hz), 7.47 (1H, d, *J* = 2.8 Hz), 7.07 (2H, m), 7.03 (1H, s), 6.75 (1H, dd, *J* = 9.0, 2.6 Hz), 6.62 (2H, m), 6.15 (1H, q, *J* = 6.8 Hz), 4.49 (2H, m), 3.65 (12H, m), 2.58 (2H, br t, *J* = 8.4 Hz), 2.45 (2H, br t, *J* = 8.4 Hz), 1.97 (4H, m), 1.61 (3H, d, *J* = 6.8 Hz), 1.28 (12H, m), 0.88 (3H, m). ^13^C­{^1^H} NMR (101 MHz, CD_3_OD) δ (ppm): 174.0, 169.1, 157.2, 156.2, 153.9, 151.1, 146.0, 143.8, 131.3, 130.7, 129.9, 129.2, 127.2, 126.3, 122.5, 119.0, 113.5, 113.4, 108.6, 107.3, 98.0, 81.5, 69.1, 61.0, 54.5, 45.9, 41.7, 35.0, 34.4, 32.3, 32.2, 27.9, 26.9, 23.5, 21.6, 14.3, 12.8. HRMS (ESI-TOF) *m*/*z* [M]^+^ 731.3489 calcd for C_42_H_53_Cl_2_N_4_O_3_, found 731.3487; analytical HPLC (60 to 100% B over 15 min, column 1) R_t_ = 10.97 min.

#### Compound **6**

To a solution of compound **13** (40 mg, 0.062 mmol) in anhydrous CH_3_CN (5 mL) under an Ar atmosphere, potassium iodide (20 mg, 0.123 mmol) and (3-bromopropyl)­trimethylammonium bromide (242 mg, 0.926 mmol) were sequentially added, and the resulting mixture was heated to 82 °C and stirred for 72 h. After cooling to room temperature, Amberlite IRA-410 (Cl^–^ form, 5 g) was added, and the mixture was stirred overnight at 60 °C. Then, the solvent was evaporated under reduced pressure to give the crude product, which was purified by column chromatography (silica gel, 0–50% MeOH in DCM, then 0–5% 0.1 M aq. KNO_3_ in CH_3_CN) to afford the title compound (22 mg, 43%) as a pink solid. TLC: R_f_ (10% 0.1 M aq. KNO_3_ in CH_3_CN) 0.42. ^1^H NMR (400 MHz, CD_3_OD) δ 8.61 (d, *J* = 7.3 Hz, 2H), 8.28 (d, *J* = 7.4 Hz, 2H), 7.76 (d, *J* = 9.3 Hz, 1H), 7.07 – 7.02 (m, 4H), 7.00 (dd, *J* = 9.3, 2.6 Hz, 1H), 6.65 (d, *J* = 8.8 Hz, 2H), 6.19 (q, *J* = 6.7 Hz, 1H), 4.52 (t, *J* = 7.5 Hz, 2H), 3.70 – 3.57 (m, 12H), 3.55 – 3.46 (m, 2H), 3.19 (s, 9H), 2.63 – 2.53 (m, 4H), 2.46 (t, *J* = 7.5 Hz, 2H), 2.05 – 1.86 (m, 2H), 1.63 (d, *J* = 6.7 Hz, 3H), 1.31 – 1.23 (m, 6H). ^13^C­{^1^H} NMR (101 MHz, CD_3_OD) δ 174.0, 169.3, 157.3, 156.5, 154.1, 151.6, 146.0, 143.8, 131.3, 130.7, 127.2, 122.6, 119.0, 113.5, 108.7, 107.2, 98.1, 81.5, 69.1, 64.0, 57.3, 54.5, 53.8, 46.0, 41.7, 35.0, 34.4, 30.8, 27.9, 25.8, 21.6, 12.8. HRMS (ESI-TOF) *m*/*z* [M/2]^2+^ 373.6835 calcd for C_42_H_55_Cl_2_N_5_O_3_^2+^, found 373.6842; analytical HPLC (30 to 100% B over 15 min, column 1) Rt = 6.12 min.

### Photophysical Characterization of the Compounds

Absorption spectra were recorded in a Jasco V-730 spectrophotometer at room temperature. Molar absorption coefficients (ε) were determined by direct application of the Beer–Lambert law, using solutions of the compounds in a 8:2 (v/v) mixture of PBS buffer and ACN with concentrations about 10^–6^ M. Emission spectra were registered in a Photon Technology International (PTI) fluorimeter. Fluorescence quantum yields (Φ_F_) were measured by the comparative method using cresyl violet in ethanol (CV; Φ_F;ref_ = 0.54 ± 0.03) as a reference.^76–78^ Then, optically matched solutions of the compounds and CV were excited and the fluorescence spectra were recorded. The absorbance of sample and reference solutions was set below 0.1 at the excitation wavelength (540 nm) and Φ_F_ values were calculated using the following eq 1

1 $$\Phi_{F;Sample}=\frac{{Area}_{Sample}}{{Area}_{Ref}}x{\frac{{Abs}_{Ref}}{{Abs}_{Sample}}x\left(\frac{\eta_{Sample}}{\eta_{Ref}}\right)}^{2}x\Phi_{F;ref}$$

where Area_Sample_ and Area_Ref_ are the integrated fluorescence for the sample and the reference, Abs_Sample_ and Abs_Ref_ are the absorbance registered at 540 nm and η_Sample_ and η_Ref_ are the refractive indexes of the sample and reference solutions, respectively.

### ROS Determination

The generation of specific ROS was assessed using Singlet Oxygen Sensor Green (SOSG) and dihydrorhodamine 123 (DHR123) probes for singlet oxygen and superoxide, respectively. All measurements were carried using a Hellma 1.5 mL PTFE-stoppered fluorescence quartz cuvette (4 clear windows) with a 1 cm path length. SOSG (5 μM) or DHR123 (10 μM) were added to a solution of the corresponding compound (10 μM) in PBS containing 2% DMSO. Positive control experiments were carried out using Rose Bengal for SOSG or Methylene blue (MB) for DHR123 as references. Negative control experiments were carried out using sodium azide-saturated PBS as a singlet oxygen scavenger or sodium 4,5-dihydroxybenzene-1,3-disulfonate (tiron) saturated PBS as a superoxide anion radical scavenger.

Singlet oxygen quantum (Φ_Δ_) yields of the compounds were determined in air-saturated DCM (bubbled for 15 min) using 1,3-diphenylisobenzofuran (DPBF) as a chemical trap upon green light irradiation using a high-power LED source (505 ± 35 nm, 100 mW cm^–2^).^57^ The initial absorbance of DPBF in DCM was adjusted to ∼1.0 (50 μM), after which the COUPY compounds were added to the cuvette and their absorbance was adjusted to 0.06 at the light irradiation wavelength (505 nm). Then, the decrease in the absorbance of DPBF at 411 nm was monitored after irradiation at 505 nm. The linear relationship between the variation in DPBF absorbance at 411 nm (A_0_-A_t_) and irradiation time was plotted. Singlet oxygen quantum yields were calculated by the following eq 2

2 $$\Phi_{\Delta ,\mathrm{sample}}=\Phi_{\Delta ,\mathrm{ref}}\frac{m_{s}}{m_{r}}\frac{\left(1-10^{A\lambda r}\right)}{\left(1-10^{A\lambda s}\right)}$$

where Φ_Δ_, _ref_ is the reference singlet oxygen quantum yield of methylene blue (MB) (Φ_Δ_, _ref_ = 0.57 in aerated DCM),^79^ *m* are the slopes and Aλs and Aλr are the absorbance of the compounds and of the reference (MB) at 505 nm.

### Irradiation Experiments

Photolysis studies were performed at 37 °C in a custom-built irradiation setup from Microbeam, which includes a cuvette, thermostated cuvette holder, and a mounted high-power LED of a wide range (470–750 nm range, centered at 530 nm)^57^ light. In a typical experiment, the cuvette containing 1.5 mL of a solution of the caged compound (20 μM) and 4-*N,N’*-dimethylaminopyridine (internal standard, 20 μM) in a 8:2 (v/v) mixture of PBS buffer and ACN was placed in front of the light source and irradiated for the indicated times while constantly stirred. At each time point, samples were taken and analyzed by reversed-phase HPLC-ESI MS with column 2 by using linear gradients of 0.1% formic acid in H_2_O (A) and 0.1% formic acid in ACN (B).

### Uncaging Quantum Yield Determination

The uncaging quantum yields (Φ_Phot_) of COUPY photocages **4**-**6** were determined by actinometry using [1,2-bis­(2,4-dimethyl-5-phenyl-3-thienyl)­perfluorocyclopentene] (DAE), which acts as a visible-light actinometer. Photon fluxes calculated from DAE actinometry, together with the uncaging photolysis parameters obtained for each photocage, enabled the determination of Φ_Phot_ values (see Supporting Information).

### Confocal Microscopy Studies

#### Cell Culture and Treatments

HeLa cells were maintained in DMEM (Dullbecco Modified Eagle Medium) containing high glucose (4.5 g/L) and supplemented with 10% fetal bovine serum (FBS) and 50 U/mL penicillin-streptomycin. For cellular uptake experiments and posterior observation under the microscope, cells were seeded on glass-bottom dishes (P35G-1.5–14-C, Mattek). Twenty-four hours after cell seeding, cells were incubated for 30 min at 37 °C with the compounds (**4–6**, 1 μM and **12**, 2 μM) in DMEM supplemented with 10% FBS. Then, the cells were washed three times with DPBS (Dulbecco’s phosphate-buffered saline, pH 7.0–7.3) to remove the excess of the COUPY compounds and kept in low glucose DMEM without phenol red for fluorescence imaging.

For colocalization experiments with Mitotracker Green FM, HeLa cells were treated with compounds **4–6** (1 μM) or **12** (2 μM) and MitoTracker Green FM (0.1 μM) for 30 min at 37 °C in nonsupplemented DMEM. After removal of the medium and washing three times with DPBS, cells were kept in low-glucose DMEM without phenol red for fluorescence imaging.

For colocalization experiments with LysoTracker Green FM (LTG) and Wheat Germ Agglutinin (WGA), HeLa cells were incubated with compound **6** (1 μM) for 30 min at 37 °C in nonsupplemented DMEM. WGA and LTG were added during the last 20 and 5 min, respectively. After incubation, cells were washed three times with DPBS and maintained in low-glucose DMEM without phenol red for fluorescence imaging.

#### Fluorescence Imaging

All microscopy observations were performed using a Zeiss LSM 880 confocal microscope equipped with a 405 nm laser diode, an argon-ion laser, a 561 nm laser, and a 633 nm laser. The microscope was also equipped with a Heating Insert P S (Pecon) and a 5% CO_2_-providing system. Cells were observed at 37 °C using a 63 × 1.4 oil immersion objective. Compounds **4**-**6** and **12** were excited using the 561 nm laser and detected from 570 to 670 nm. MTG and LTG were observed using the 488 nm laser line of the argon-ion laser, whereas the 633 nm laser was used for observing WGA. Image processing and analysis were performed using Fiji.^80^

#### Co-Localizations Coefficients

The mitotracker, lysotracker, WGA, and compound channels were processed by median filtering (radius = 1), Gaussian filtering (sigma = 1), and background subtraction (rolling ball radius = 30). Then images were segmented by applying the Li threshold^81^ and the resulting binary images were used to mask the original images. Colocalization coefficients were measured using the JaCoP plugin^82^ on the different stacks of images (*n* = 5) with each stack containing 25 cells on average.

### (Photo)­cytotoxicity Evaluation

HeLa and U87-MG cells were seeded in 96-well flat-bottom plates at a density of 5000 cells/well in DMEM supplemented with 10% (v/v) FBS and 1% (v/v) penicillin-streptomycin (100 U/mL penicillin, 100 μg/mL streptomycin) (total volume: 95 μL). After incubation for 24 h, 5 μL of a solution of compounds **4**-**6** or **12** were added at the final concentrations in the range of 0 to 200 μM to give a final volume of 100 μL per well. Stock solutions prepared in DMSO were serially diluted in supplemented DMEM (10% FBS, 1% P/S) to obtain the desired final concentrations, maintaining a constant DMSO concentration of 0.5%. For (photo)­cytotoxicity studies, the light-based treatment regime was performed as follows: 1 h incubation (5% CO_2_, 37 °C) with the compound in the dark, followed by 1 h incubation under either dark or irradiation conditions by placing 96-well LED array plates (LEDA Teleopto) below the samples. Illumination was carried out for 1 h at 550 nm with a 4 mW/cm^2^ pulsed light (1 min ON, 1 min OFF). Potency was measured using a Thorlabs PM100D power energy meter connected with a standard photodiode power sensor (S120VC). Then, the drug-containing media was removed, and 100 μL of fresh medium was added. Cells were then incubated for 48 h. Cell viability was determined with an MTT assay by adding 10 μL of a 12 mM 3-(4,5-dimethylthiazol-2-yl)-2,5-diphenyltetrazolium bromide solution in PBS, followed by 4 h of incubation. The medium was aspirated, and the samples were solubilized with 200 μL of DMSO, allowing absorbance to be recorded using a Tecan Spark 20 M Multimode Microplate reader. Data from dose–response sigmoidal curves were processed with GraphPad software to calculate IC_50_ values (*n* = 3 replicates).

## Supplementary Material





## Notes

Additional figures and material from stability studies, ROS photogeneration studies, irradiation experiments, and fluorescence imaging, (photo)­cytotoxicity evaluation, copies of ^1^H and ^13^C­{^1^H} NMR and HRMS spectra of the synthesized compounds (PDF)
Molecular Formula Strings (CSV)

## Abbreviations Used

ACN: acetonitrile
CLB: chlorambucil
CV: cresyl violet
DCM: dichloromethane
DMEM: Dulbecco modified Eagle medium
DMF: *N,N*-dimethylformamide, DMSO, dimethyl sulfoxide
ESI-TOF: electrospray ionization time-of-fly
FBS: fetal bovine serum
HeLa: cervical carcinoma cell line
HPLC-MS: high-performance liquid chromatography–mass spectrometry
HRMS: high-resolution mass spectrometry
LED: light-emitting diode
LW: Lawesson’s reagent
NIR: near-infrared
NMR: nuclear magnetic resonance
NOESY: nuclear overhauser enhancement spectroscopy
PACT: photochemically activated chemotherapy
4-PBA: phenylbutyric acid
PBS: phosphate-buffered saline
PDT: photodynamic therapy
PPG: photocleavable protecting group
PI: phototoxic/phototherapeutic index
PS: photosensitizer
ROS: reactive oxygen species
RT: room temperature
TLC: thin-layer chromatography
U87-MG: human glioblastoma cell line
UV: ultraviolet
