# Supplementary material for: Exploring the Phototherapeutic Applications of Mitochondria-Targeted COUPY Photocages of Antitumor Drugs
Source: J Med Chem. 2025 Apr 28;68(9):9741–54. doi: 10.1021/acs.jmedchem.5c00550 (PMC12164338; doi:10.1021/acs.jmedchem.5c00550)
Supplement: Supplementary file 1 [file jm5c00550_si_001.pdf]

SUPPORTING INFORMATION

Exploring the phototherapeutic applications of mitochondria-targeted COUPY photocages of antitumor drugs

Marta López-Corrales,[1] Eduardo Izquierdo-García,[1] Manel Bosch,[2] Tapas Das,[3] Amadeu Llebaria,[4] Laia Josa-Culleré,[4] Vicente Marchán[1,*]

[1] Departament de Química Inorgànica i Orgànica, Secció de Química Orgànica, Universitat de Barcelona (UB), and Institut de Biomedicina de la Universitat de Barcelona (IBUB), Martí i Franquès 1-11, E-08028 Barcelona, Spain. Email: vmarchan@ub.edu

[2] Unitat de Microscòpia Òptica Avançada, Centres Científics i Tecnològics (CCiTUB), Universitat de Barcelona (UB), Av. Diagonal 643, E- 08028 Barcelona, Spain.

[3] Department of Chemistry, National Institute of Technology Jamshedpur, Jamshedpur, Jharkhand, 831014, India

[4] MCS, Department of Biological Chemistry, Institute for Advanced Chemistry of Catalonia (IQAC-CSIC), Jordi Girona 18-26, 08034, Barcelona, Spain

Table of contents

1.-Reversed-phase HPLC analysis of COUPY-caged compounds (**4-6**) S3

2.- 2D NMR characterization of coumarin scaffolds (**10** and **13**) S4

3.- 2D NMR characterization of COUPY-caged compounds (**4-6**) S6

4.- ROS photogeneration studies

4.1.- Evaluation of singlet oxygen generation using SOSG S9

4.2.- Evaluation of superoxide anion radical generation using DHR123 S11

4.2.- Determination of singlet oxygen quantum yield S13

5.- Irradiation experiments S15

6.- Determination of uncaging quantum yield (Φ_{Phot}) S20

7.- Confocal microscopy studies S22

8.- (Photo)cytotoxicity studies S24

9.- ^{1}H and ^{13}C spectra and HR ESI-MS of the compounds S30

10.- References S46

1.- Reversed-phase HPLC analysis of COUPY-caged compounds (4-6).

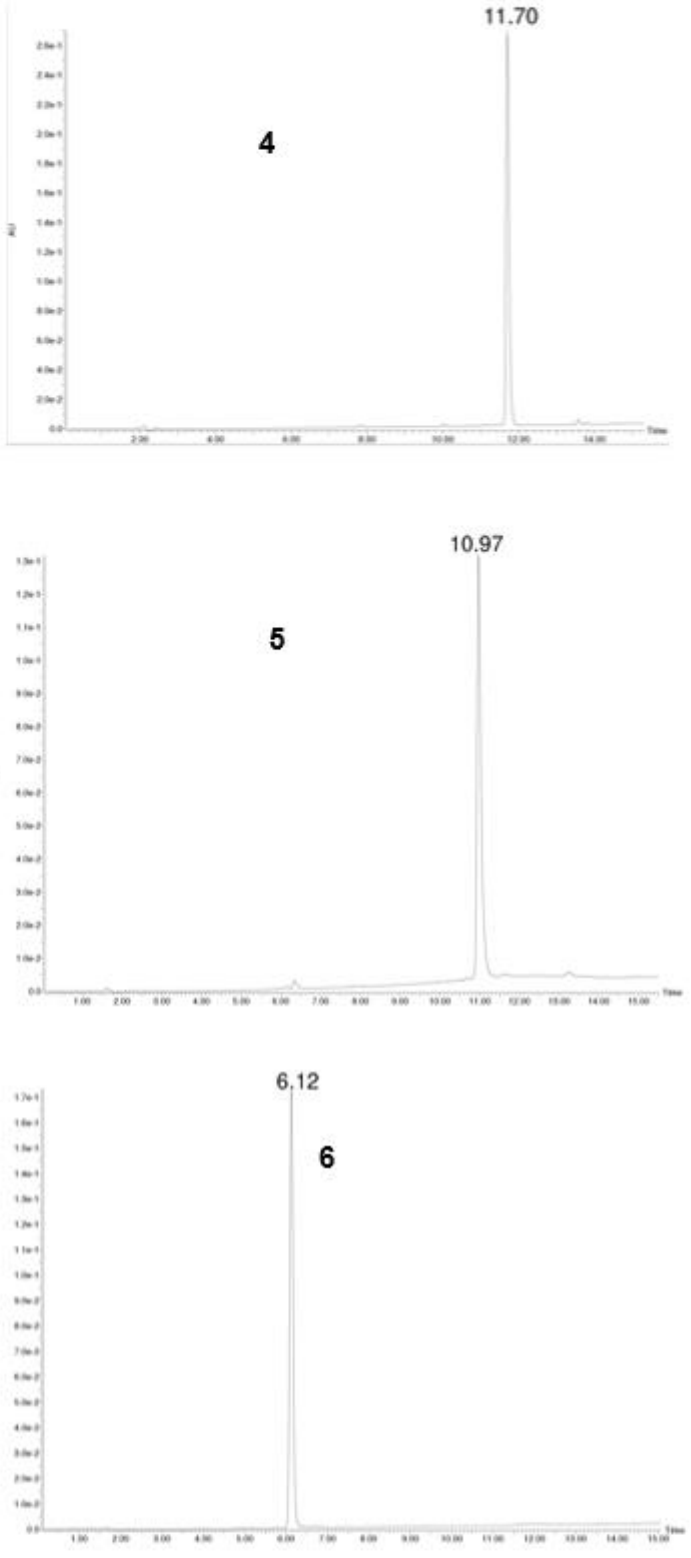

Figure S1. Reversed-phase HPLC traces at 260 nm of COUPY-caged compounds (**4-6**) using column 1.

2.- 2D NMR characterization of COUPY scaffolds (10 and 13)

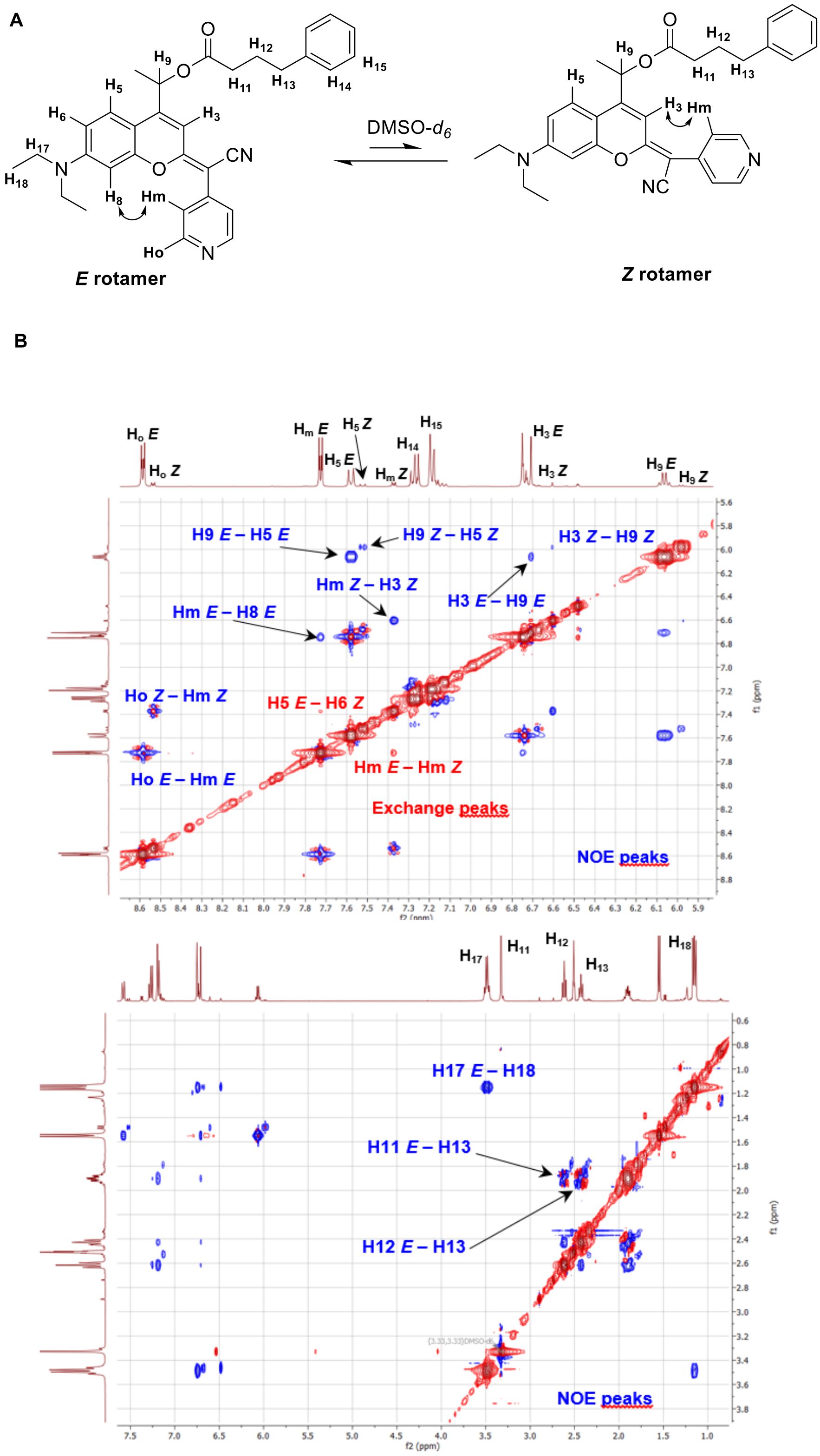

Figure S2. A) Structure of *E* and *Z* rotamers of coumarin **10** with some diagnostic NOE cross-peaks indicated. B) Expansions of the NOESY spectrum (DMSO-d_6, 298 K) showing some characteristic NOE cross-peaks and exchange cross-peaks between rotamer resonances of the same sign as the diagonal of **10**

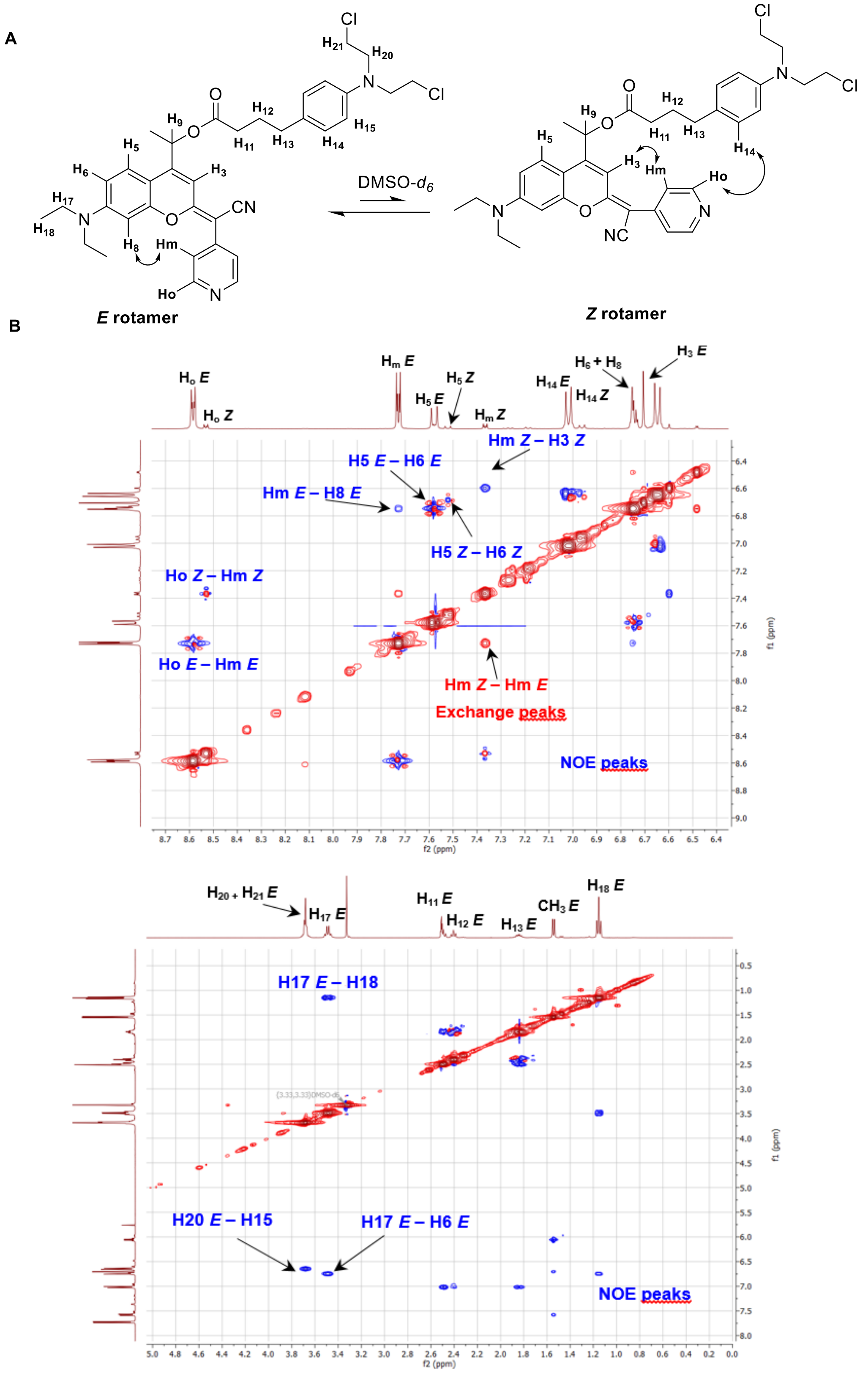

Figure S3. A) Structure of *E* and *Z* rotamers of coumarin **13** with some diagnostic NOE cross-peaks indicated. B) Expansions of the NOESY spectrum (DMSO-d_6, 298 K) showing some characteristic NOE cross-peaks and exchange cross-peaks between rotamer resonances of the same sign as the diagonal of **13**.

3.- 2D NMR characterization of COUPY-caged compounds (4-6)

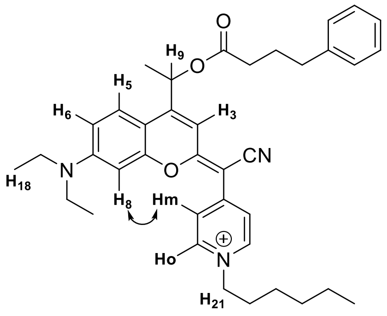

E rotamer

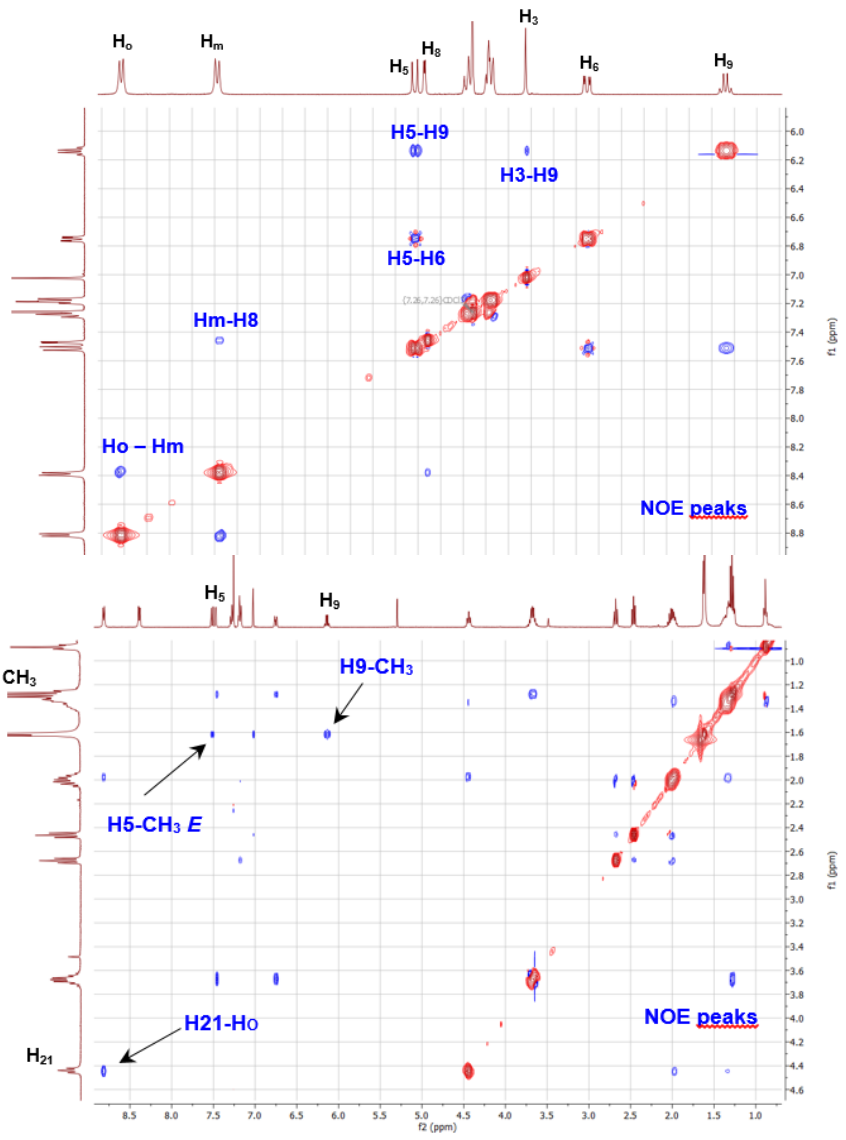

Figure S4. Structure of *E* rotamer of compound **4** with some diagnostic NOE cross-peaks indicated, and expansions of the NOESY spectrum showing some characteristic NOE cross-peaks in $CDCl_3$ at 298 K.

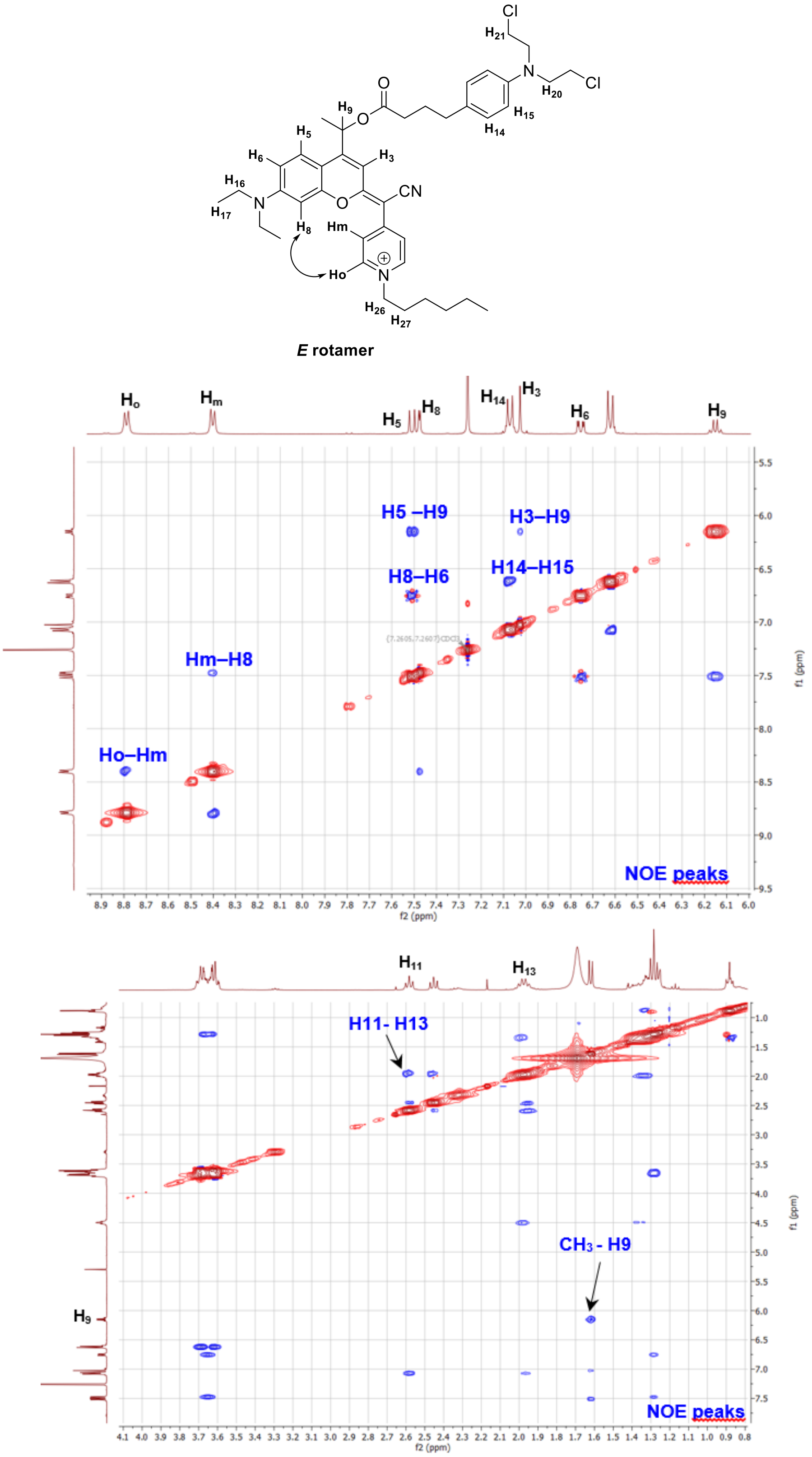

Figure S5. Structure of *E* rotamer of compound **5** with some diagnostic NOE cross-peaks indicated, and expansions of the NOESY spectrum showing some characteristic NOE cross-peaks in $CDCl_3$ at 298 K.

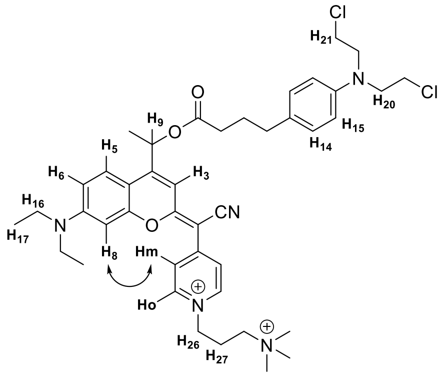

E rotamer

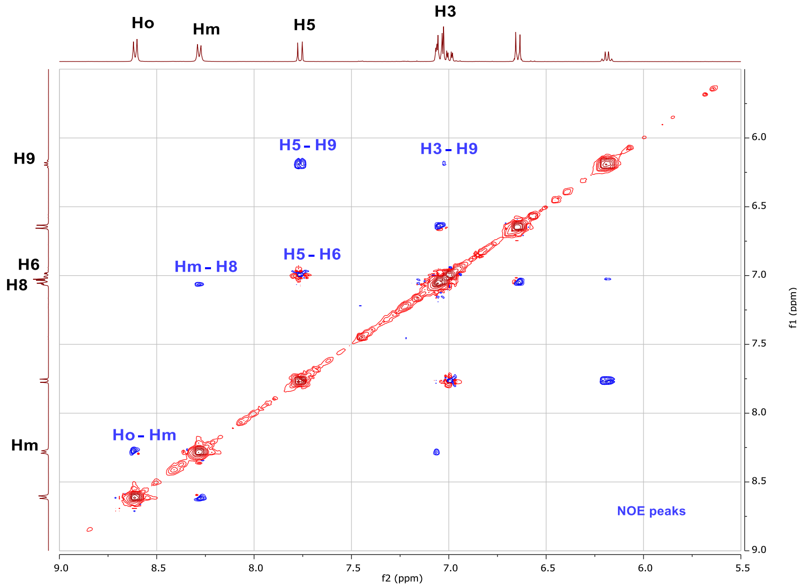

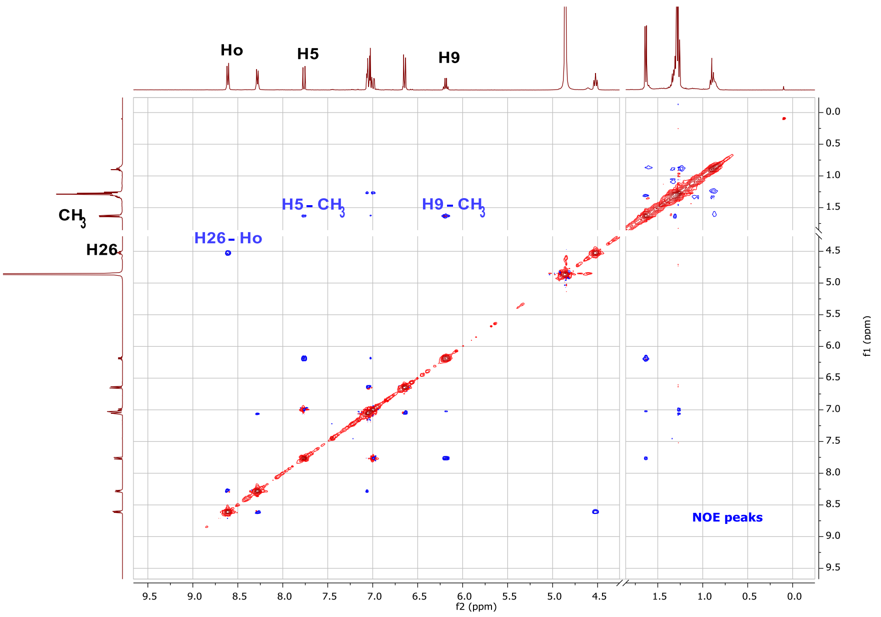

Figure S6. Structure of *E* rotamer of compound **6** with some diagnostic NOE cross-peaks indicated, and expansions of the NOESY spectrum showing some characteristic NOE cross-peaks in CD_3OD at 298 K.

4- ROS photogeneration studies

4.1.- Evaluation of singlet oxygen generation using SOSG

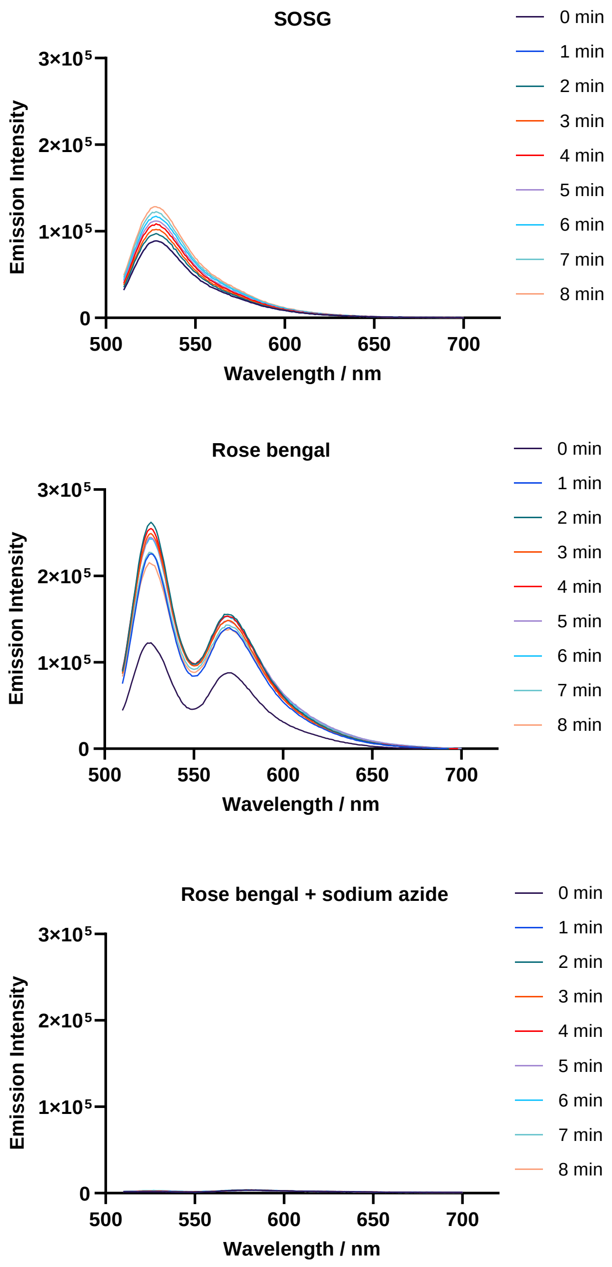

Figure S7. Photogeneration of singlet oxygen by Rose Bengal. Emission spectra of SOSG upon irradiation with green light (505±35 nm, 100 mW cm^{-2}) in PBS (2 % DMSO) (top). Increase of the fluorescence emission of SOSG upon irradiation of Rose Bengal at 505 nm in PBS (2 % DMSO) alone (middle) or in the presence of sodium azide-saturated PBS (2 % DMSO) as a singlet oxygen scavenger (bottom).

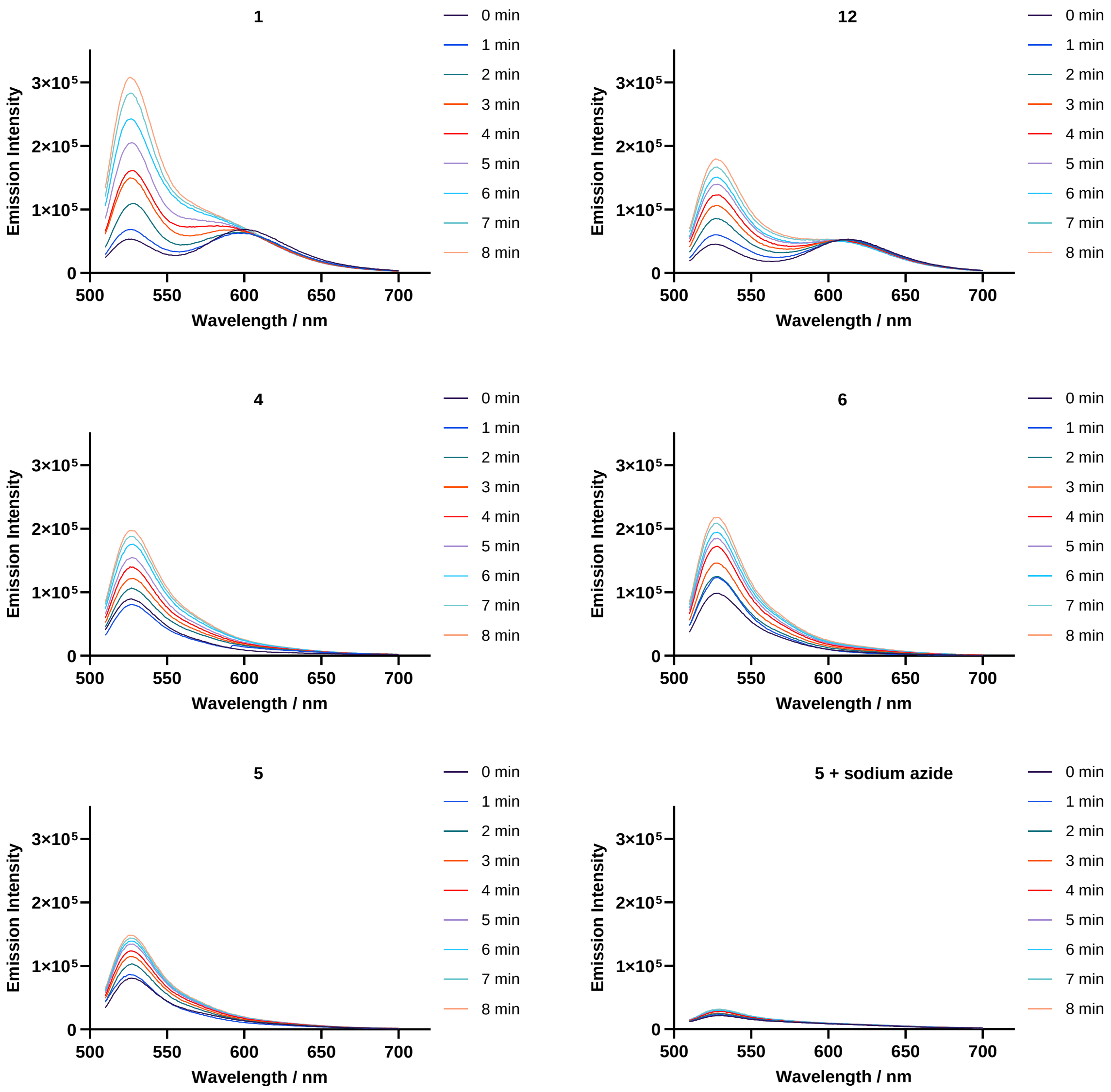

Figure S8. Photogeneration of singlet oxygen by COUPY-caged compounds **4-6**, coumarin alcohol **12** and COUPY fluorophore **1**. Increase of the fluorescence emission of SOSG upon irradiation of the compounds and SOSG with green light (505±35 nm, 100 mW cm^{-2}) in PBS (2 % DMSO). Representative negative control experiment using sodium azide-saturated PBS (2 % DMSO) as a singlet oxygen scavenger with compound **5**.

4.2.- Evaluation of superoxide anion radical generation using DHR123.

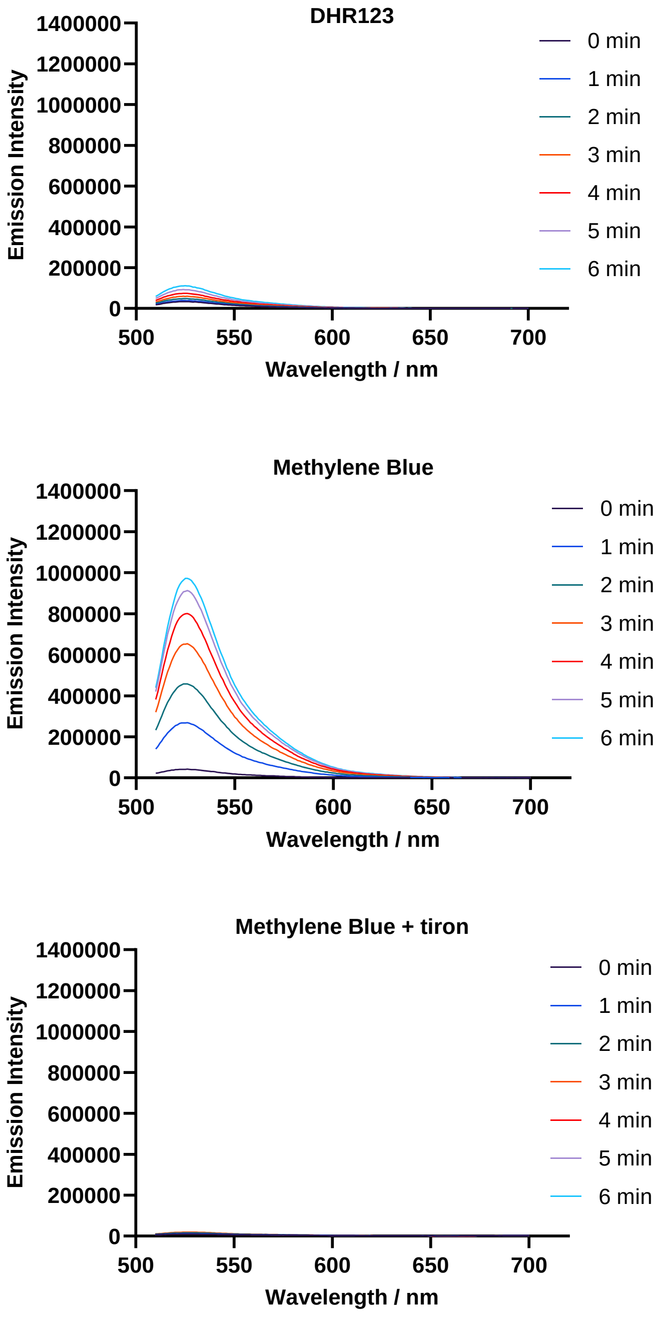

Figure S9. Photogeneration of superoxide anion radical by Methylene blue. Emission spectra of DHR123 upon irradiation with green light (505±35 nm, 100 mW cm^{-2}) in PBS (2 % DMSO) (top). Increase of the fluorescence emission of DHR123 upon irradiation of Methylene Blue at 505 nm in PBS (2 % DMSO) alone (middle) or in the presence of tiron saturated-PBS (2 % DMSO) as a superoxide anion scavenger (bottom).

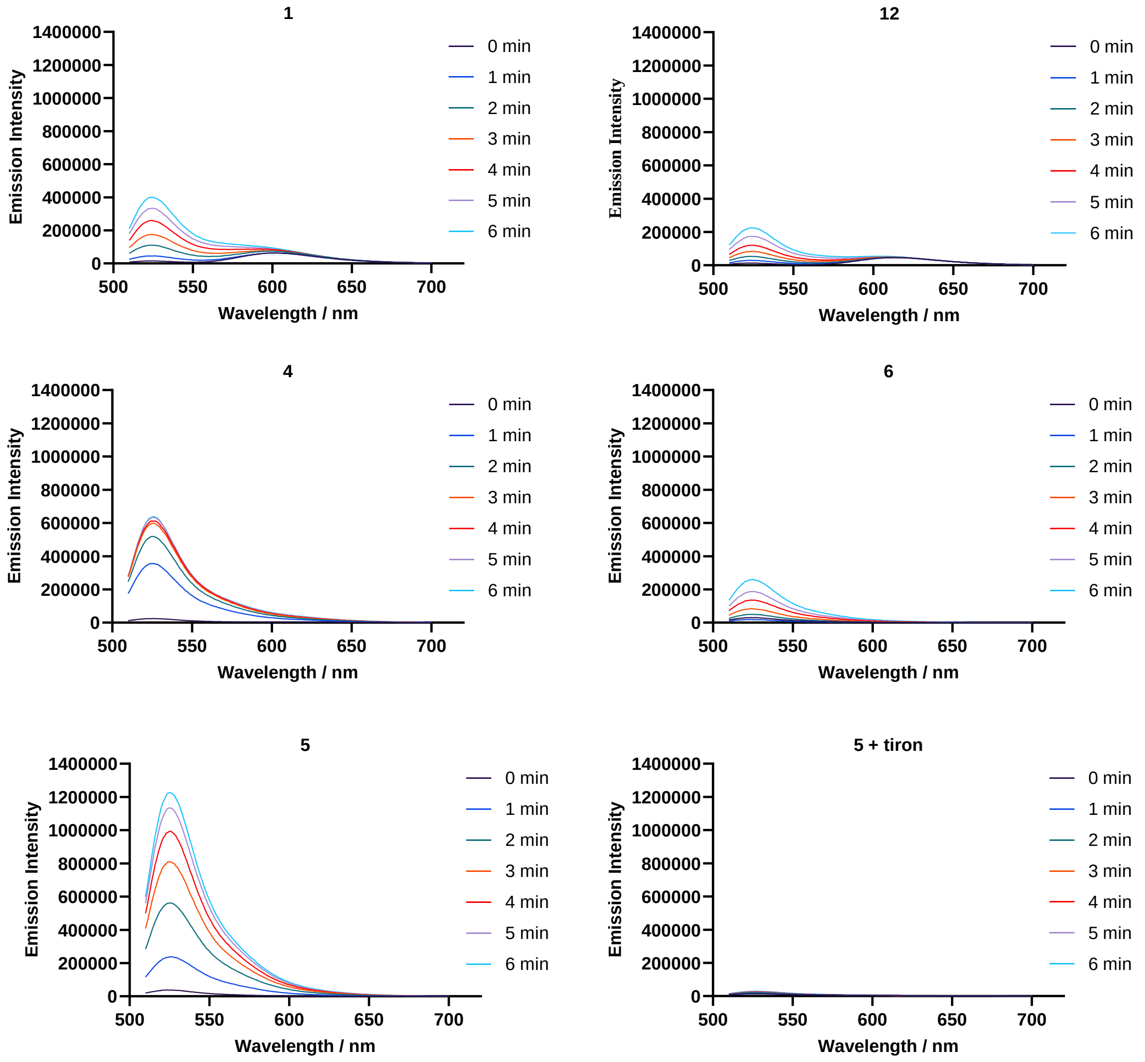

Figure S10. Photogeneration of superoxide by COUPY-caged compounds **4-6**, coumarin alcohol **12** and COUPY fluorophore **1**. Increase of the fluorescence emission of DHR123 upon irradiation of the compounds and DHR123 with green light (505±35 nm, 100 mW cm^{-2}) in PBS (2 % DMSO). Representative negative control experiment using tiron-saturated PBS (2 % DMSO) as a superoxide scavenger with compound **5**.

4.3.- Determination of singlet oxygen quantum yield

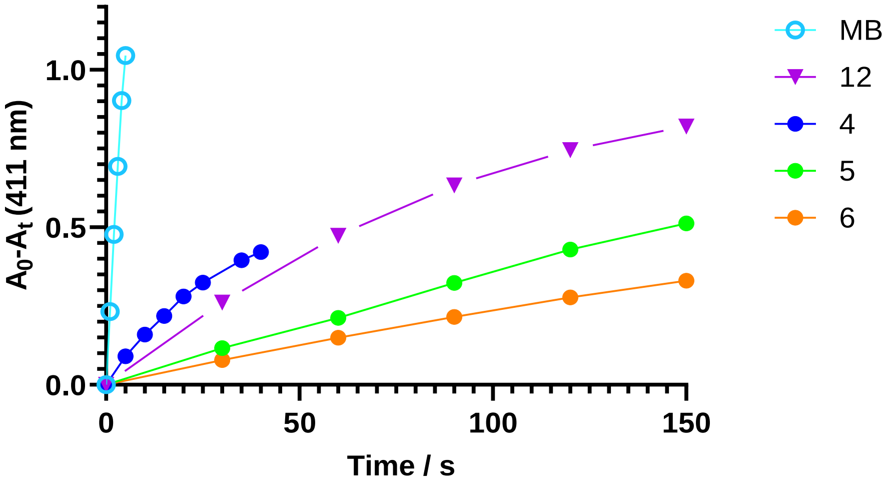

Figure S11. Plot of the changes in the absorbance (A_0-A_t) of DPBF at 411 nm against irradiation time with green light (505±35 nm, 100 mW cm^{-2}) in the presence of the standard sensitizer methylene blue (MB) or compounds **12** and **4-6** in aerated DCM.

Table S1. Singlet oxygen quantum yields (Φ_Δ) in air-saturated DCM (bubbled for 15 min) using DPBF.

compound	**Φ_Δ 505 nm**
**1**	0.11
**12**	0.017
**4**	0.052
**5**	< 0.01
**6**	< 0.01

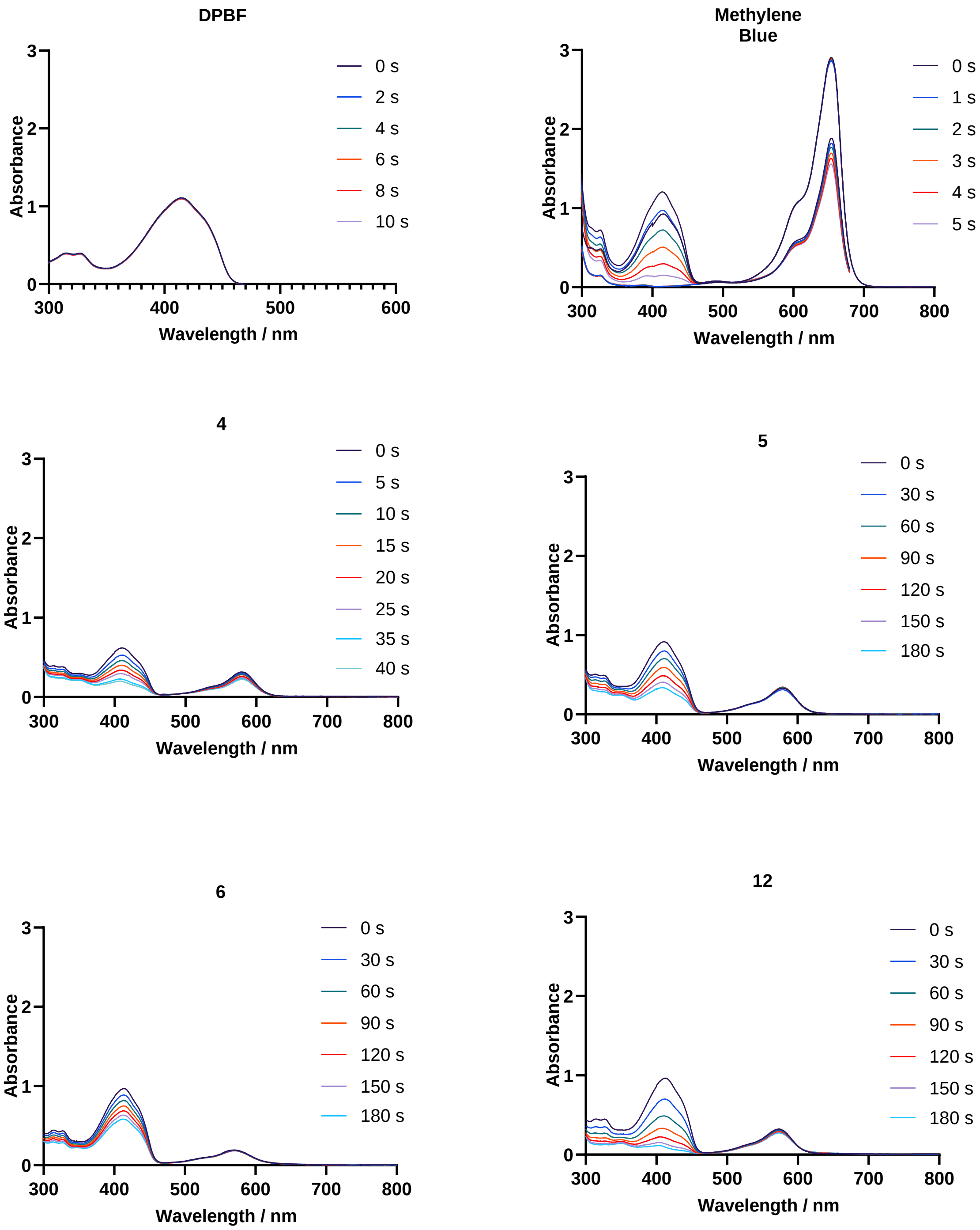

Figure S12. Changes in the absorption spectra of DPBF resulting from the irradiation with green light (505±35 nm, 100 mW cm^{-2}) in the absence or in the presence of COUPY-caged compounds **4-6**, coumarin alcohol **12** or methylene blue as reference.

5.- Irradiation experiments

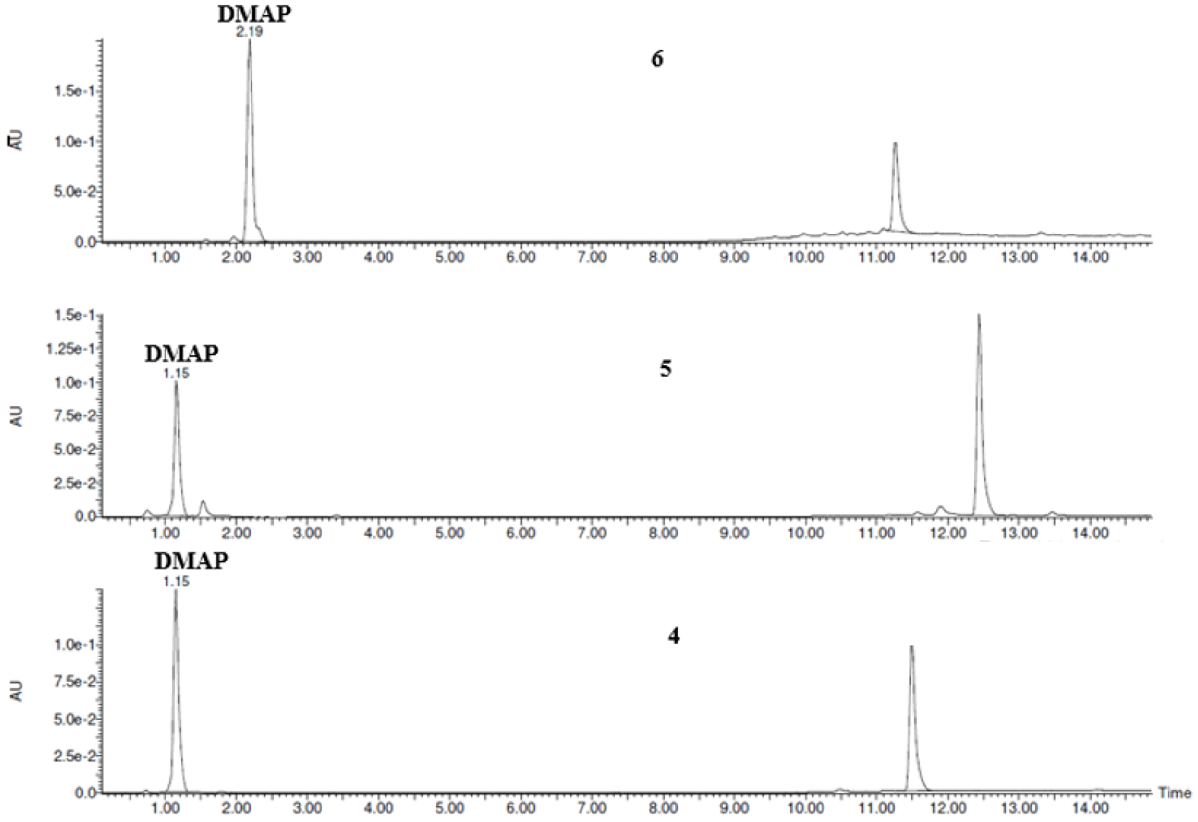

Figure S13. Reversed-phase HPLC-ESI MS traces at 260 nm of COUPY-caged compounds **4-6** after standing for 2 h in the dark at 37 ºC in a 8:2 (v/v) mixture of PBS buffer and ACN in the presence of DMAP (internal standard) using column 2.

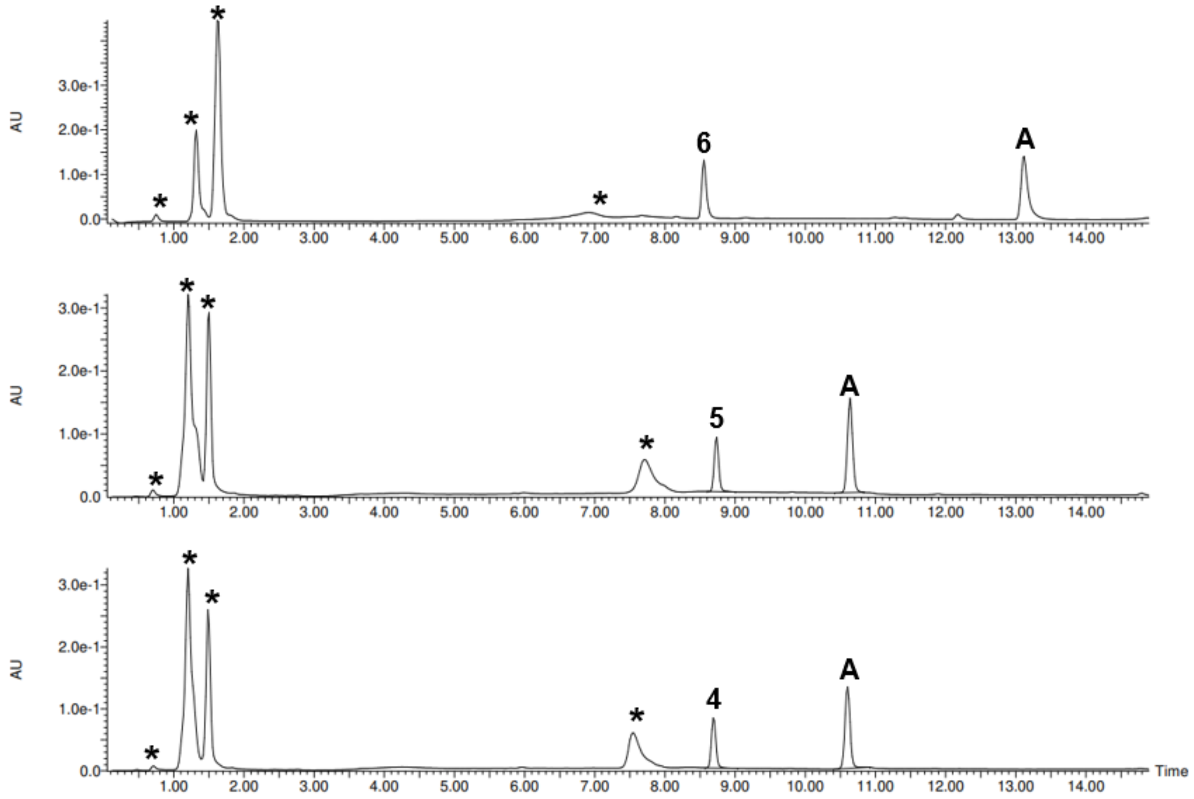

Figure S14. Reversed-phase HPLC-ESI MS traces at 260 nm of COUPY-caged compounds **4-6** after standing 2 h in the dark at 37 ºC in supplemented DMEM with 10% FBS and 1% P/S in the presence of coumarin **A** (internal standard) using column 2. Internal standard **A** corresponds to 1,1,6,6,8-Pentamethyl-2,3,5,6-tetrahydro-1H,4H-11-oxa-3a-aza-benzo[de]anthracen-10-one. *Peaks corresponding to culture medium.

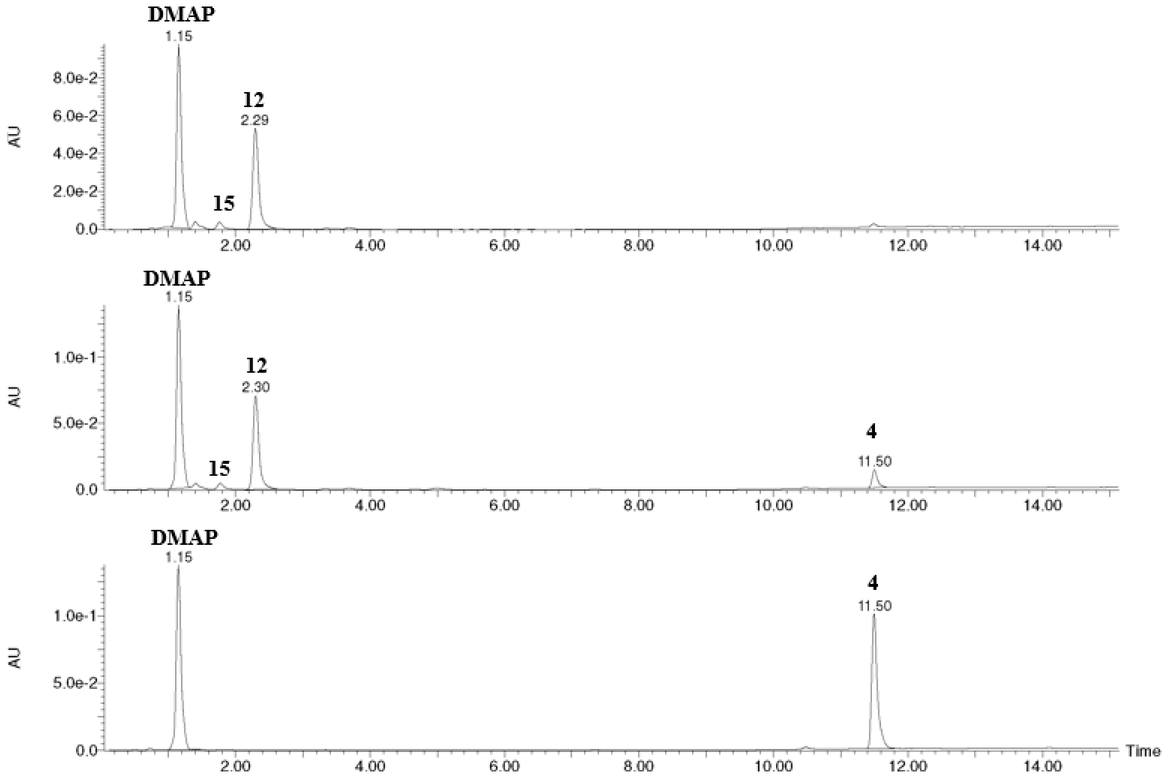

Figure S15. Reversed-phase HPLC-ESI MS traces at 260 nm for the photolysis reaction of **4** in a 8:2 (v/v) mixture of PBS buffer and ACN in the presence of DMAP (internal standard) at t=0 (bottom) and after irradiation with visible LED light (470-750 nm range, centered at 530 nm) for 12 min (middle) and 25 min (top) at 37 ºC using column 2.

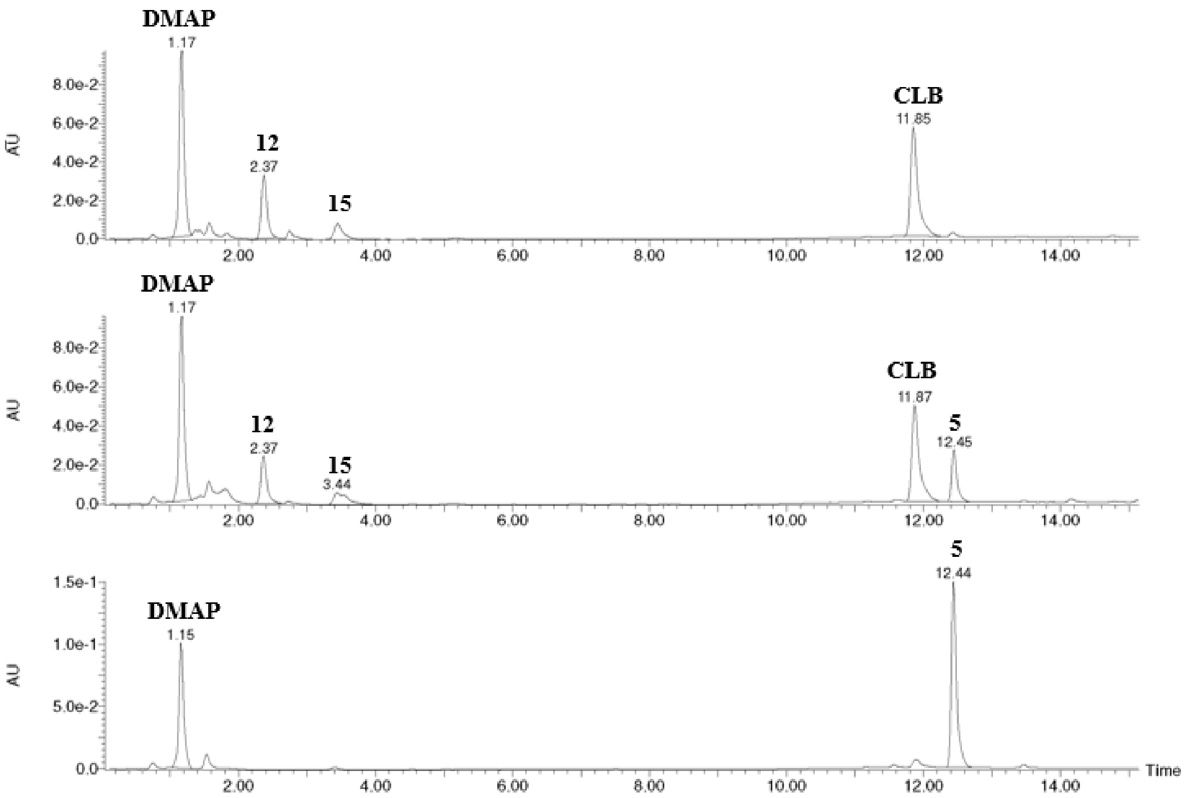

Figure S16. Reversed-phase HPLC-ESI MS traces at 260 nm for the photolysis reaction of **5** in a 8:2 (v/v) mixture of PBS buffer and ACN in the presence of DMAP (internal standard) at t=0 (bottom) and after irradiation with visible LED light (470-750 nm range, centered at 530 nm) for 30 min (middle) and 97 min (top) at 37 ºC using column 2.

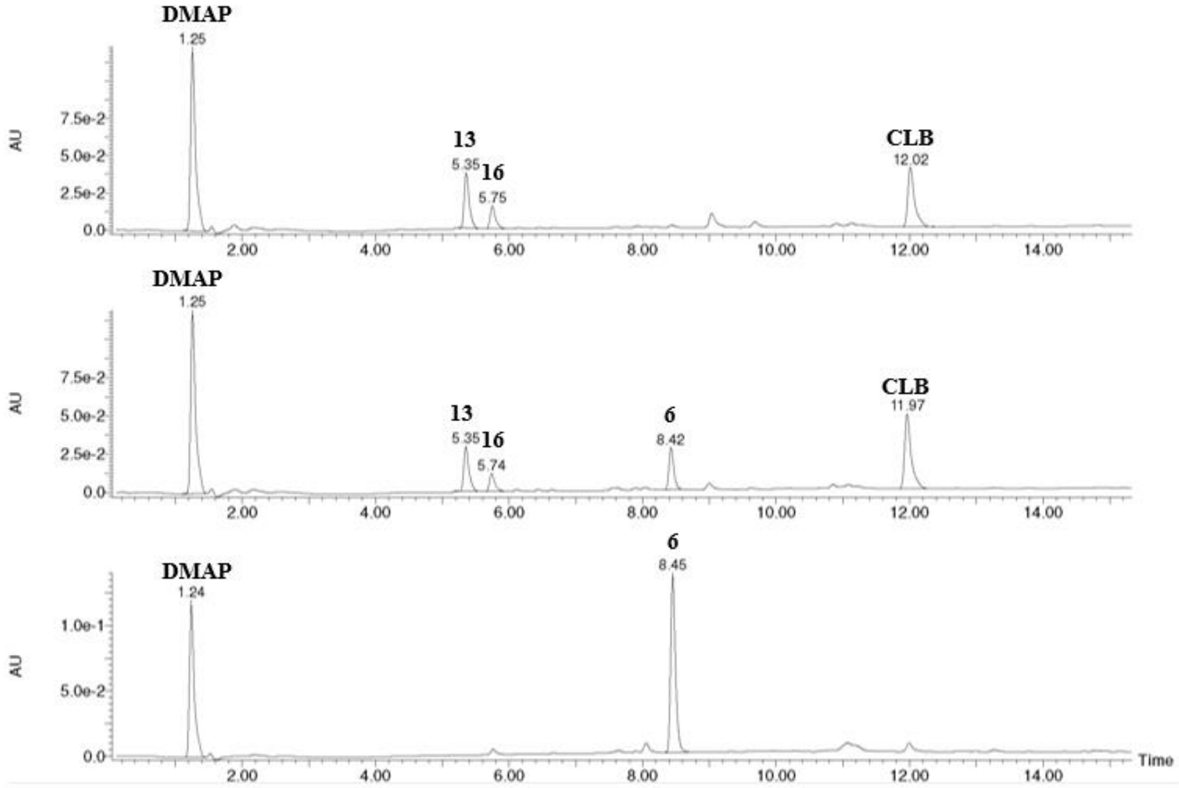

Figure S17. Reversed-phase HPLC-ESI MS traces at 260 nm for the photolysis reaction of **6** in a 8:2 (v/v) mixture of PBS buffer and ACN in the presence of DMAP (internal standard) at t=0 (bottom) and after irradiation with visible LED light (470-750 nm range, centered at 530 nm) for 30 min (middle) and 120 min (top) at 37 ºC using column 2.

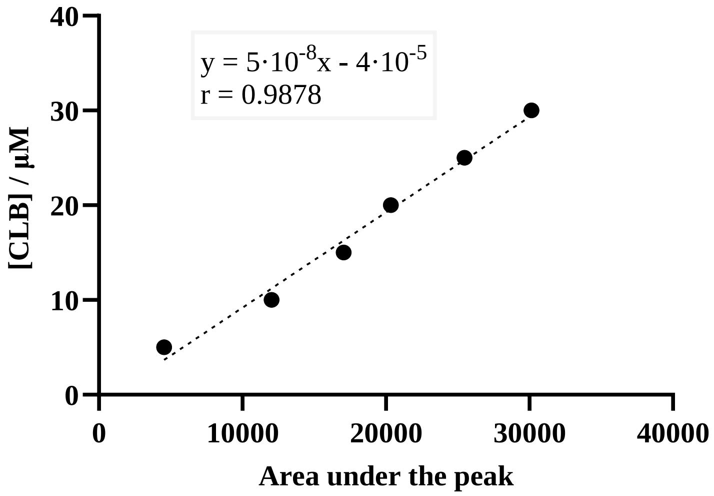

Figure S18. CLB calibration curve used for 5–30 µM range concentration.

6.- Determination of uncaging quantum yield (Φ_{Phot}).

To calculate the uncaging quantum yield of COUPY photocages **4-6**, first the photon flux of the light source was determined by actinometry following a previously reported procedure.[1] The actinometrical measurements using [1,2-bis(2,4-dimethyl-5-phenyl-3-thienyl)perfluorocyclopentene] (DAE), which acts as a visible-light actinometer, involves two steps: (i) conversion of DAE OF (open form) to DAE CF (closed form) under UV irradiation (UV lamp, λ_{max} = 365 nm) for 30 min, and (ii) conversion of DAE CF to DAE OF under the desired visible light irradiation wavelength. As shown in Figure S19, DAE OF only exhibits one absorption band centered at 268 nm, while DAE CF shows two additional bands at 370 nm and 564 nm. Moreover, this can also be easily observed by the colour of the solution: DAE CF appears purple in hexanes, whereas DAE OF is colourless. Once the complete formation of DAE CF was confirmed, the sample was irradiated with visible light over a time range of 1 to 150 seconds to induce ring opening and the UV-Vis spectra were recorded (Figure S19).

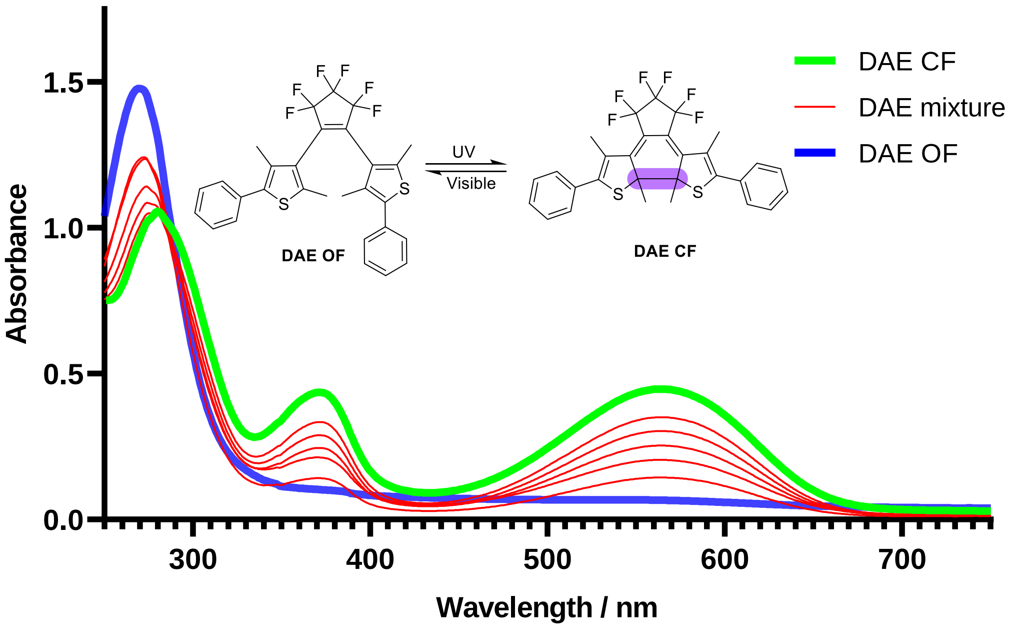

Figure S19. Absorption spectra of DAE OF solution in hexanes (30 µM) before UV irradiation (one band at 268 nm is observed) and of DAE CF after 30 min of UV irradiation (two additional bands at 370 and 564 nm are observed). After irradiation with visible light, the conversion of DAE CF to DAE OF is evidenced by the disappearance of the bands at 370 and 564 nm.

Once the photon flux (I_{abs}) of the light source (470-750 nm range, centered at 530 nm) was determined (6.19 x 10^{-7} Einstein s^{-1}),[2,3,4] the uncaging quantum yield (Φ_{Phot}) of COUPY photocages **4-6** was calculated using the following equation:

$$\frac{d[PPG]}{t} = -\phi_{\mathrm{Phot}} \frac{I_{absco}}{V}$$

in which I_{absco} represents the corrected photon flux that only considers the number of photons absorbed by the COUPY photocage and not by the photoreleased coumarin alcohol (Figure S20).

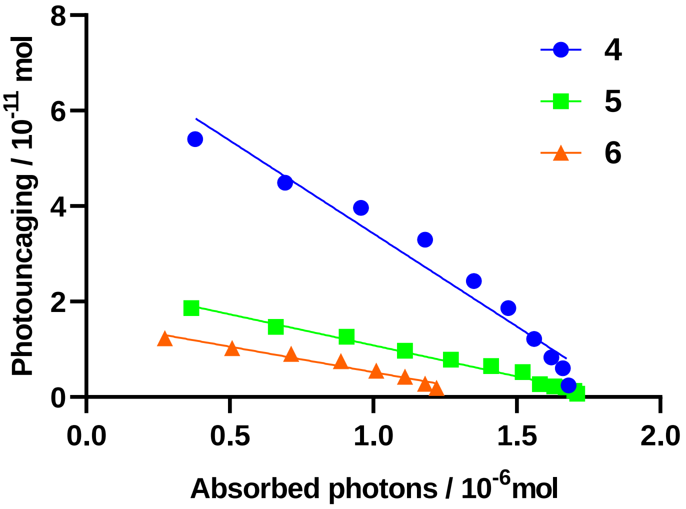

Figure S20. Determination of uncaging quantum yields from the decrease of COUPY photocages **4-6** as absorbed light dose increases.

7.- Confocal microscopy studies

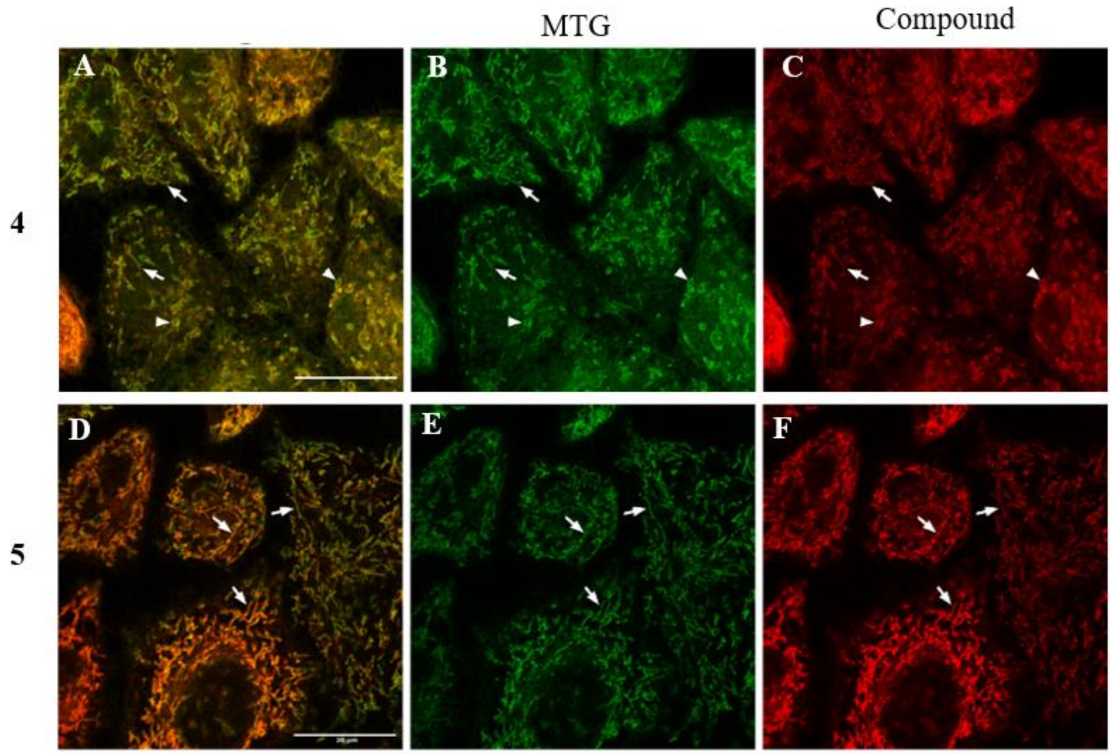

Figure S21. Colocalization studies with COUPY-caged compounds **4** (top) or **5** (bottom) and Mitotracker Green FM. Single confocal plane of HeLa cells incubated with **4** or **5** (1 μM, red) and MTG (0.1 μM, green). (A, D) Overlay of the two stainings. (C, F) Compounds **4** and **5'** signals. (B, E) MTG signal. White arrows and arrowheads point out elongated and donut mitochondria, respectively, colocalizing with Mitotracker staining. Scale bar: 20 μm.

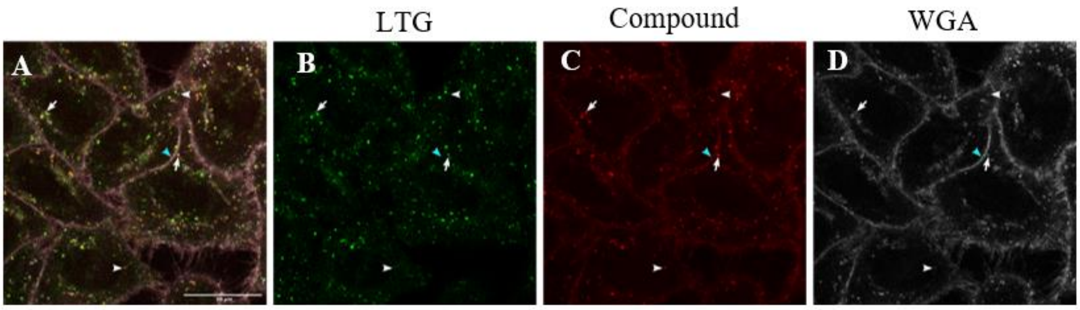

Figure S22. Colocalization studies with COUPY-caged compound **6** and LysoTracker Green FM (LTG) and Wheat Germ Agglutinin Alexa Fluor 633 (WGA). Single confocal plane of HeLa cells incubated with **6** (1 μM, red) and LTG (0.2 μM, green) and WGA (0.2 μg/mL). (A) Overlay of the three stainings. (C) Compound **6**' signal. (B, D) LTG and WGA signals, respectively. White arrows point out COUPY-photocage signal colocalized with lysosomes, white arrowheads with endosomes, and blue arrowheads with cytoplasmic membrane staining. Scale bar: 20 μm.

Table S2. Pearson's correlation coefficients (*r*) and Manders' overlap coefficients (M1 and M2) for the colocalization of COUPY photocages **4–6** with MitoTracker Green (MTG), LysoTracker Green (LTG), and Wheat Germ Agglutinin (WGA). M1 represents the fraction of the compound signal overlapping with the marker signal, while M2 represents the fraction of the marker signal overlapping with the compound signal.

**compound**	**MTG**			**LTG**			**WGA**		
	r	*M1*	*M2*	*r*	*M1*	*M2*	*r*	*M1*	*M2*
**4**	0.84	0.67	0.80	0.19	0.03	0.20	-	-	-
**5**	0.82	0.59	0.71	0.21	0.02	0.18	-	-	-
**6**	-	-	-	0.44	0.21	0.69	0.59	0.63	0.59

8.- (Photo)cytotoxicity studies

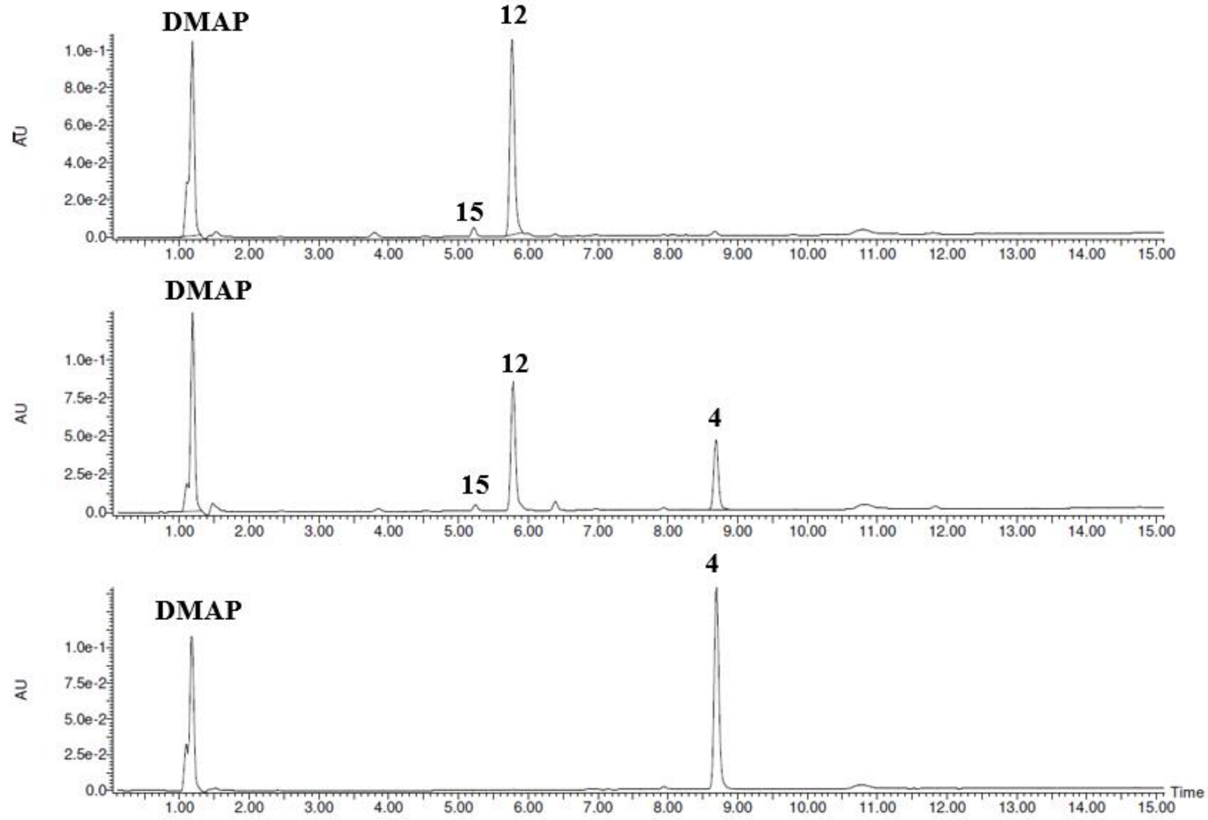

Figure S23. Reversed-phase HPLC-ESI MS traces at 260 nm for the photolysis reaction of **4** in a 8:2 (v/v) mixture of PBS buffer and ACN in the presence of DMAP (internal standard) at t=0 (bottom) and after irradiation with green-yellow LED light (550 nm, 4 mW/cm^2 pulsed light, 1 min ON, 1 min OFF) 5 min (middle) and 15 min (top) at 37 ºC using column 2. Irradiation was carried out directly in the 96-well plate.

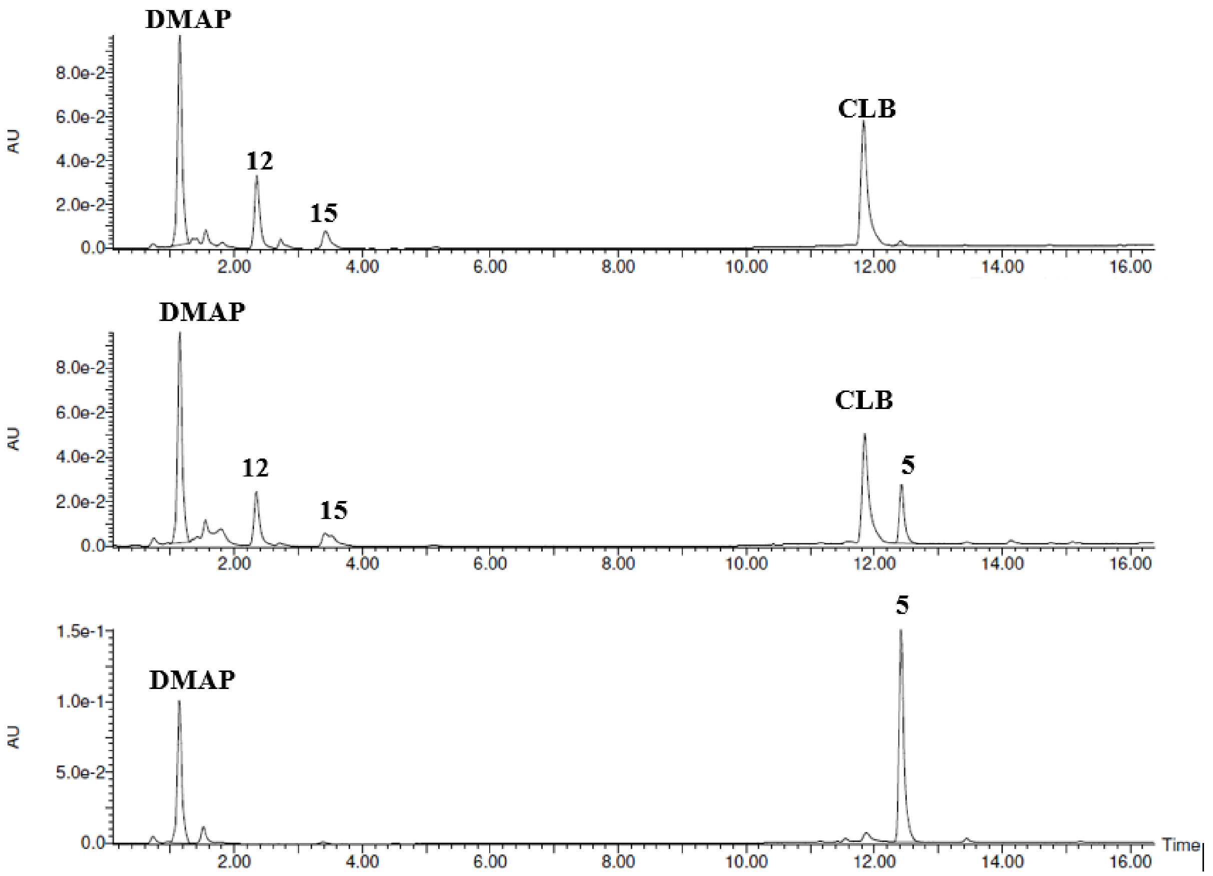

Figure S24. Reversed-phase HPLC-ESI MS traces at 260 nm for the photolysis reaction of **5** in a 8:2 (v/v) mixture of PBS buffer and ACN in the presence of DMAP (internal standard) at t=0 (bottom) and after irradiation with green-yellow with green-yellow LED light (550 nm, 4 mW/cm^2 pulsed light, 1 min ON, 1 min OFF) (middle) and for 15 min (middle) and 30 min (top) at 37 ºC using column 2. Irradiation was carried out directly in the 96-well plate.

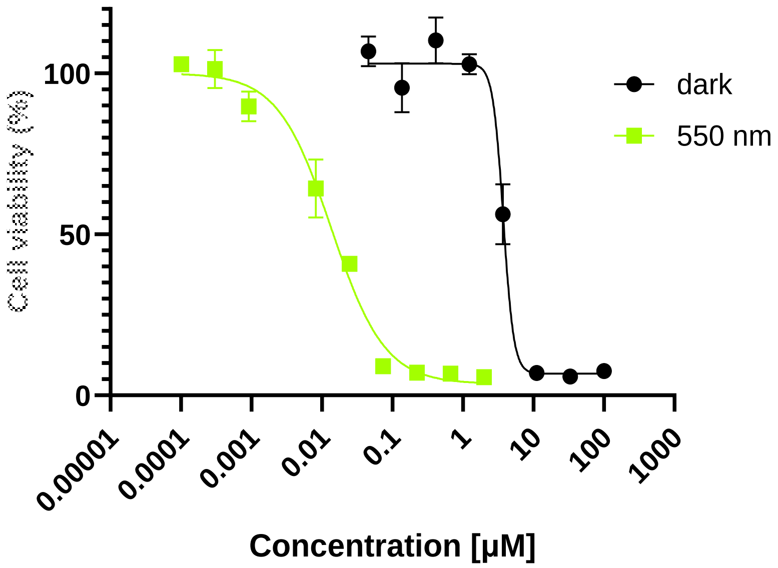

Figure S25. Cellular activity of **4**, measured as the percentage of live cells relative to the 0.5% DMSO control. Dose–response curves of **4** on the viability of HeLa cells (MTT assay) under either dark or irradiation (550 nm, 4 mW/cm^2 pulsed light, 1 min ON, 1 min OFF) conditions for 1 h.

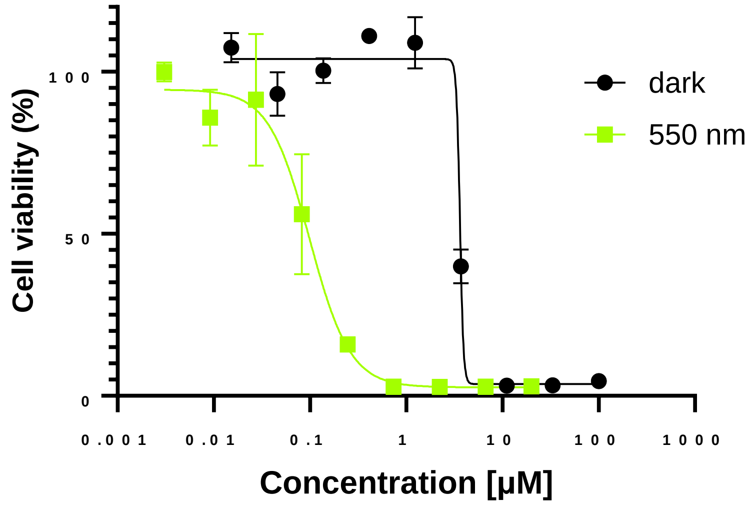

Figure S26. Cellular activity of **5**, measured as the percentage of live cells relative to the 0.5% DMSO control. Dose–response curves of **5** on the viability of HeLa cells (MTT assay) under either dark or irradiation (550 nm, 4 mW/cm^2 pulsed light, 1 min ON, 1 min OFF) conditions for 1 h.

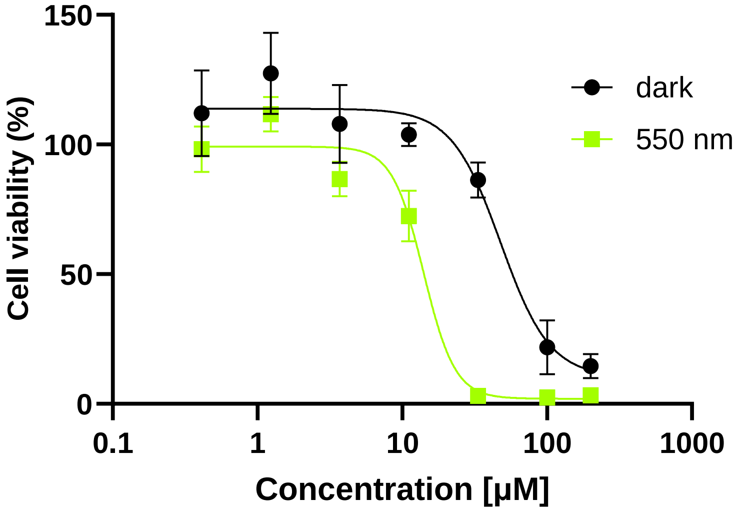

Figure S27. Cellular activity of **6**, measured as the percentage of live cells relative to the 0.5% DMSO control. Dose–response curves of **6** on the viability of HeLa cells (MTT assay) under either dark or irradiation (550 nm, 4 mW/cm^2 pulsed light, 1 min ON, 1 min OFF) conditions for 1 h.

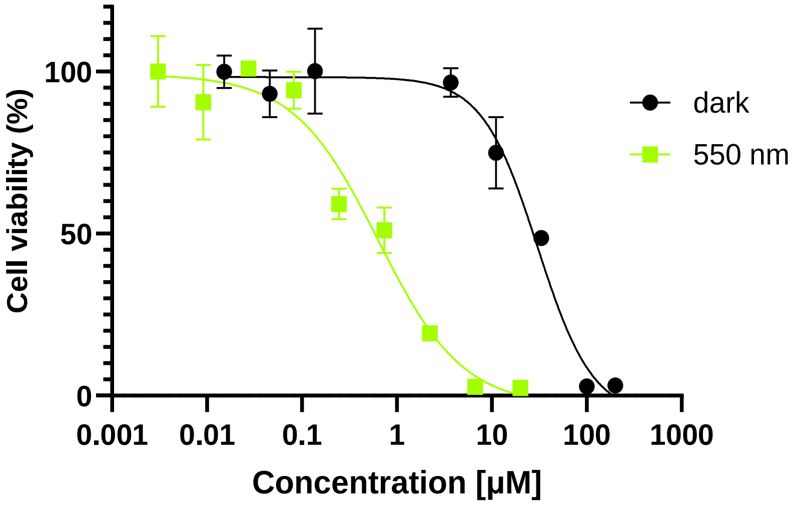

Figure S28. Cellular activity of **12**, measured as the percentage of live cells relative to the 0.5% DMSO control. Dose–response curves of **12** on the viability of HeLa cells (MTT assay) under either dark or irradiation (550 nm, 4 mW/cm^2 pulsed light, 1 min ON, 1 min OFF) conditions for 1 h.

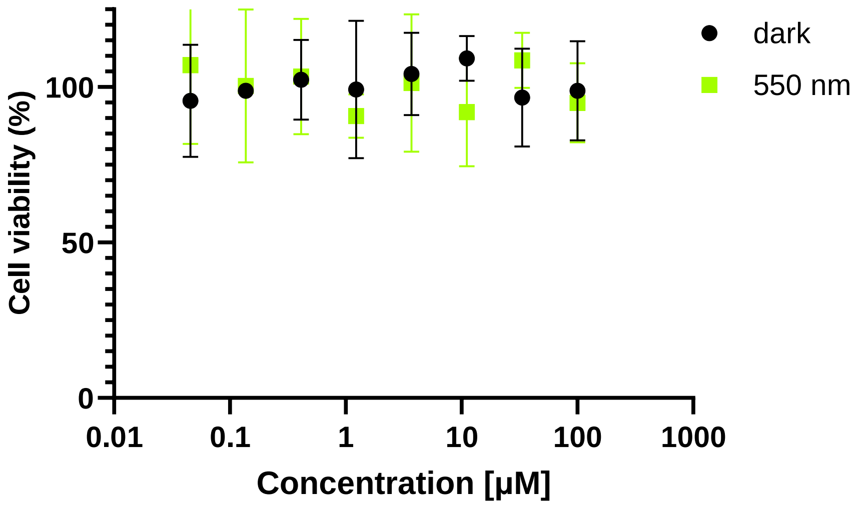

Figure S29. Cellular activity of 4-PBA, measured as the percentage of live cells relative to the 0.5% DMSO control. Dose–response curves of 4-PBA on the viability of HeLa cells (MTT assay) under either dark or irradiation (550 nm, 4 mW/cm^2 pulsed light, 1 min ON, 1 min OFF) conditions for 1 h.

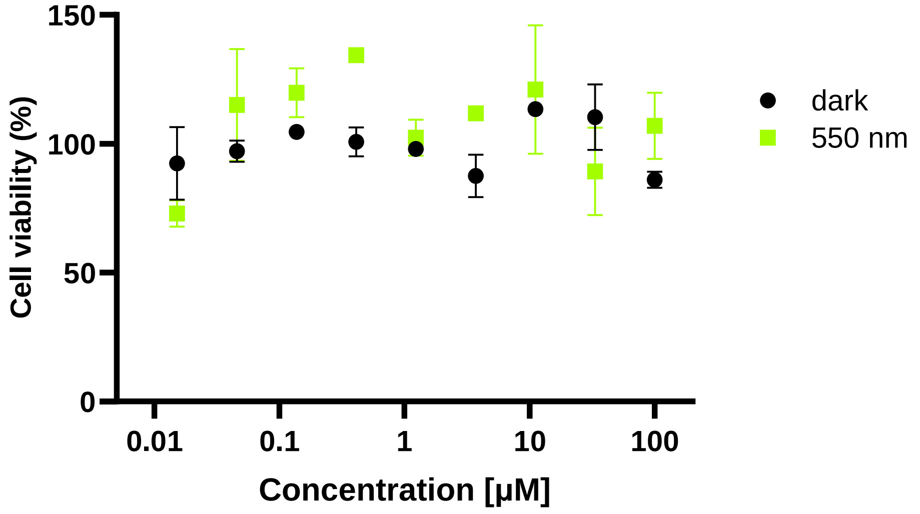

Figure S30. Cellular activity of CLB, measured as the percentage of live cells relative to the 0.5% DMSO control. Dose–response curves of CLB on the viability of HeLa cells (MTT assay) under either dark or irradiation (550 nm, 4 mW/cm^2 pulsed light, 1 min ON, 1 min OFF) conditions for 1 h.

Table S3. (Photo)cytotoxicity of compounds **5**, **12** and CLB towards U87-MG cells expressed as IC_{50} values [µM][a] and phototoxic indexes (PI)[b].

Compound	IC_{50} [µM] dark	IC_{50} [µM] light	PI
**5**	6.72 ± 0.81	0.12 ± 0.02	56
**12**	48.69 ± 4.62	0.66 ± 0.11	74
**CLB**	>100	>100	-

[a]Cells were treated for 2 h (1 h of incubation and 1 h of irradiation with green-yellow light (550 nm pulsed light (1 min ON, 1 min OFF), 7.2 J cm^{-2})) followed by 48 h recovery in drug-free medium. Control cells were left in the dark. IC_{50} values represent the mean of three independent experiments.
[b]Phototoxic index (PI) = IC_{50} (dark)/IC_{50} (irradiated cells).

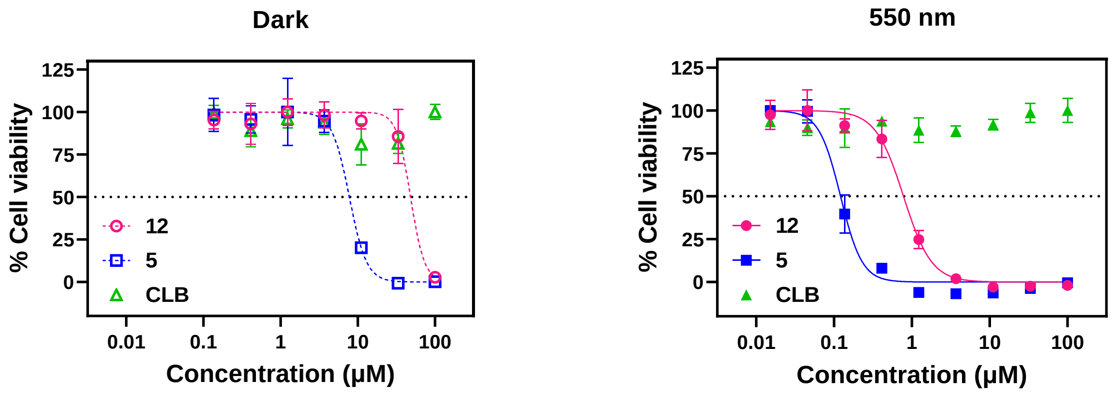

Figure S31. Cellular activity of compounds **5**, **12**, and **CLB**, expressed as the percentage of viable cells relative to the 0.5% DMSO control. Dose–response curves showing the effect of compounds **5**, **12**, and **CLB** on U87-MG cell viability, as assessed by the resazurin assay, under dark conditions or upon light irradiation (550 nm, 4 mW/cm², pulsed light: 1 min ON / 1 min OFF) for 1 hour.

9.- ^{1}H and ^{13}C NMR spectra and HR ESI-MS of the compounds

Compound 8

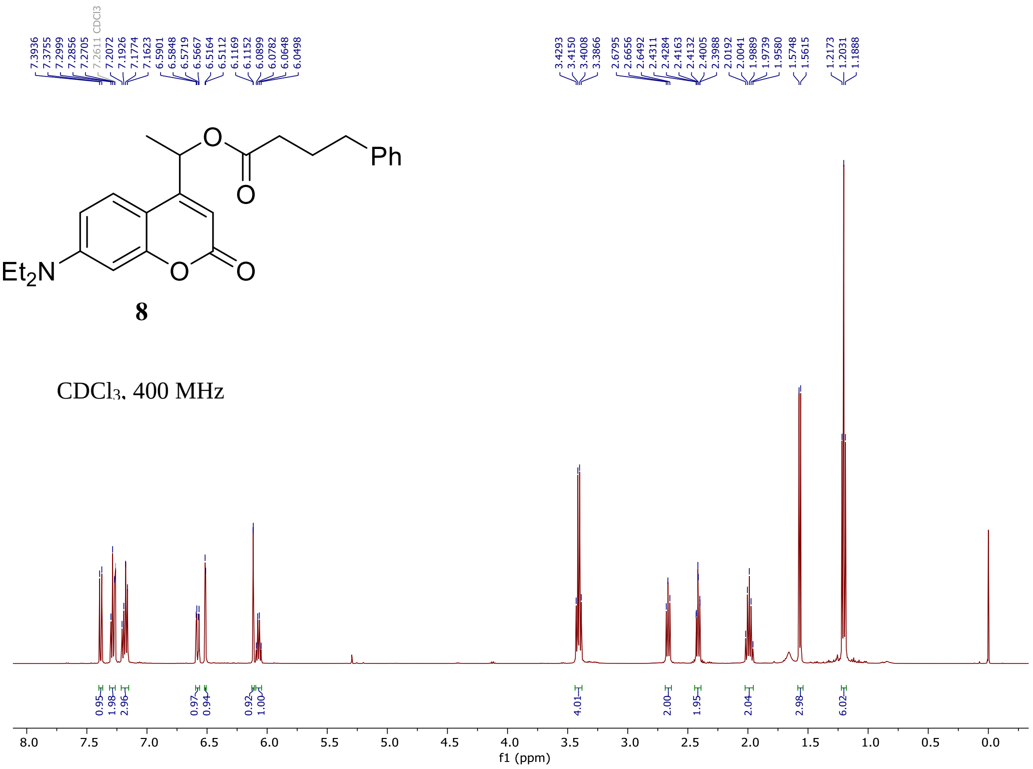

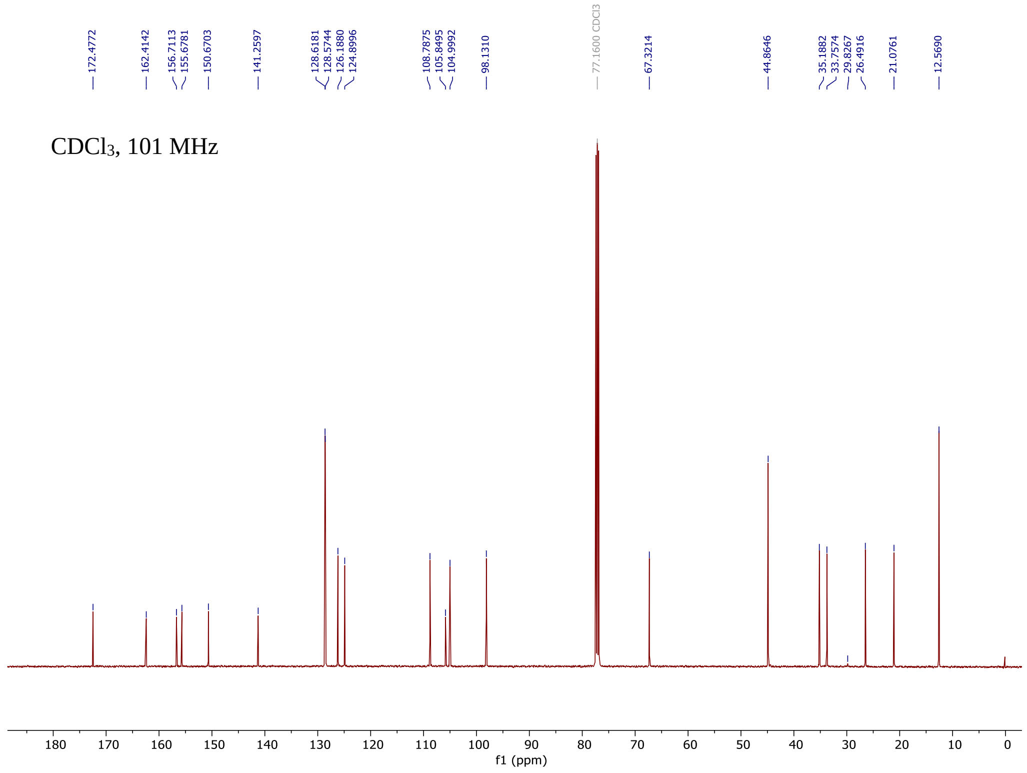

Figure S32. ^{1}H and ^{13}C NMR spectra of compound **8** in $CDCl_3$.

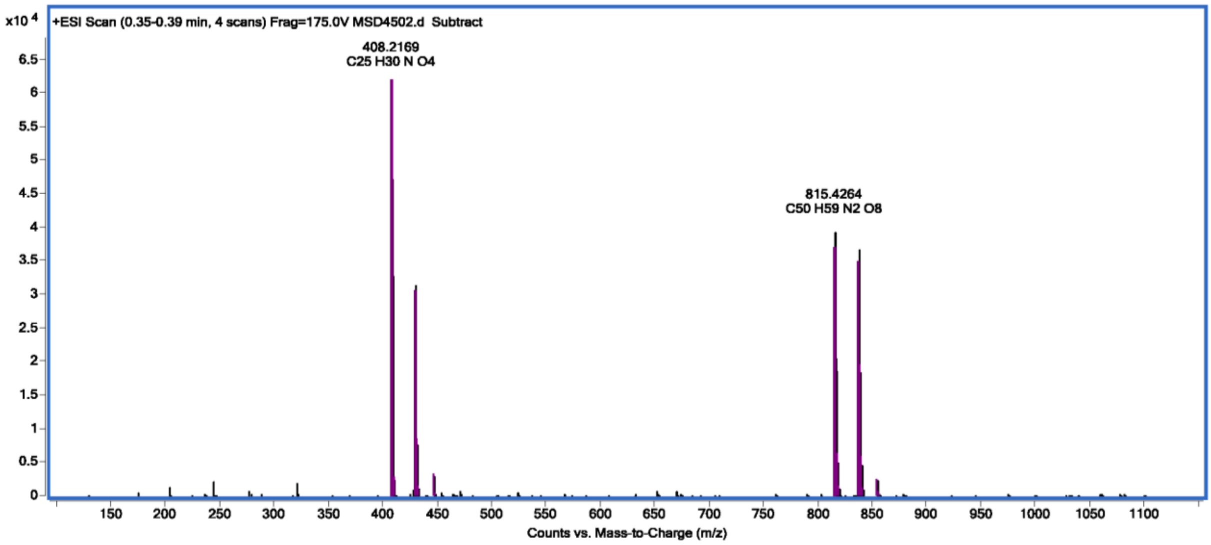

Figure S33. HR ESI-MS spectrum of compound **8**.

Compound 9

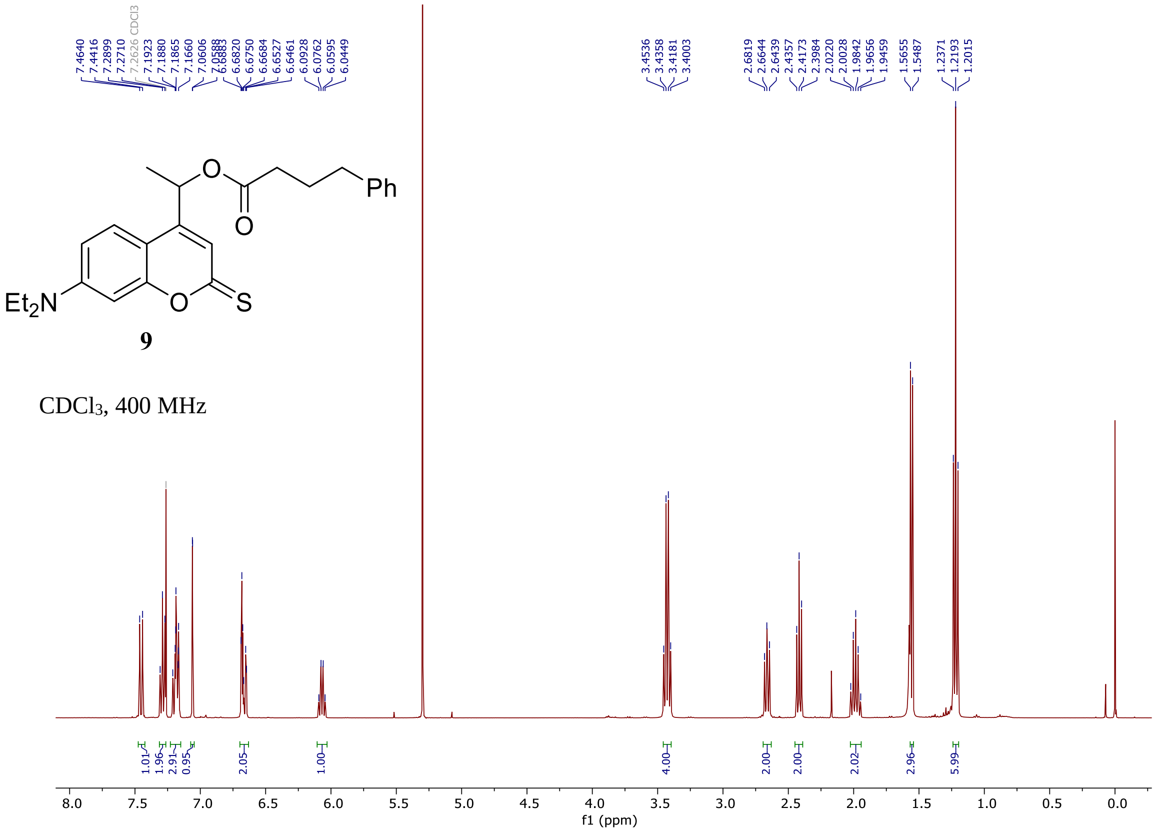

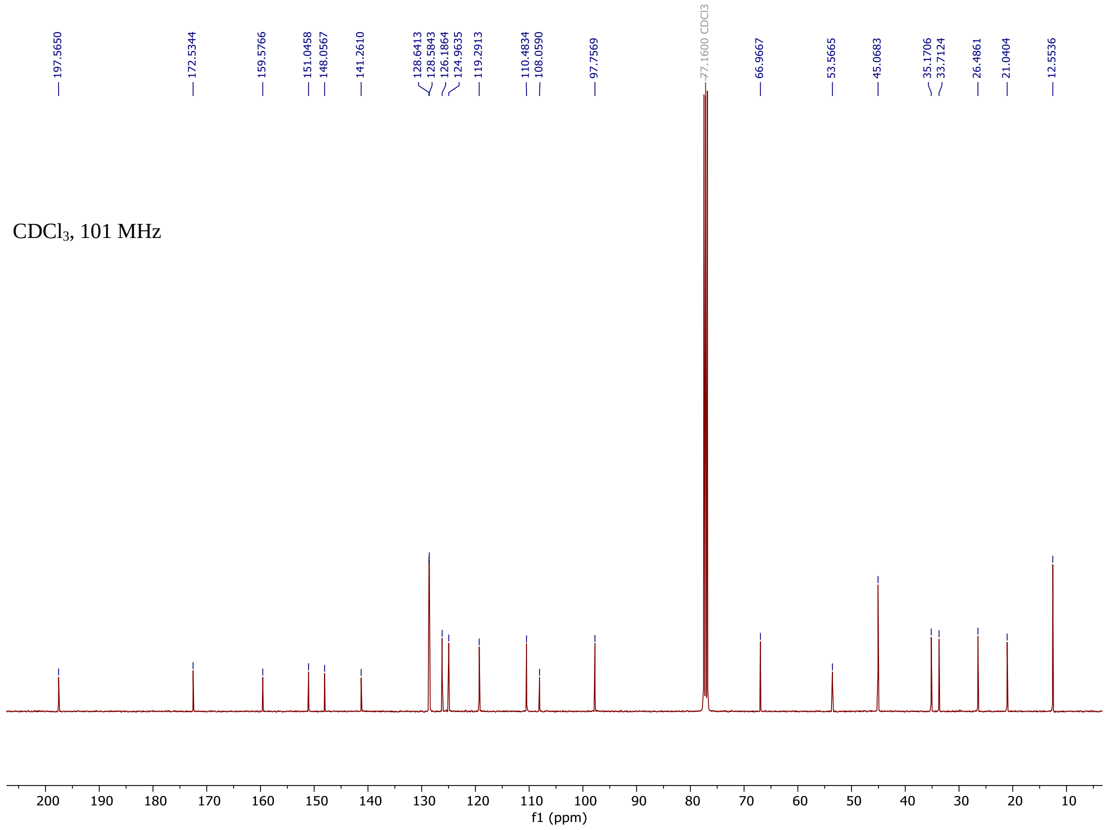

Figure S34. ^{1}H and ^{13}C NMR spectra of compound **9** in $CDCl_3$.

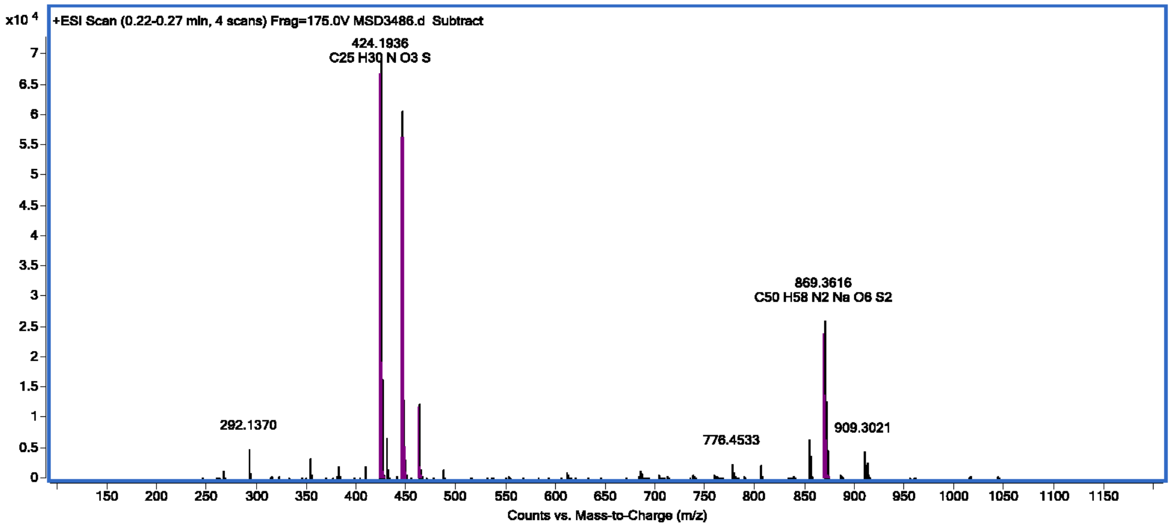

Figure S35. HR ESI-MS spectrum of compound **9**.

Compound 10

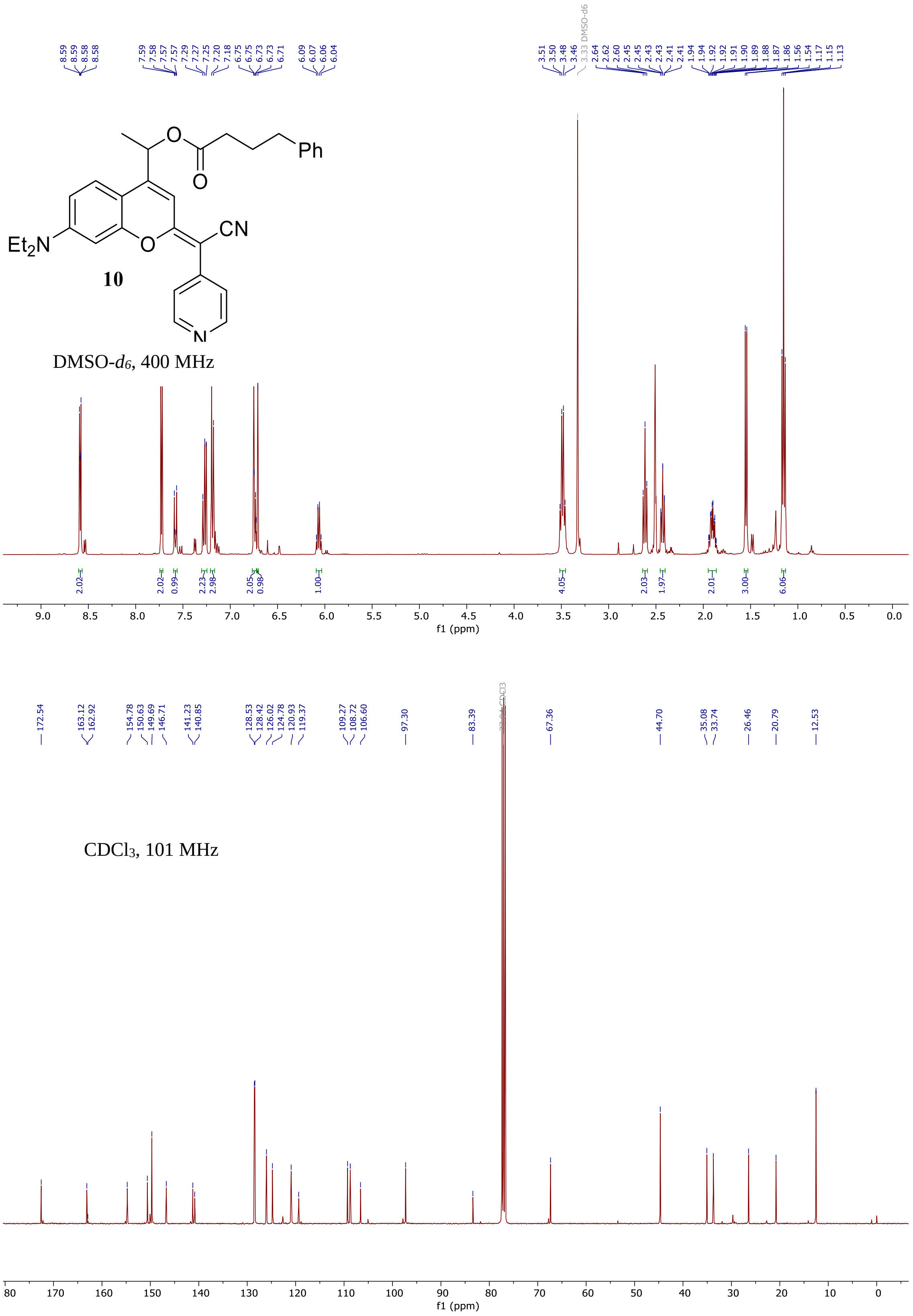

Figure S36. ^{1}H and ^{13}C NMR spectra of compound **10** in DMSO-d_6 and $CDCl_3$, respectively.

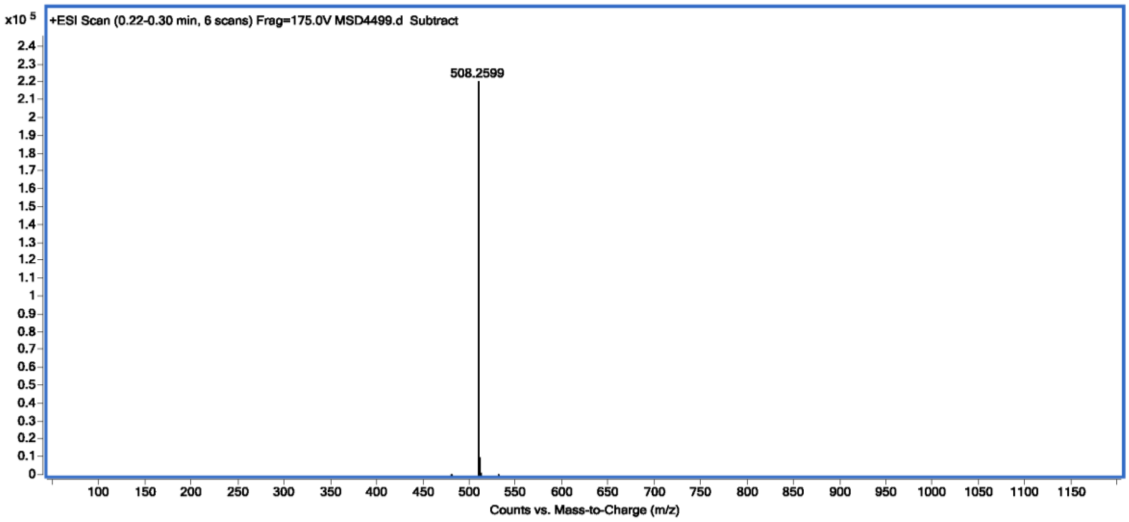

Figure S37. HR ESI-MS spectrum of compound **10**.

Compound 11

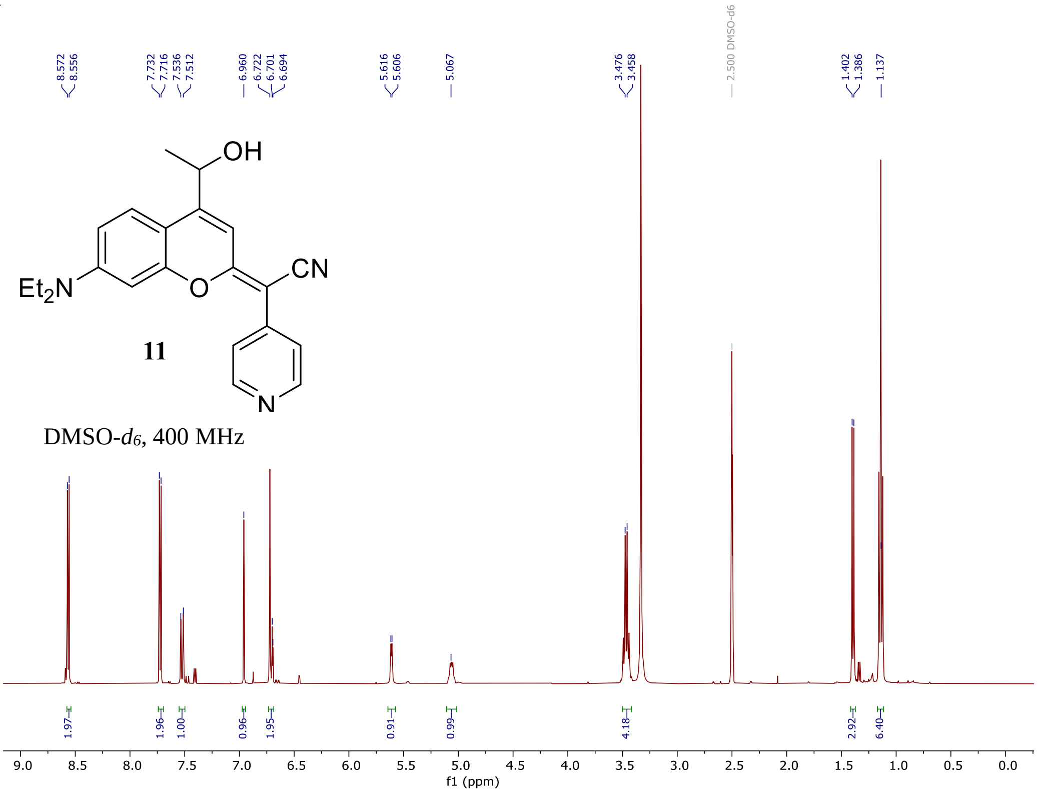

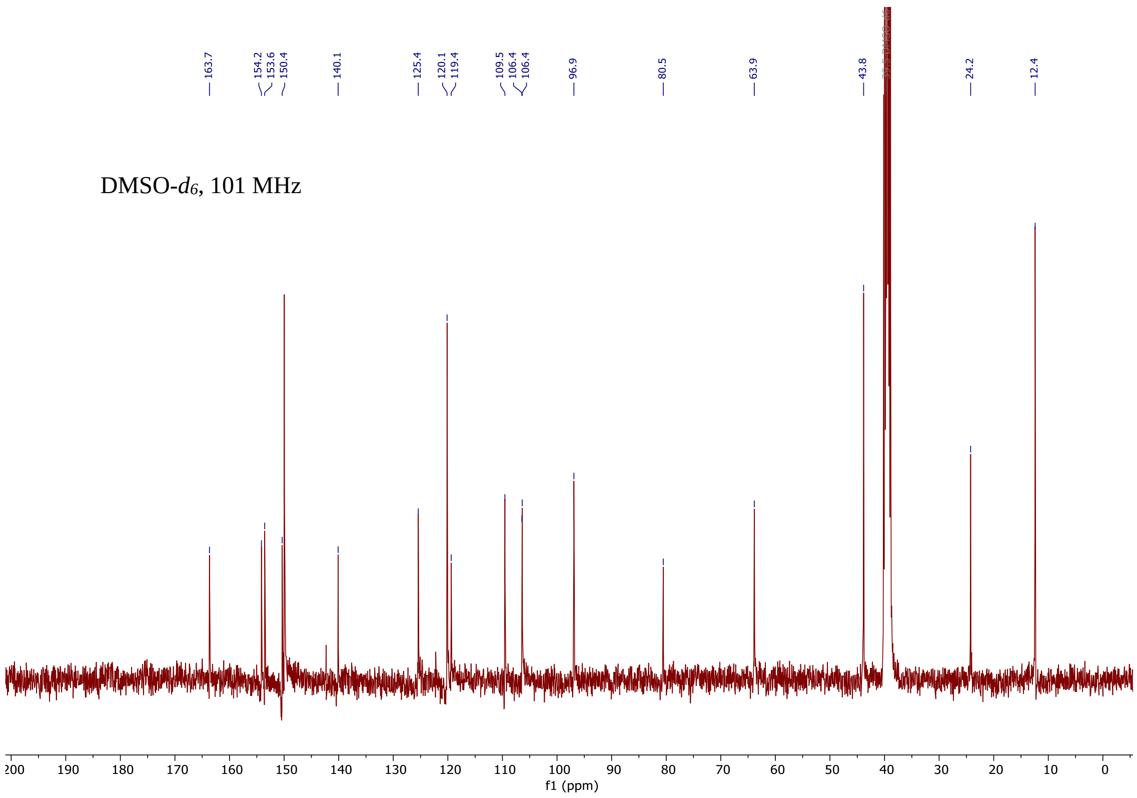

Figure S38. ^{1}H and ^{13}C NMR spectra of compound **11** in DMSO-d_6.

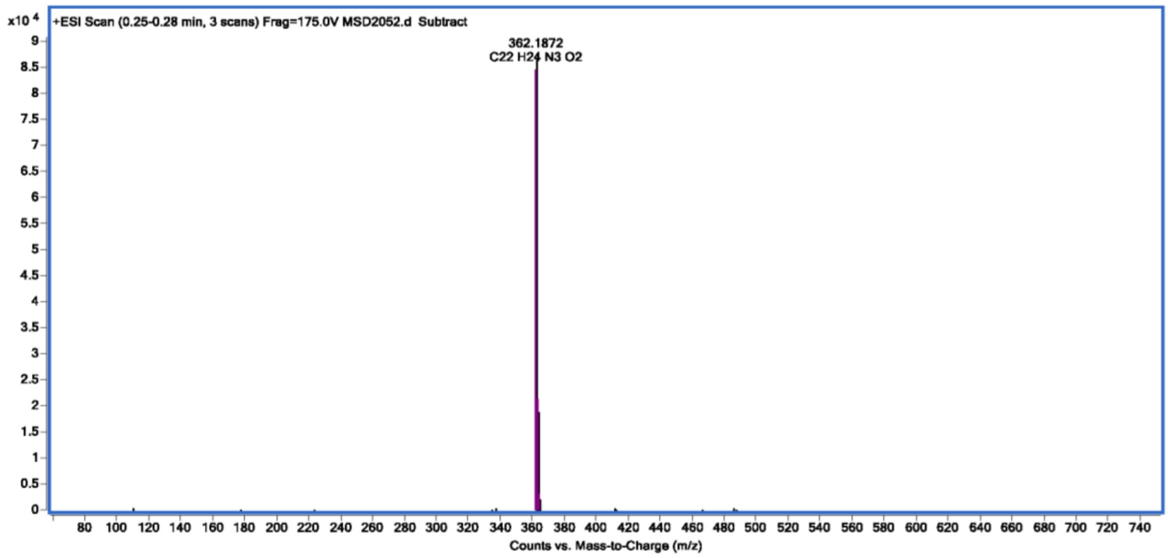

Figure S39. HR ESI-MS spectrum of compound **11**.

Compound 13

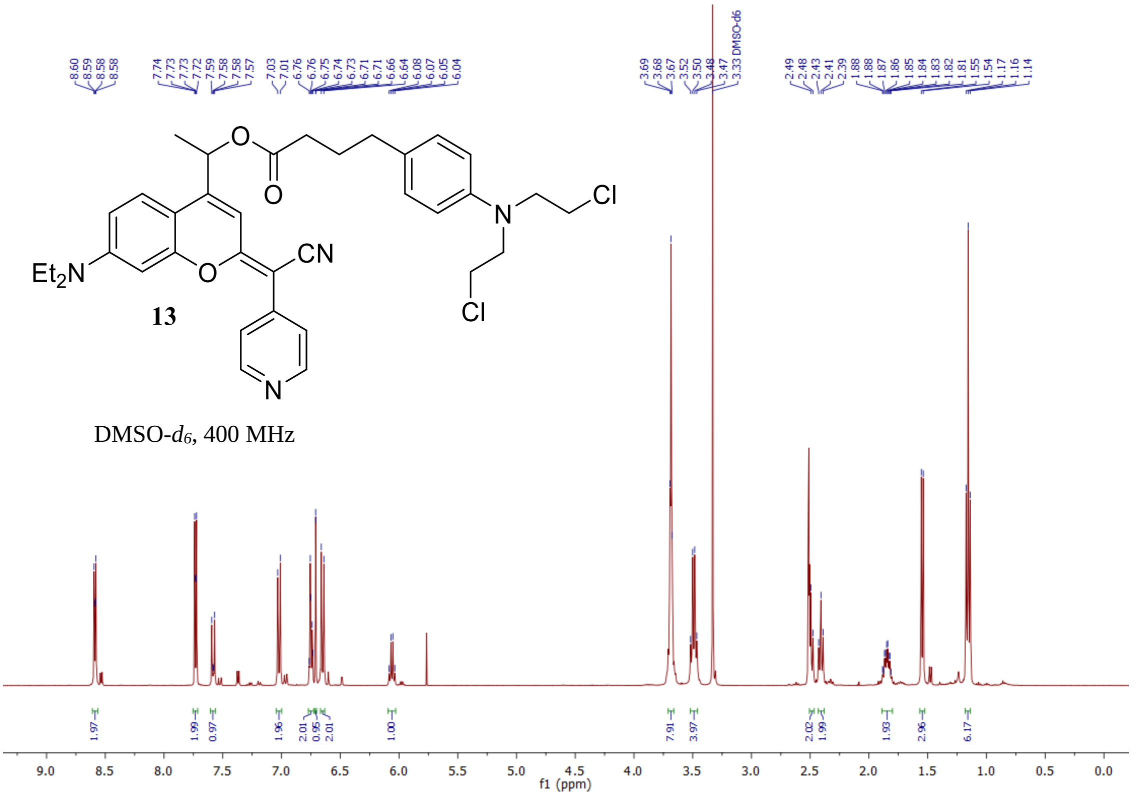

DMSO-d_6, 101 MHz

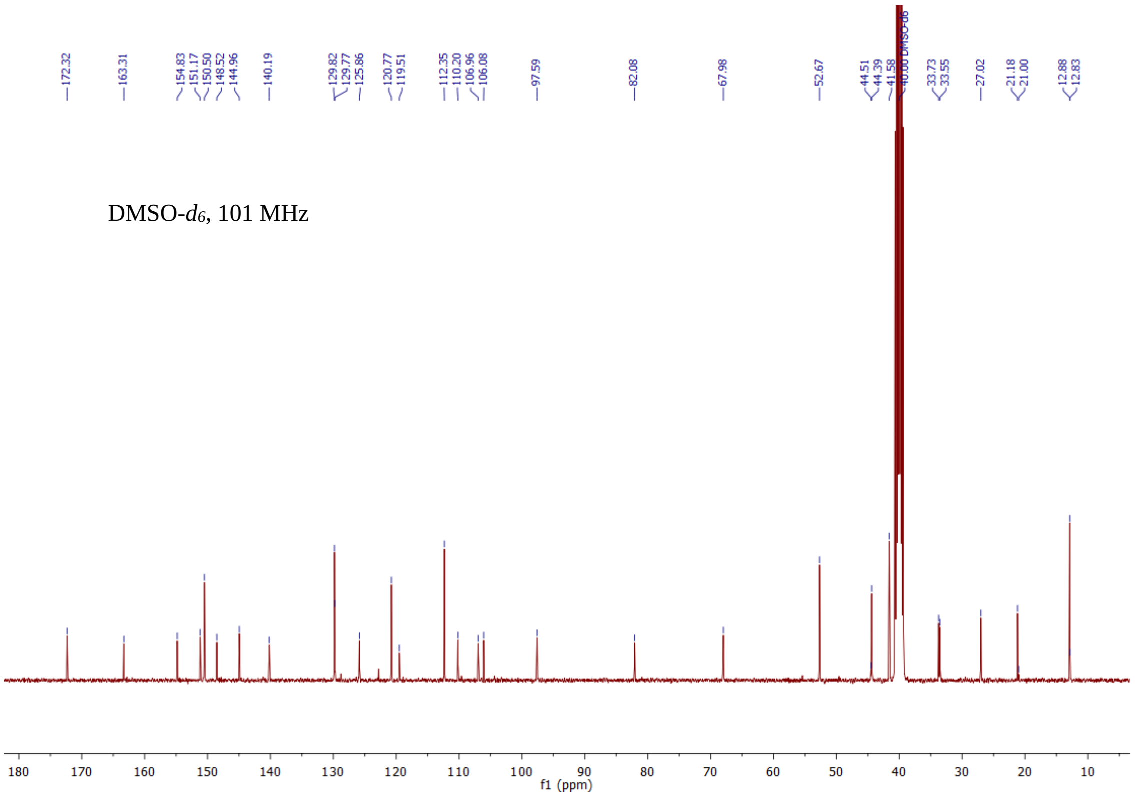

Figure S40. ^{1}H and ^{13}C NMR spectra of compound **13** in DMSO-d_6.

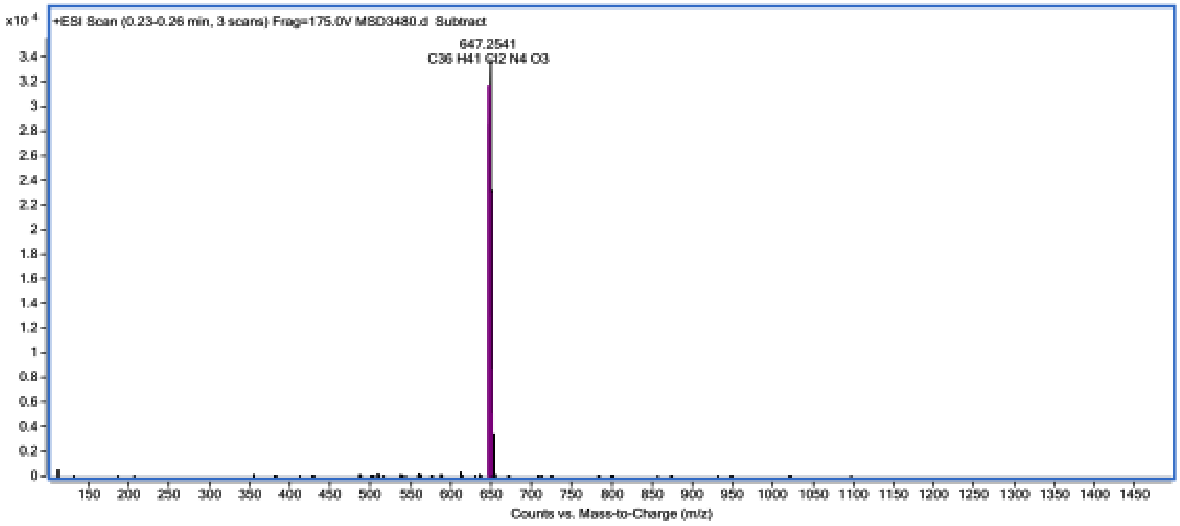

Figure S41. HR ESI-MS spectrum of compound **13**.

Compound 4

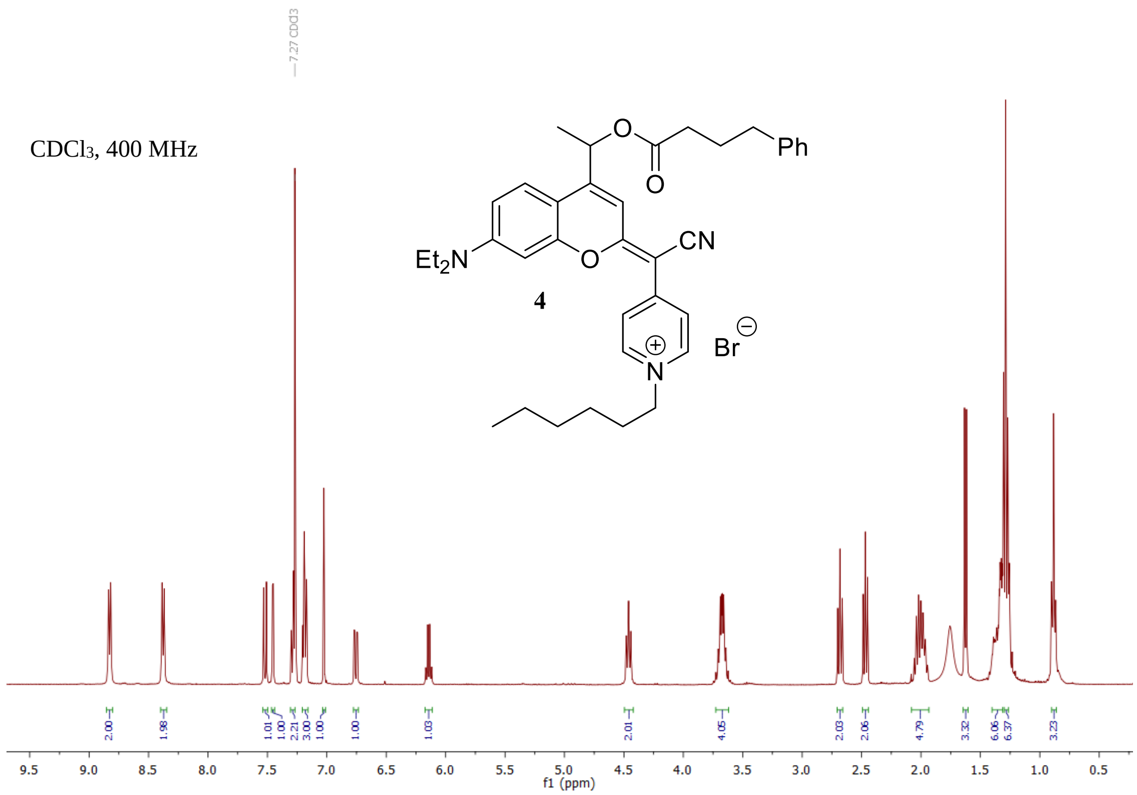

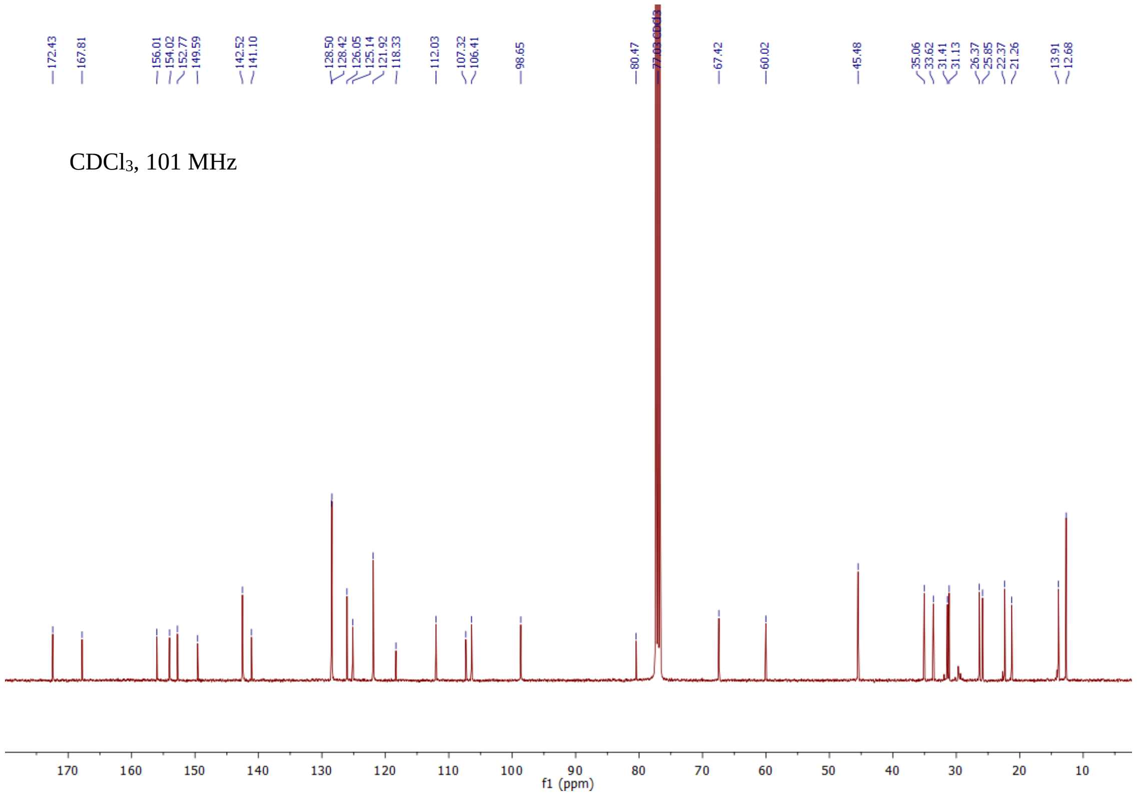

Figure S42. ^{1}H and ^{13}C NMR spectra of compound **4** in $CDCl_3$.

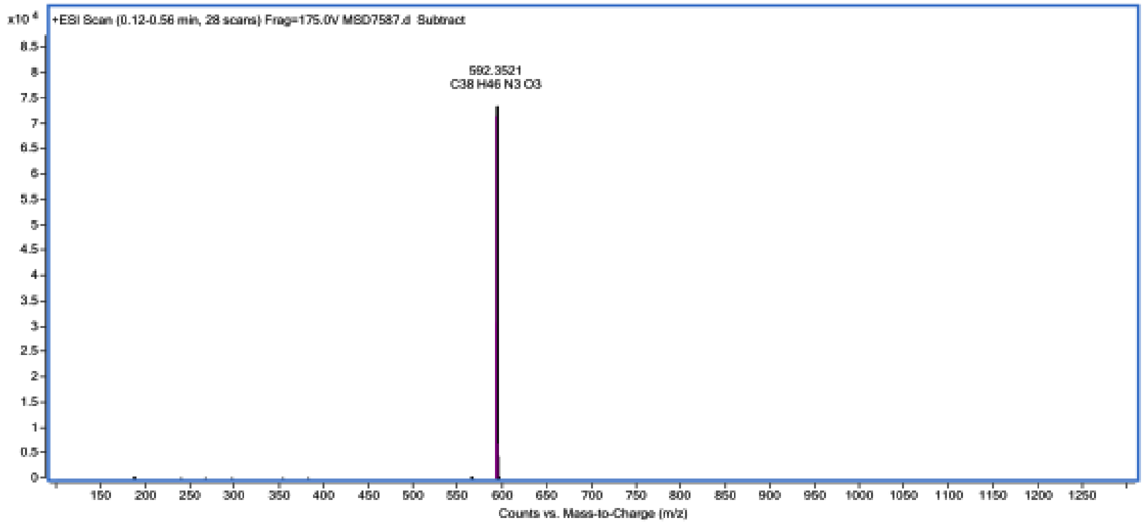

Figure S43. HR ESI-MS spectrum of compound **4**.

Compound 5

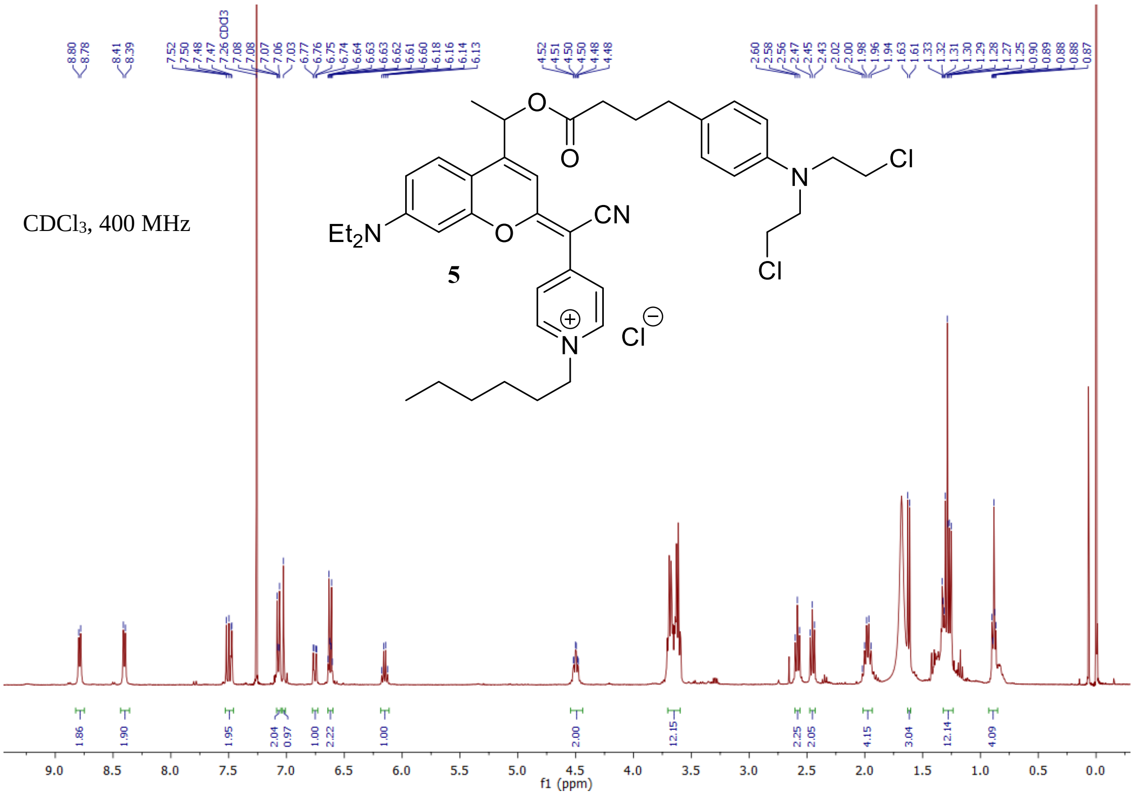

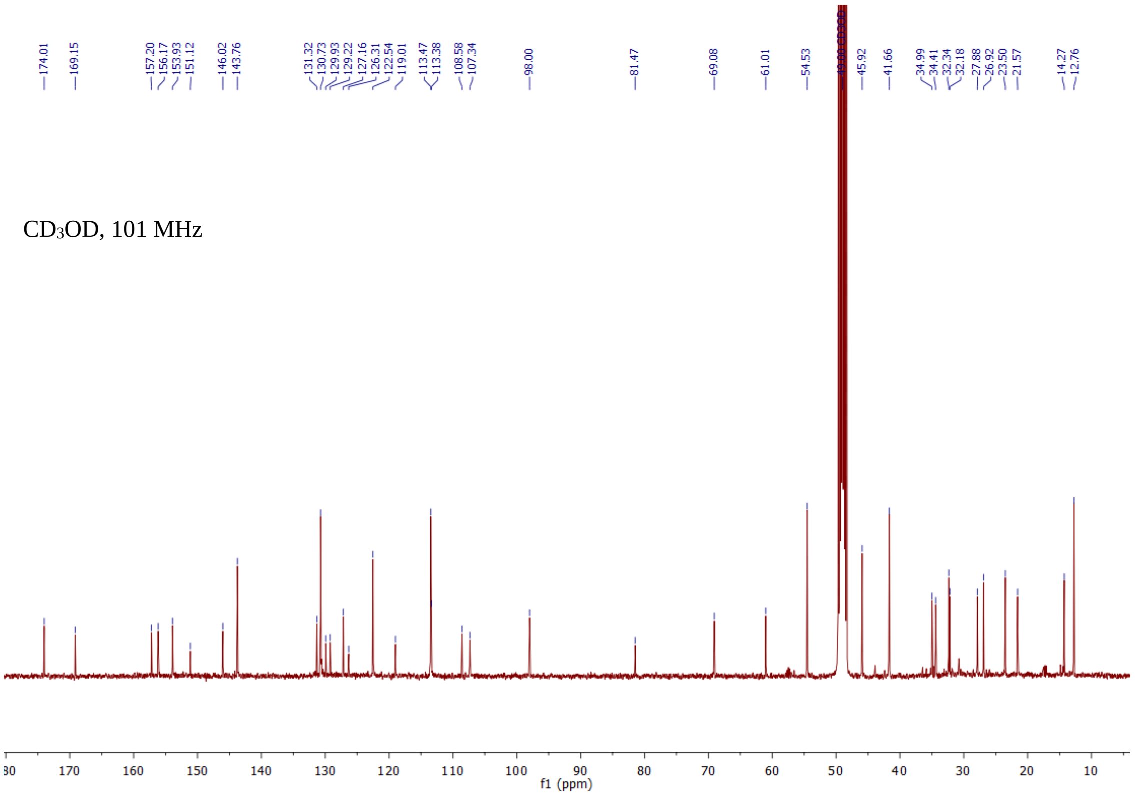

Figure S44. ^{1}H and ^{13}C NMR spectra of compound **5** in $CDCl_3$ and CD_3OD, respectively.

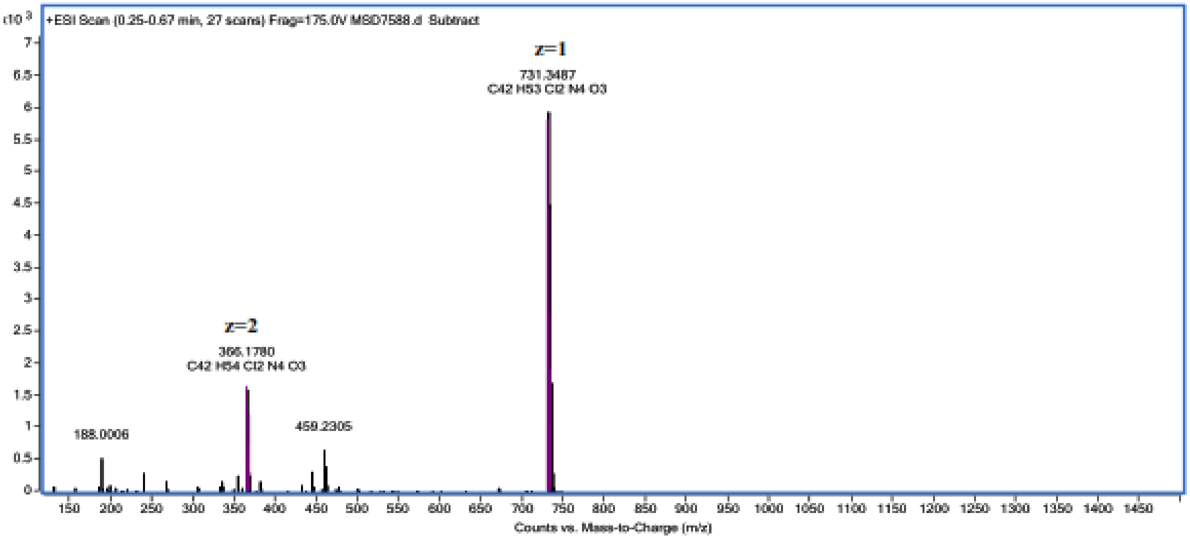

Figure S45. HR ESI-MS spectrum of compound **5**.

Compound 6

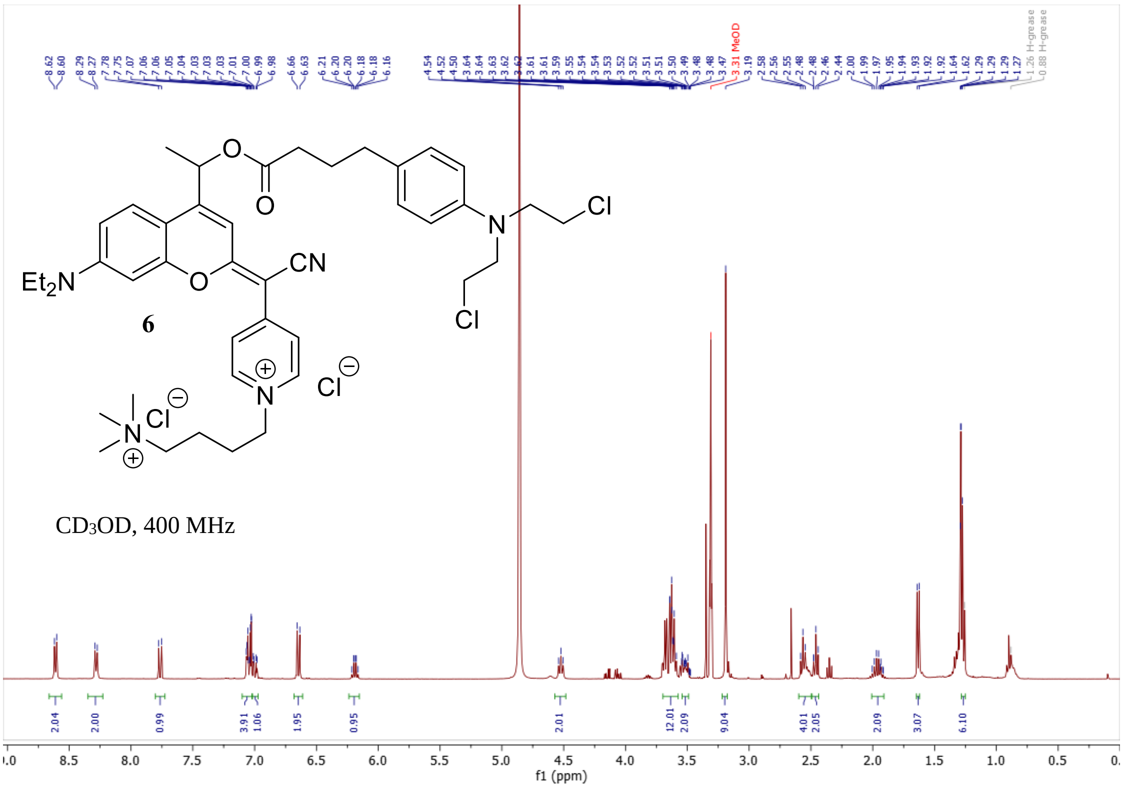

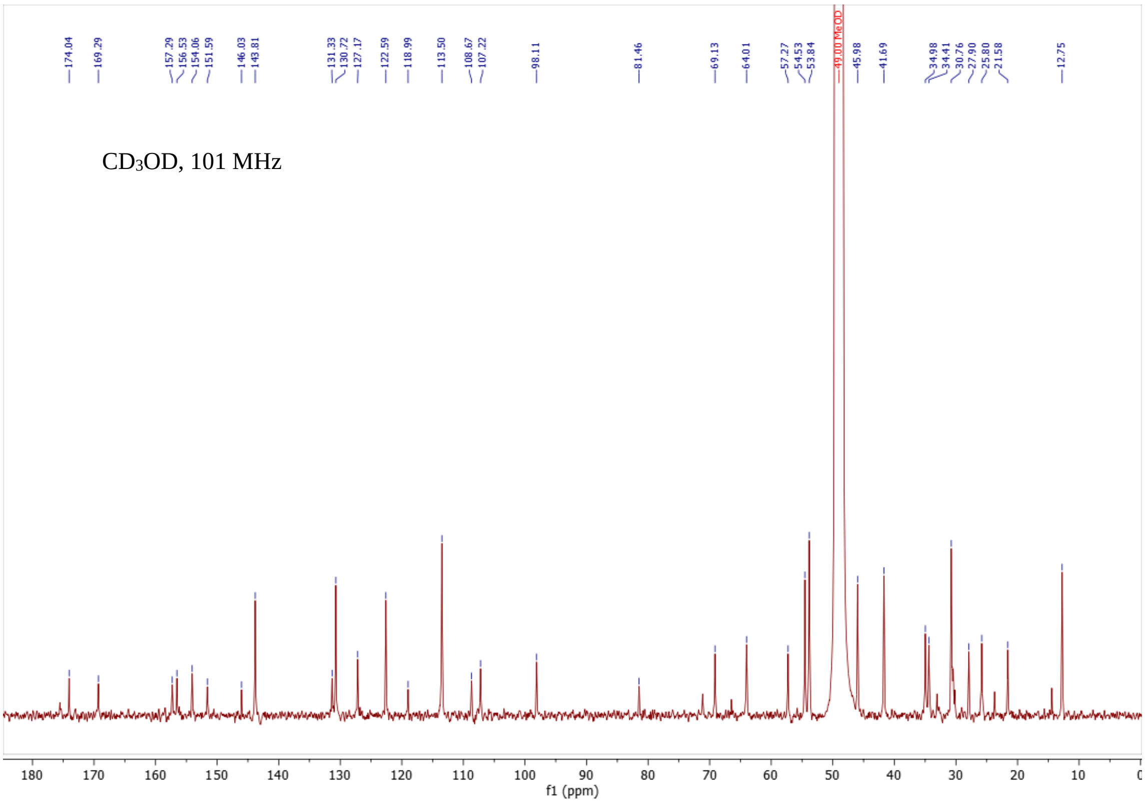

Figure S46. ^{1}H and^{13}C NMR spectra of compound **6** in $CDCl_3$.

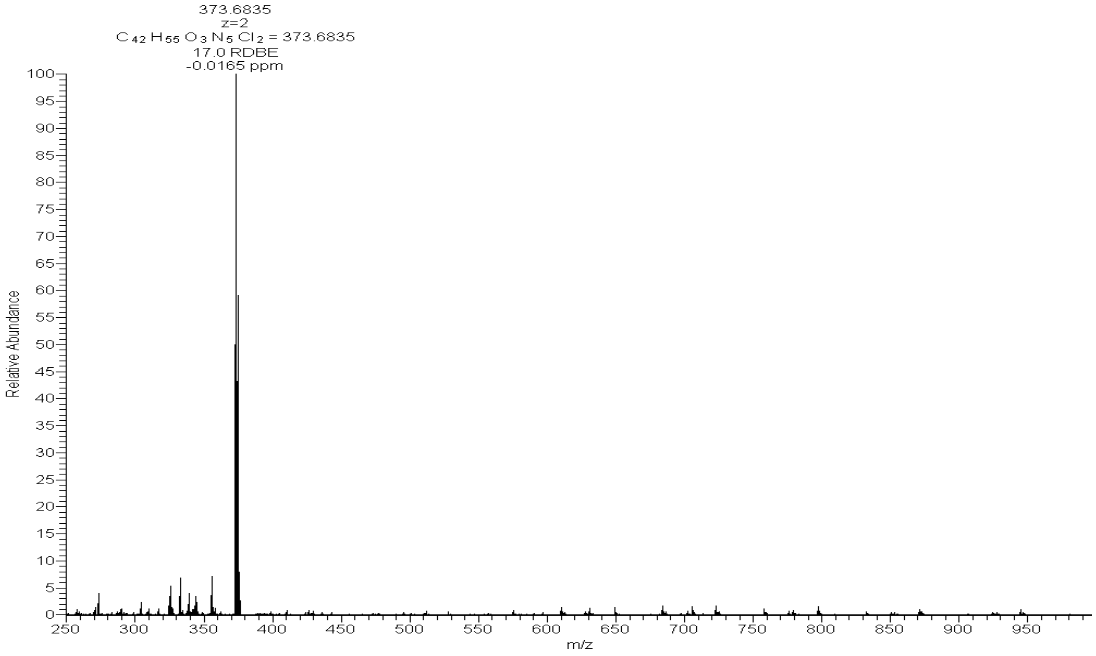

Figure S47. HR ESI-MS spectrum of compound **6**.

10.- References

[1] Roibu, A.; Fransen, S.; Leblebici, M. E.; Meir, G.; Gerven, T. V.; Kuhn, S., An accessible visible-light actinometer for the determination of photon flux and optical pathlength in flow photo microreactors, *Sci. Rep.* **2018**, *8*, 5421.

[2] Maafi, M.; The potential of AB(1Φ) systems for direct actinometry. Diarylethenes as successful actinometers for the visible range.*Phys. Chem. Chem. Phys*. **2010**, *12*, 13248–13254.

[3] Maafi, M.; Brown, R. G., The kinetic model for AB(1Φ) systems A closed-form integration of the differential equation with a variable photokinetic factor. *J. Photochem. Photobiol. A 187*, **2007**, 319–324.

[4] Sumi, T.; Takagi, Y.; Yagi, A.; Morimoto, M.; Irie, M. Photoirradiation wavelength dependence of cycloreversion quantum yields of diarylethenes. *Chem. Commun.* **2014**, *50*, 3928–3930.
